# Supplementary material for: The transcriptional co‐activator Yap1 promotes adult hippocampal neural stem cell activation
Source: EMBO J. 2023 Apr 21;42(11):e110384. doi: 10.15252/embj.2021110384 (PMC10233373; doi:10.15252/embj.2021110384)
Supplement: Supplementary file 1 — Appendix S1 [file EMBJ-42-e110384-s005.pdf]

APPENDIX MATERIALS

Contents

Appendix Figures S1-S4 and Appendix Figure Legends

Appendix Fig S1

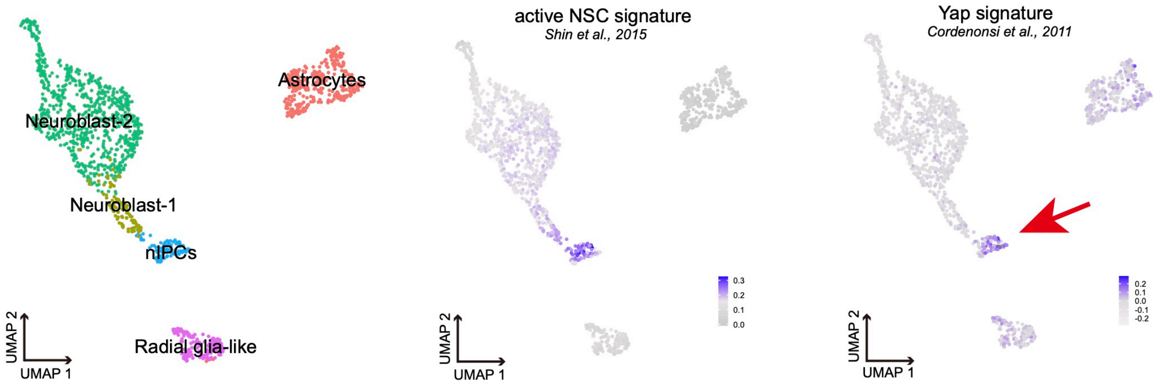

Appendix Fig S1. Yap1 signature is enriched in the proliferative neural progenitors of the mouse hippocampus.

Uniform Manifold Approximation and Projection (UMAP) of single cell transcriptome from published data set comprising NSCs and their early progeny isolated from the dentate gyrus of the adult mouse hippocampus. Yap1 signature is enriched in the same cell population as the active NSC signature. Arrow indicates Yap1 signature in the nIPCs (neural intermediate progenitor cells).

Appendix Fig S2

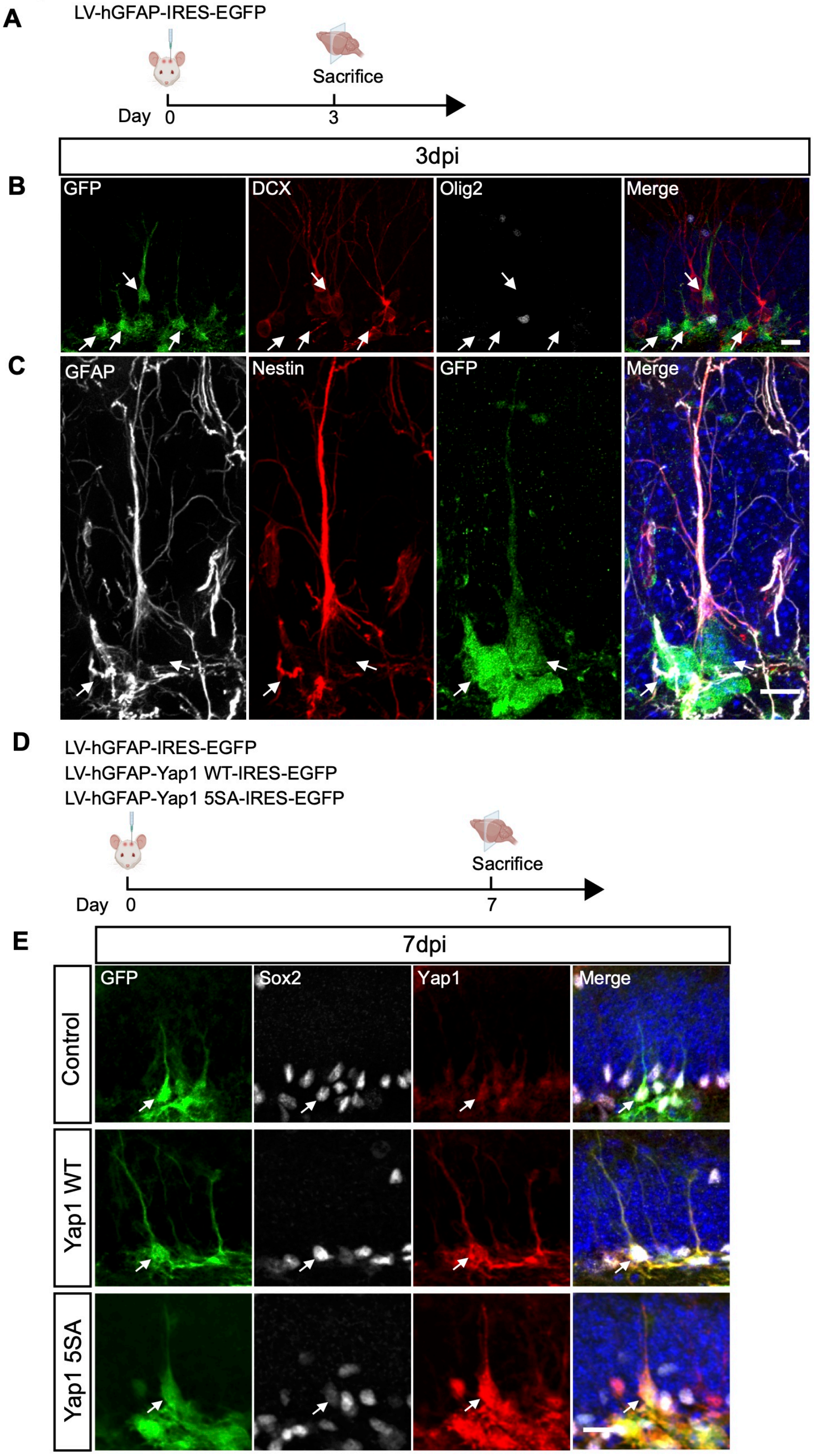

Appendix Fig S2. Targeting of adult NSCs *in vivo* with lentivirus under the control of hGFAP gene regulatory sequence.

A. Schematic diagram of experimental design. P60 mice were analyzed at 3 days after control lentivirus injection.

B. Immunofluorescence for GFP, DCX and Olig2 in coronal brain sections. Arrows indicate adult RGLs in the DG.

C. Immunofluorescence for GFP, Nestin and GFAP in coronal brain sections. Arrows indicate GFAP, Nestin and GFP positive adult RGLs in the DG.

D. Schematic diagram of experimental design. P60 mice were analyzed at 7 days after lentivirus injection of control, wild type Yap1 and Yap1-5SA.

E. Immunofluorescence for GFP, Sox2 and Yap1 in coronal brain sections. Arrows indicate the expression of Yap1 in adult RGLs.

Data information: Scale bars: 20 μm.

Appendix Fig S3

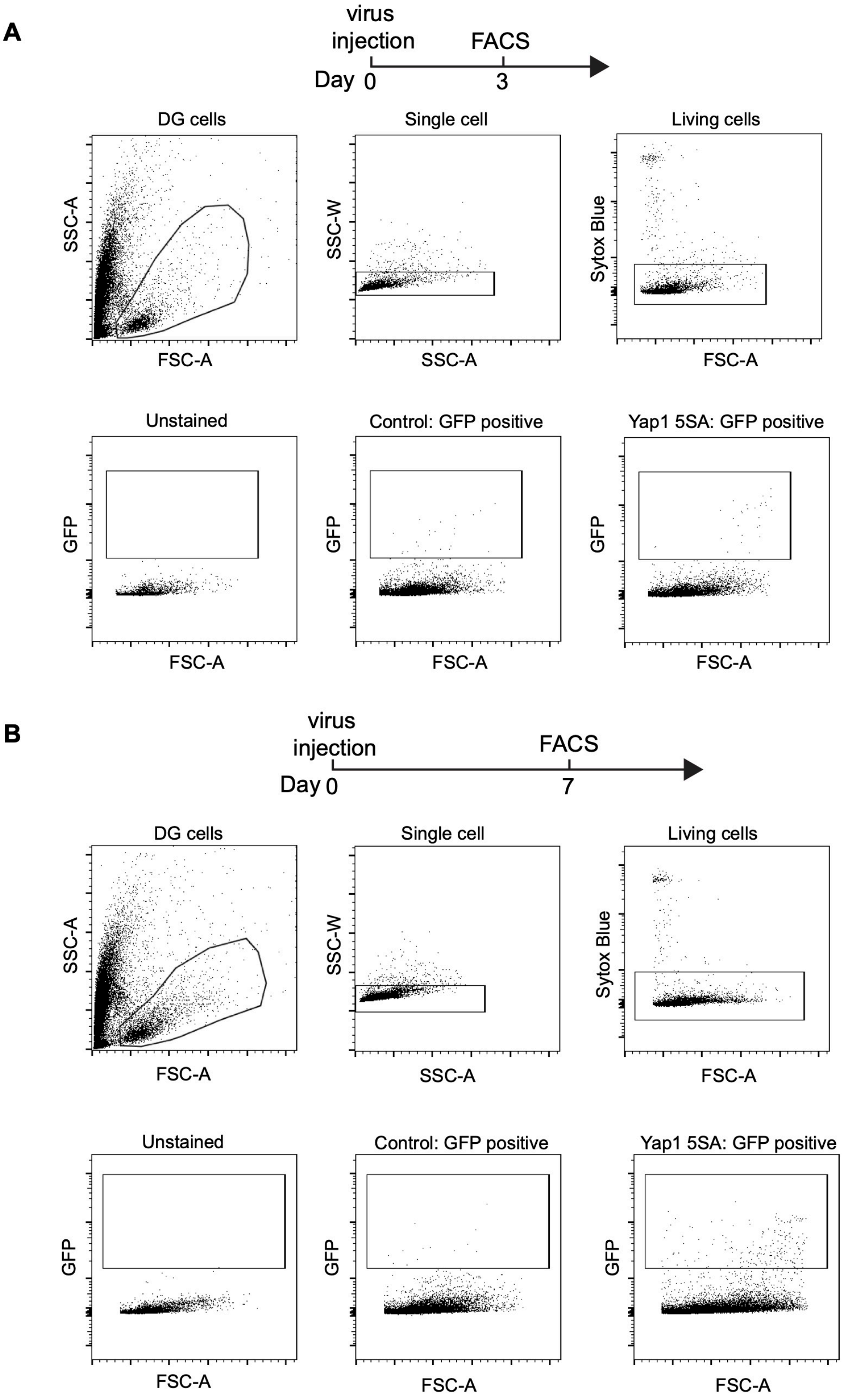

Appendix Fig S3. Gating strategy for FACS at 3 dpi and 7 dpi.

A-B. FACS strategy to obtain GFP positive cells (transduced cells by LV-hGFAP-IRES-EGFP or LV-hGFAP-Yap1-5SA-IRES-EGFP). First gate uses stringent FSC/SSC gating to exclude cellular debris; second excludes cell aggregates to obtain single cells; third excludes dead and dying cells by Sytox Blue; fourth excludes GFP negative cells. GFP positive cells were collected in both control and Yap1 overexpression group at 3dpi and 7dpi.

Appendix Fig S4

A Differentially expressed genes for each cluster after removing dying cells, microglia and oligodendroglial cells

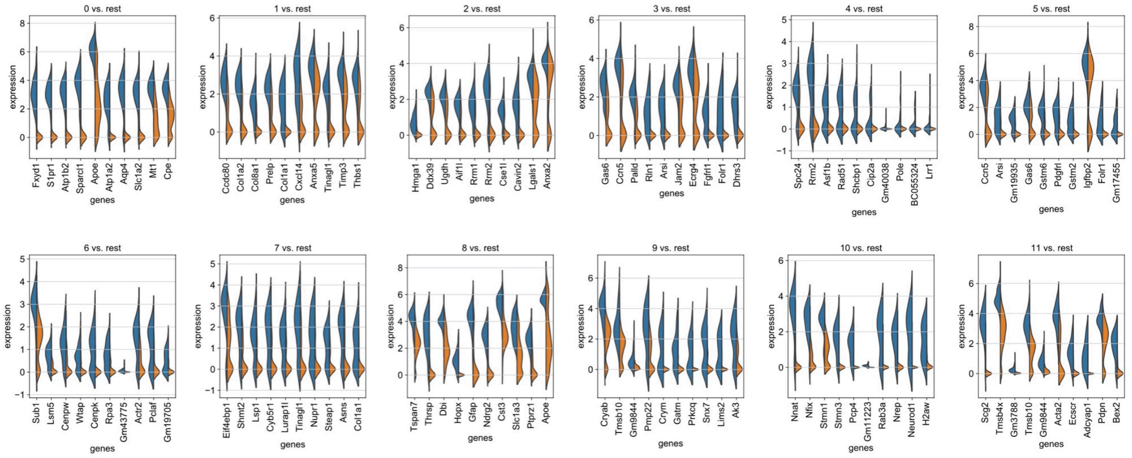

B experimental groups

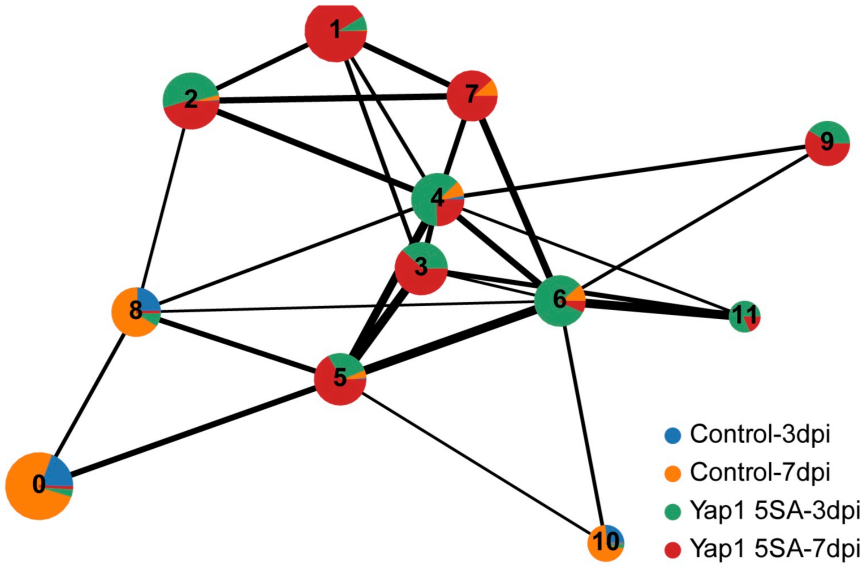

Appendix Fig S4. Re-clustering of the single cell RNA sequencing data after removal of dying cells, microglia and oligodendroglial cells

A. Top 10 markers for each cluster after re-clustering the data.

B. Composition of experimental groups in each cluster are indicated in the plot.
