# Supplementary material for: The transcriptional co‐activator Yap1 promotes adult hippocampal neural stem cell activation
Source: EMBO J. 2023 Apr 21;42(11):e110384. doi: 10.15252/embj.2021110384 (PMC10233373; doi:10.15252/embj.2021110384)
Supplement: Supplementary file 2 — Expanded View Figures PDF [file EMBJ-42-e110384-s004.pdf]

Expanded View Figures

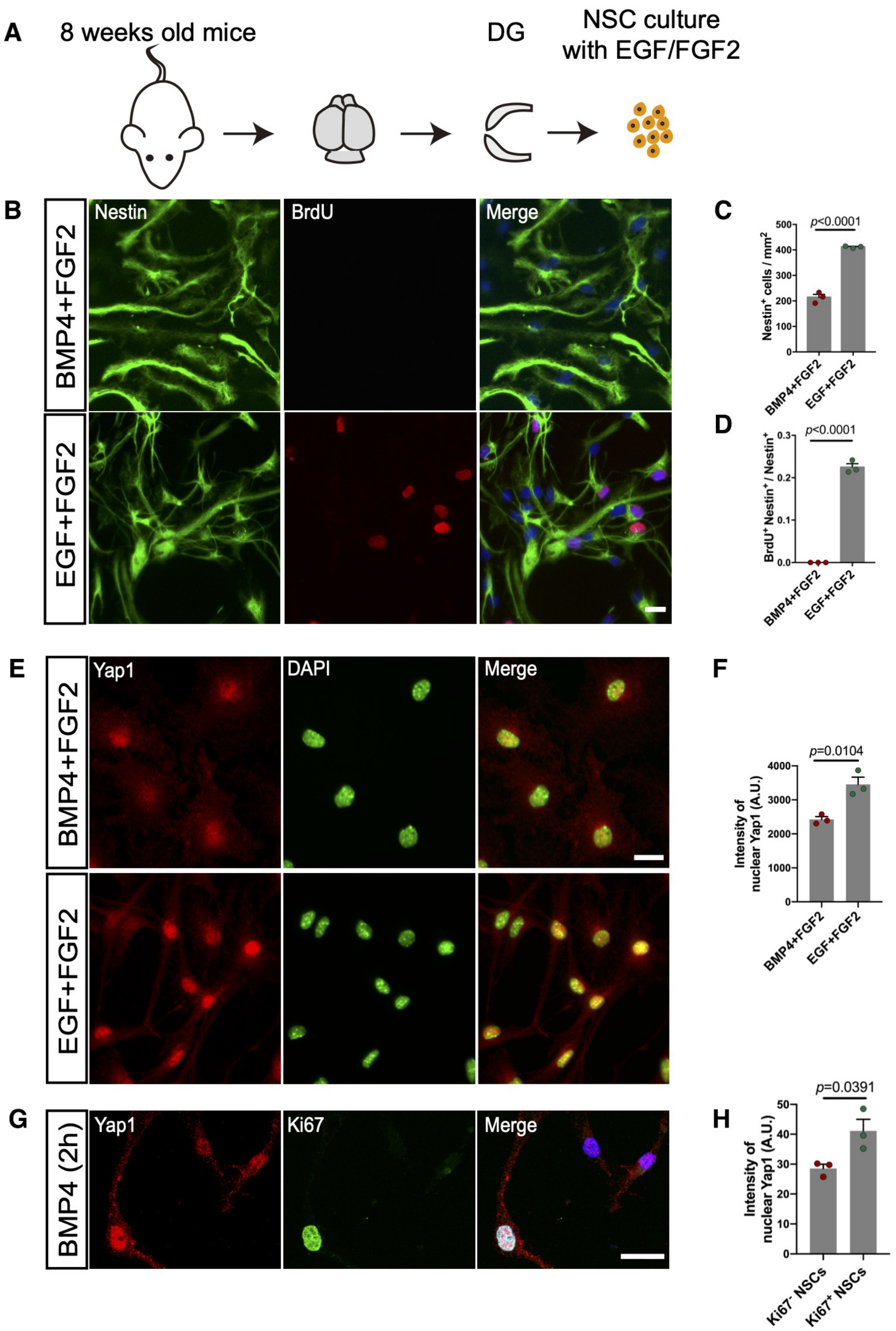

Figure EV1.

Figure EV1. Nuclear Yap1 level is increased upon adult NSCs activation.

A Schematic diagram of adult hippocampus derived NSCs culture.

B Cultured adult NSCs were treated with EGF + FGF2 (proliferative condition) or BMP4 + FGF2 (quiescent condition). Immunofluorescence for Nestin and BrdU indicates that BMP4 + FGF2 treatment efficiently blocks the cell division of adult NSCs, as evidenced by the fact that they did not incorporate the BrdU administered during the last 6 h prior to fixation.

C, D Quantification of the data in (B). No BrdU-positive cells were found in quiescent condition and resulted in a reduction of NSCs density. n = 3 (biological replicates) independent experiments.

E Immunofluorescence for Yap1 and DAPI in quiescent and proliferative NSCs.

F Quantification of the nuclear Yap1 intensity in (E). Nuclear Yap1 level increases upon activation of quiescent NSCs. n = 3 (biological replicates, 300 cells from three independent experiments) in proliferative condition, n = 3 (biological replicates, 249 cells from three independent experiments) in quiescent condition.

G Cultured adult NSCs were treated with BMP4 + FGF2 for 2 h. Immunofluorescence for Yap1 and Ki67 in proliferative and nonproliferative NSCs.

H Quantification of the data in (G). Proliferative NSCs (Ki67-positive) enriched higher level of nuclear Yap1. n = 3 (biological replicates, 85 Ki67-positive cells and 91 Ki67-negative cells from 3 independent experiments).

Data information: Data are represented as mean ± SEM. Unpaired Student's t-test. Scale bars: 20 μm.

Figure EV2. Conditional knockout Yap1 in adult NSCs at 30 dpi.

A Immunofluorescence for GFP (recombination reporter), GFAP, and Mcm2 in control and Yap1 cKO RGLs at 30 days after tamoxifen administration. Arrows indicate Mcm2-positive, proliferating RGLs.

B Quantification of the data in (A). Loss of Yap1 does not induce significant changes in the number of proliferative RGLs or the overall number of RGLs. n = 3 mice (biological replicates) for control group, n = 4 mice (biological replicates) for Yap1 cKO group.

C, D Immunofluorescence for GFP (recombination reporter) and DCX in control and Yap1 cKO RGLs at 30 and 60 days after tamoxifen administration. Arrows indicate DCX-positive immature neurons.

E, F Quantification of the data in (C and D). Loss of Yap1 does not induce significant changes in the number of immature neurons. 30 dpi group (E): n = 3 mice for control group, n = 4 mice (biological replicates) for Yap1 cKO group; 60 dpi group (F): n = 4 mice (biological replicates) for both control group and Yap1 cKO group.

G Immunofluorescence for GFP (recombination reporter), GFAP, and Yap1 in control and Yap1 cKO RGLs at 60 days after tamoxifen administration. Quantification of Yap1 intensity in recombined RGLs. Yap1 levels are significantly decreased in Yap1 cKO RGLs at 60 dpi. n = 4 (23 cells from four mice) in control group, n = 4 (21 cells from four mice) in Yap1 cKO group.

H Schematic diagram of experimental design.

I Immunofluorescence for GFP and EdU indicates that loss of Yap1 efficiently blocks the cell division of adult NSCs, as evidenced by the fact that they did not incorporate the EdU administered during the last 4 h prior to fixation. Quantification shown in the right panel. n = 3 independent experiments (biological replicates).

Data information: Data are represented as mean ± SEM. Unpaired Student's t-test. Scale bars: 20 μm in (A), (C), and (I); 10 μm in (D); 5 μm in (G).

 © 2023 The Authors

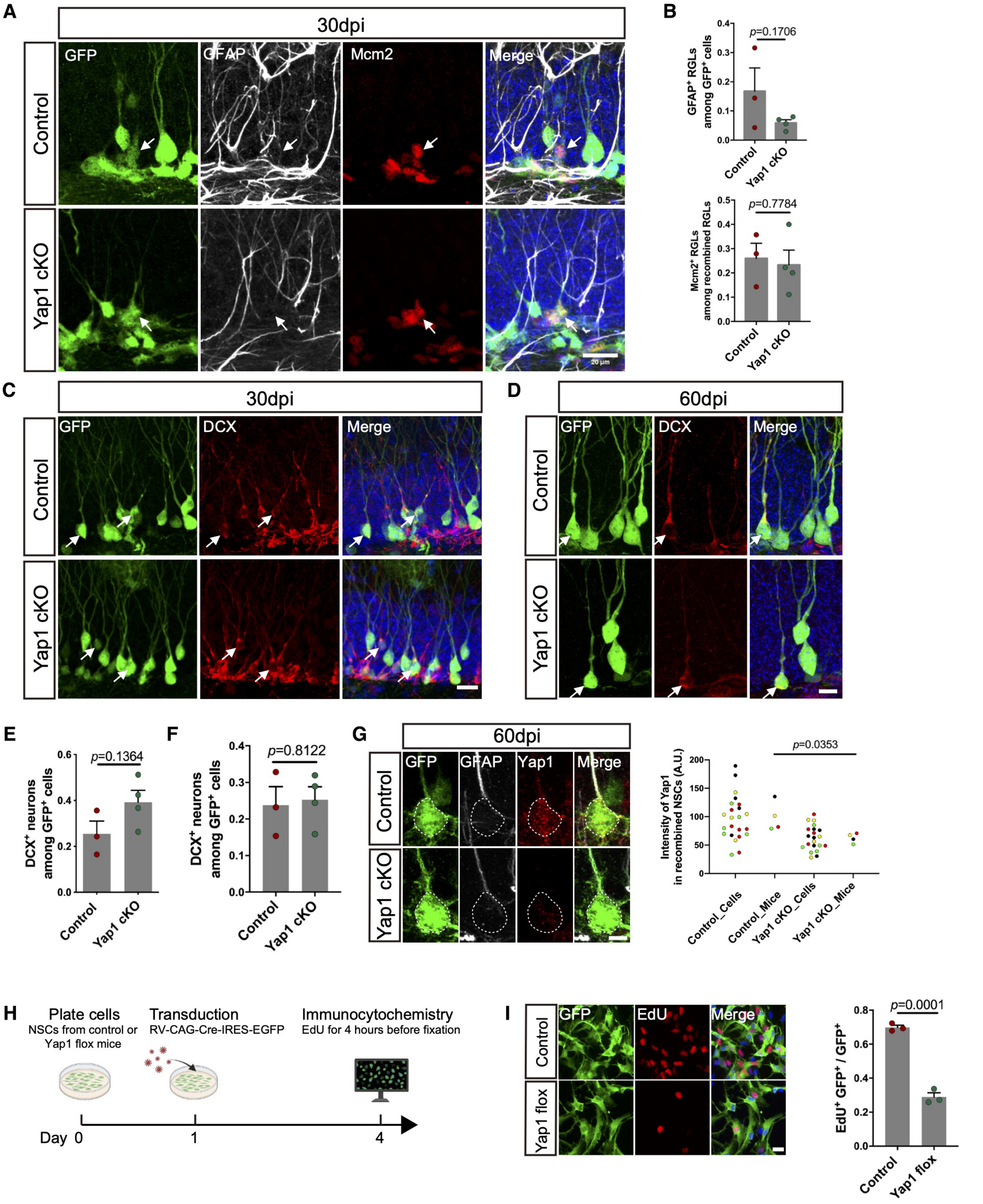
A
30dpi
GFP
GFAP
Mcm2
Merge
Control
Yap1 cKO
20 μm
B
p=0.1706
GFAP+ RGLs among GFP+ cells
Control
Yap1 cKO
p=0.7784
Mcm2+ RGLs among recombined RGLs
C
30dpi
GFP
DCX
Merge
D
60dpi
E
p=0.1364
DCX+ neurons among GFP+ cells
F
p=0.8122
G
60dpi
Yap1
p=0.0353
Intensity of Yap1 in recombined NSCs (A.U.)
Control_Cells
Control_Mice
Yap1 cKO_Cells
Yap1 cKO_Mice
H
Plate cells
NSCs from control or Yap1 flox mice
Transduction
RV-CAG-Cre-IRES-EGFP
Immunocytochemistry
EdU for 4 hours before fixation
Day 0
1
4
I
EdU
Yap1 flox
p=0.0001
EdU+ GFP+ / GFP+

Figure EV2.

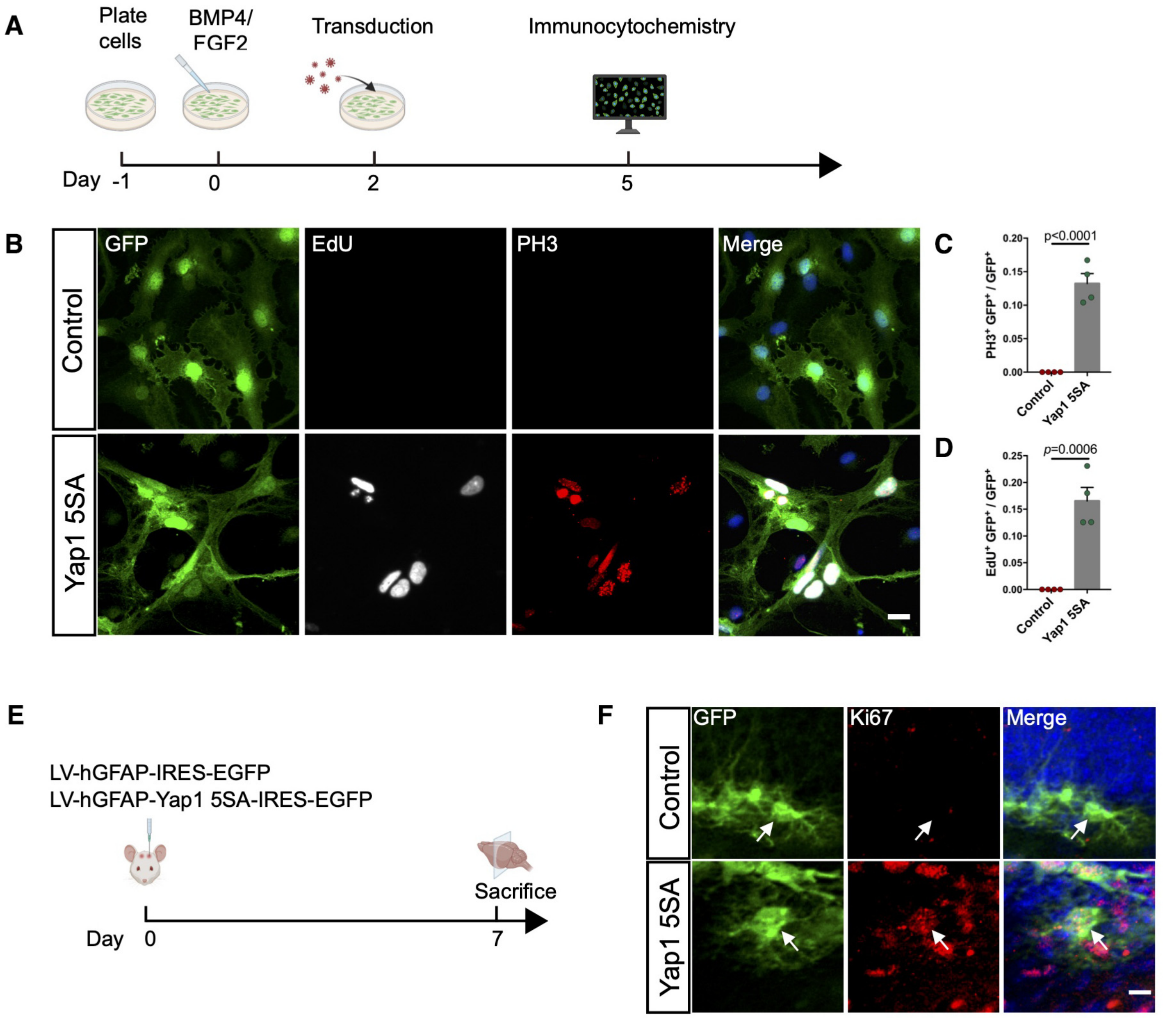

Figure EV3. Overexpression of Yap1-5SA induces the proliferation of quiescent adult NSCs *in vitro* and astrocytes *in vivo*.

A Schematic diagram of the experimental design for the analysis of the effect of Yap1 overexpression in quiescent NSCs *in vitro*.

B Immunofluorescence for GFP, EdU (administered 6 h before fixation), and PH3 (to identify proliferative cells) in control and Yap1-5SA overexpressing NSCs.

C, D Quantification of the data in (B). Yap1-5SA induces the proliferation of quiescent NSCs *in vitro*. $n = 4$ (biological replicates) independent cultures for each group.

E Schematic diagram of the experimental design for the analysis of the effect of Yap1 overexpression in astrocytes in hilus.

F Immunofluorescence for GFP and Ki67 in coronal brain sections. Arrows indicate astrocytes in hilus.

Data information: Data are represented as mean ± SEM. Unpaired Student's *t*-test. Scale bars: 20 μm.

Figure EV4. Clustering of single cell RNA-sequencing data and cell-type identification based on differential gene expression analysis.

A Experimental groups and 13 different cell clusters.

B Number of transcripts in both control and Yap1-5SA-expressing cells.

C-H Markers for each cell type including NSCs, astrocytes, dying cells, microglia, oligodendroglial cells, and neurons.

I Identification of top 10 markers for each cluster via differential gene expression analysis.

J Expression levels of cell cycle-related genes at 3 and 7 days after lentivirus injection.

© 2023 The Authors

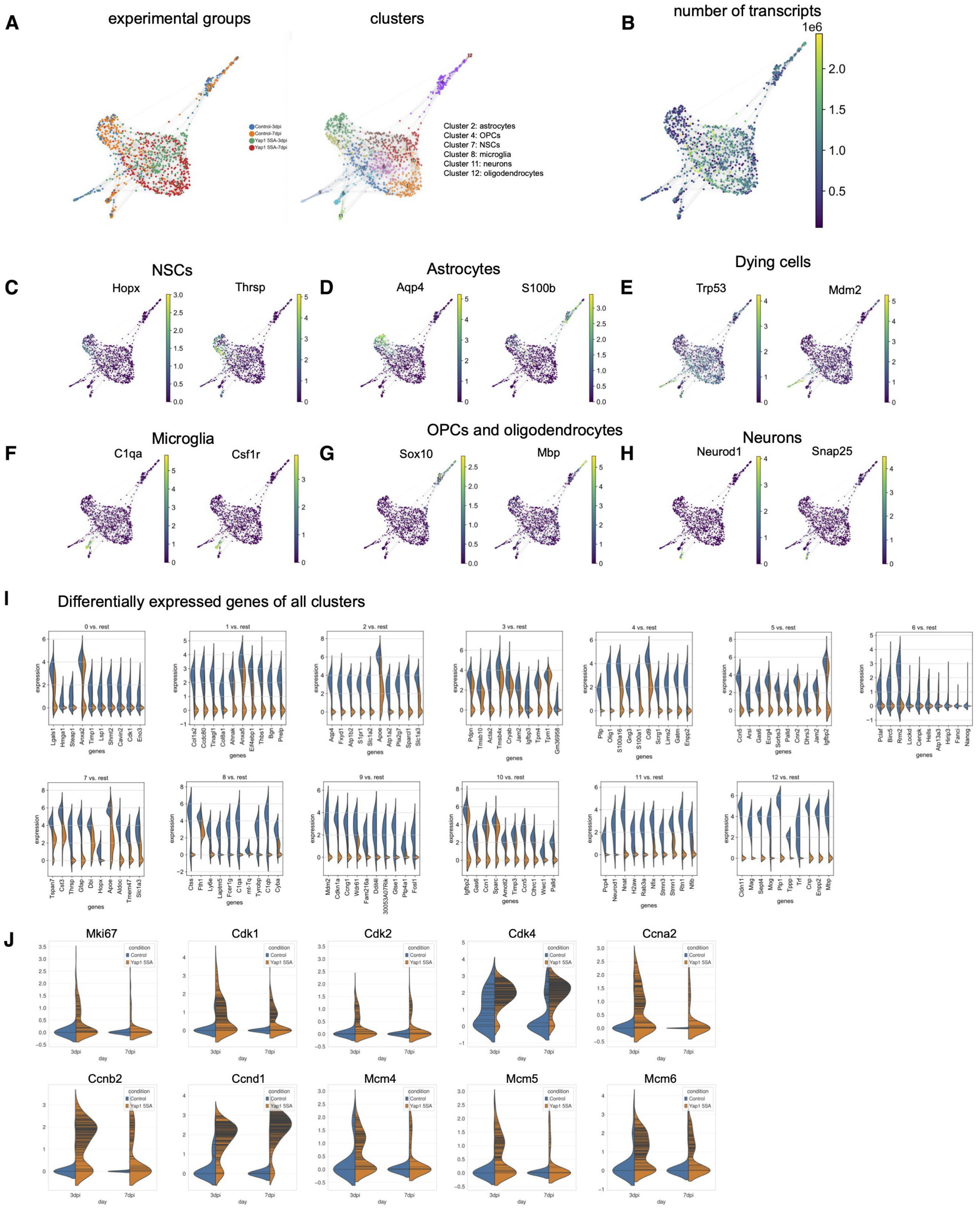
A
experimental groups
clusters
Control-3dpi
Control-7dpi
Yap1 5SA-3dpi
Yap1 5SA-7dpi
Cluster 2: astrocytes
Cluster 4: OPCs
Cluster 7: NSCs
Cluster 8: microglia
Cluster 11: neurons
Cluster 12: oligodendrocytes
B
number of transcripts
1e6
C
NSCs
Hopx
Thrsp
D
Astrocytes
Aqp4
S100b
E
Dying cells
Trp53
Mdm2
F
Microglia
C1qa
Csf1r
G
OPCs and oligodendrocytes
Sox10
Mbp
H
Neurons
Neurod1
Snap25
I
Differentially expressed genes of all clusters
J
Mki67
Cdk1
Cdk2
Cdk4
Ccna2
Ccnb2
Ccnd1
Mcm4
Mcm5
Mcm6
condition
Control
Yap1 5SA
3dpi
7dpi
day

Figure EV4.

Figure EV5. Overexpression of Yap1-5SA in adult NSCs for 30 days *in vivo*.

A Schematic diagram of experimental design. P60 mice were analyzed at 30 days after lentivirus injection of control, wild-type Yap1 and Yap1-5SA.
B Immunofluorescence for GFP and Sox2 in control conditions or overexpression of wild-type Yap1 or Yap1-5SA 30 days after lentivirus injection.
C High magnification images from immunofluorescence in (B). Lower panel shows quantification of Sox2-positive cells among GFP-positive cells in (C). $n = 4$ mice (biological replicates) in control and Yap1-5SA group; $n = 3$ mice (biological replicates) in Yap1 WT group.
D Immunofluorescence for GFP and NeuN in control conditions or overexpression of wild-type Yap1 or Yap1-5SA 30 days after lentivirus injection.
E Immunofluorescence for GFP and S100 beta (astrocyte marker) in Yap1-5SA overexpressing mice at 30 days after lentivirus injection.

Data information: Data are represented as mean ± SEM. One-way ANOVA. Scale bars: 100 μm in (B). 10 μm in (C), (D), and (F).

 © 2023 The Authors

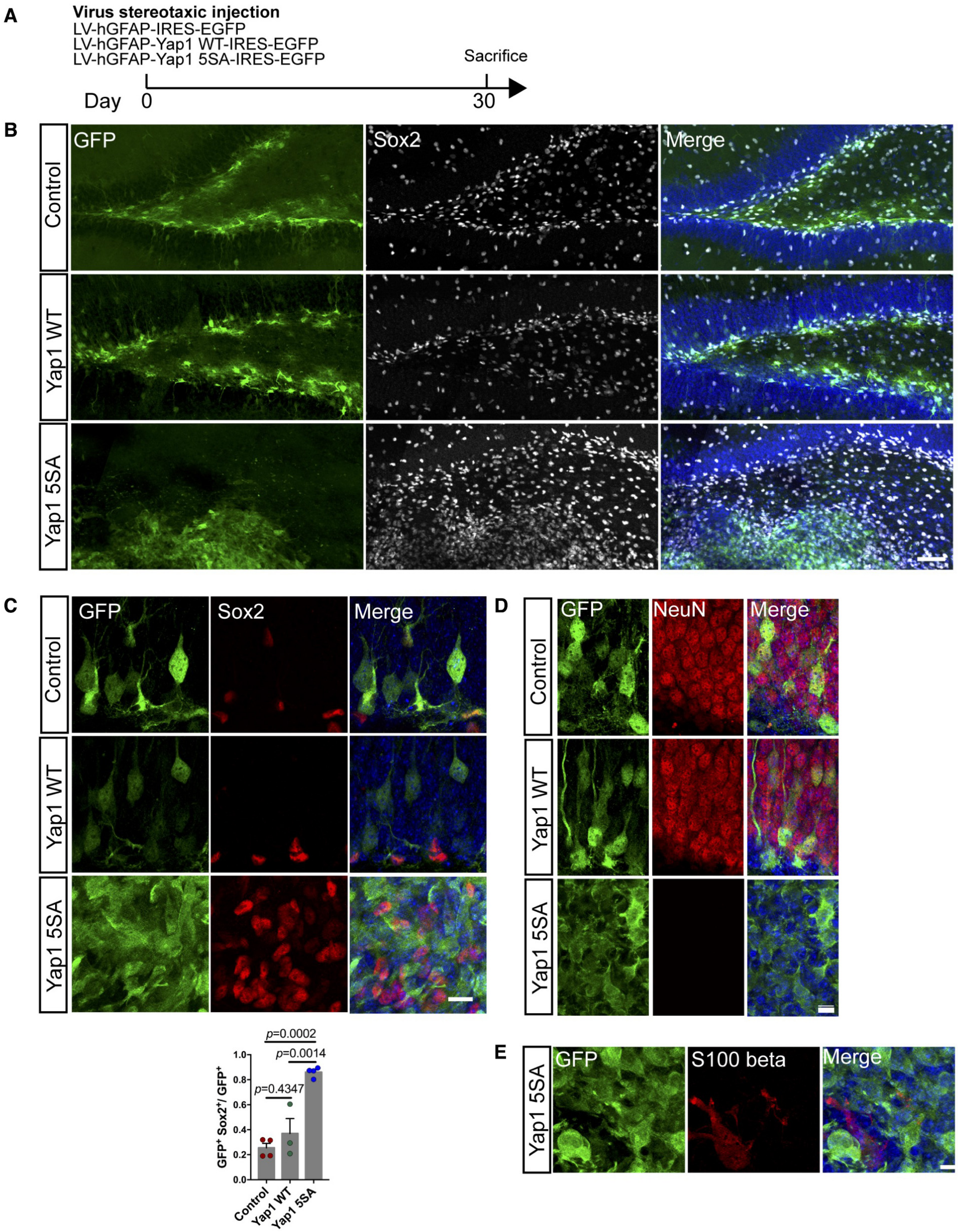
A
Virus stereotaxic injection
LV-hGFAP-IRES-EGFP
LV-hGFAP-Yap1 WT-IRES-EGFP
LV-hGFAP-Yap1 5SA-IRES-EGFP
Sacrifice
Day 0
30
B
Control
Yap1 WT
Yap1 5SA
GFP
Sox2
Merge
C
GFP
Sox2
Merge
Control
Yap1 WT
Yap1 5SA
D
GFP
NeuN
Merge
Control
Yap1 WT
Yap1 5SA
p=0.0002
p=0.0014
p=0.4347
GFP+ Sox2+/ GFP+
1.0
0.8
0.6
0.4
0.2
0.0
Control
Yap1 WT
Yap1 5SA
E
Yap1 5SA
GFP
S100 beta
Merge

Figure EV5.
