# Supplementary material for: The transcriptional co‐activator Yap1 promotes adult hippocampal neural stem cell activation
Source: EMBO J. 2023 Apr 21;42(11):e110384. doi: 10.15252/embj.2021110384 (PMC10233373; doi:10.15252/embj.2021110384)
Supplement: Supplementary file 3 — Source Data for Expanded View [file EMBJ-42-e110384-s001.zip › Fig EV3B control/READ ME Figure EV3B control.docx]

**Figure EV3B control**

Confocal image of adult NSCs cultured in quiescent conditions and transduced with control virus (LV-hGFAP-IRES-EGFP). Cells were stained for:

- Channel 1 (C1-Fig EV3B control): Phospho-Histone H3 (PH3)
- Channel 2 (C2-Fig EV3B control): EdU
- Channel 3 (C3-Fig EV3B control): GFP
- Channel 4 (C4-Fig EV3B control): DAPI
