# Supplementary figures and images for: The transcriptional co‐activator Yap1 promotes adult hippocampal neural stem cell activation
Source: EMBO J. 2023 Apr 21;42(11):e110384. doi: 10.15252/embj.2021110384 (PMC10233373; doi:10.15252/embj.2021110384)

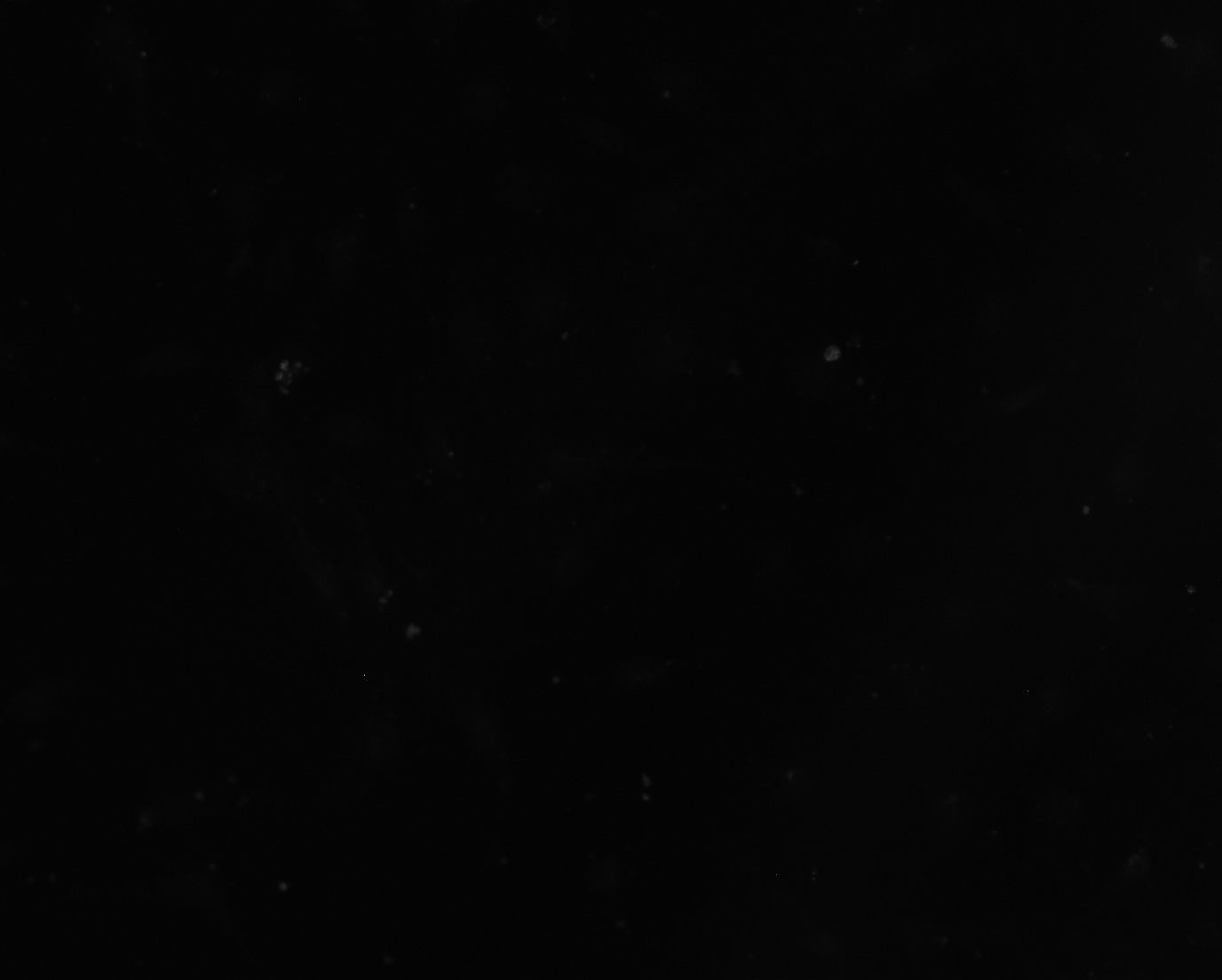

Supplement: Supplementary file 3 — Source Data for Expanded View [file EMBJ-42-e110384-s001.zip › Fig EV3B control/C1-Fig EV3B control.tif]

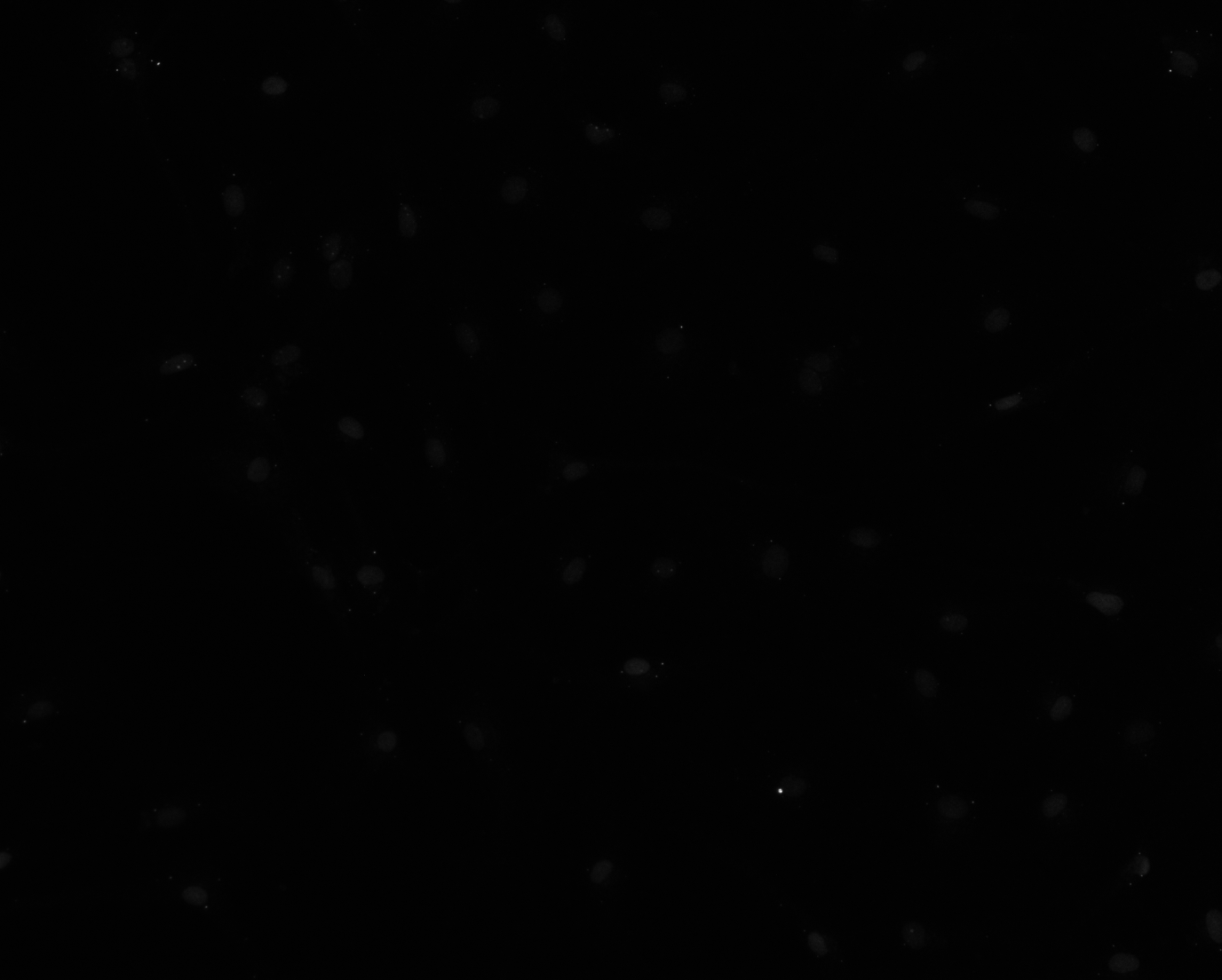

Supplement: Supplementary file 3 — Source Data for Expanded View [file EMBJ-42-e110384-s001.zip › Fig EV3B control/C2-Fig EV3B control.tif]

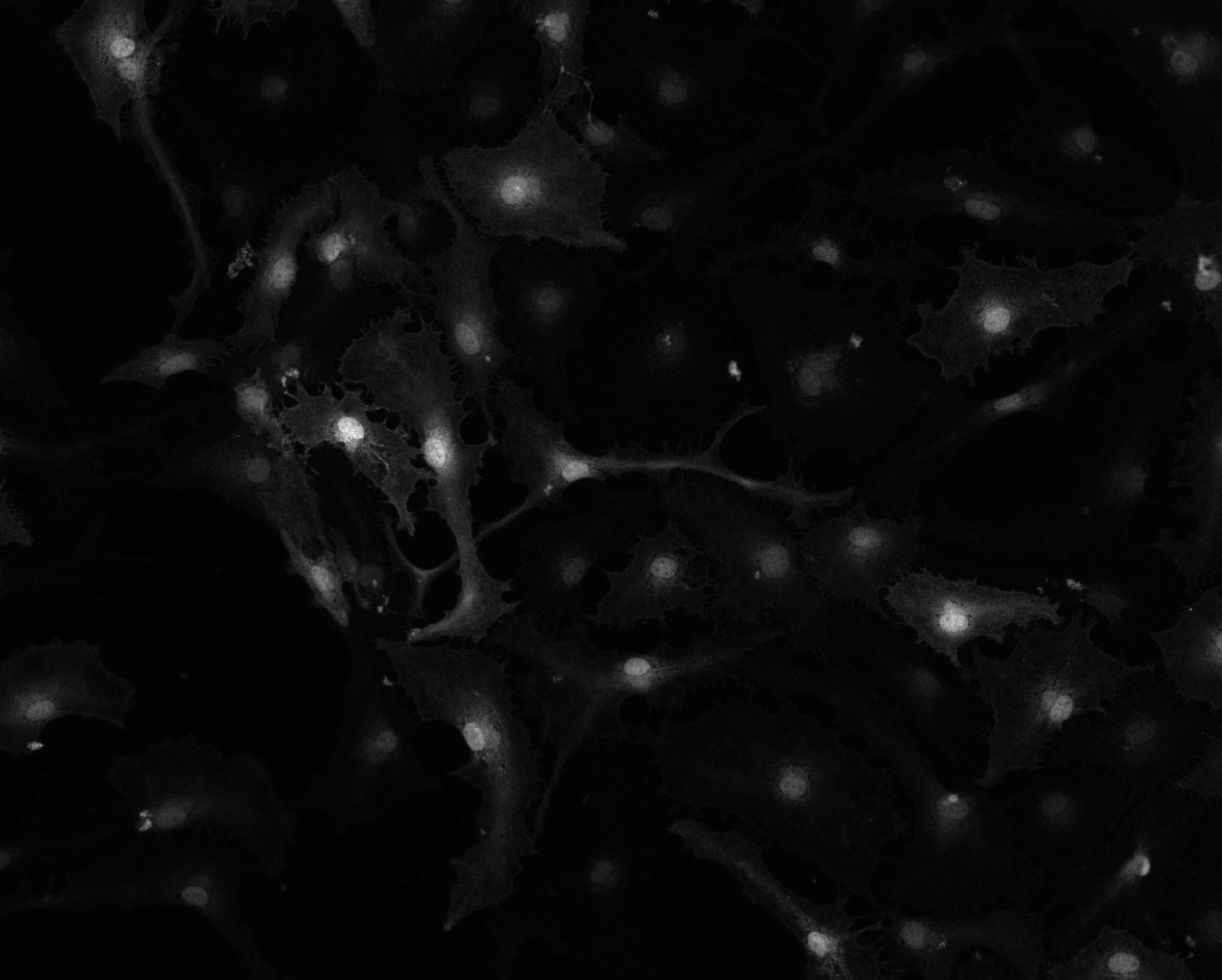

Supplement: Supplementary file 3 — Source Data for Expanded View [file EMBJ-42-e110384-s001.zip › Fig EV3B control/C3-Fig EV3B control.tif]

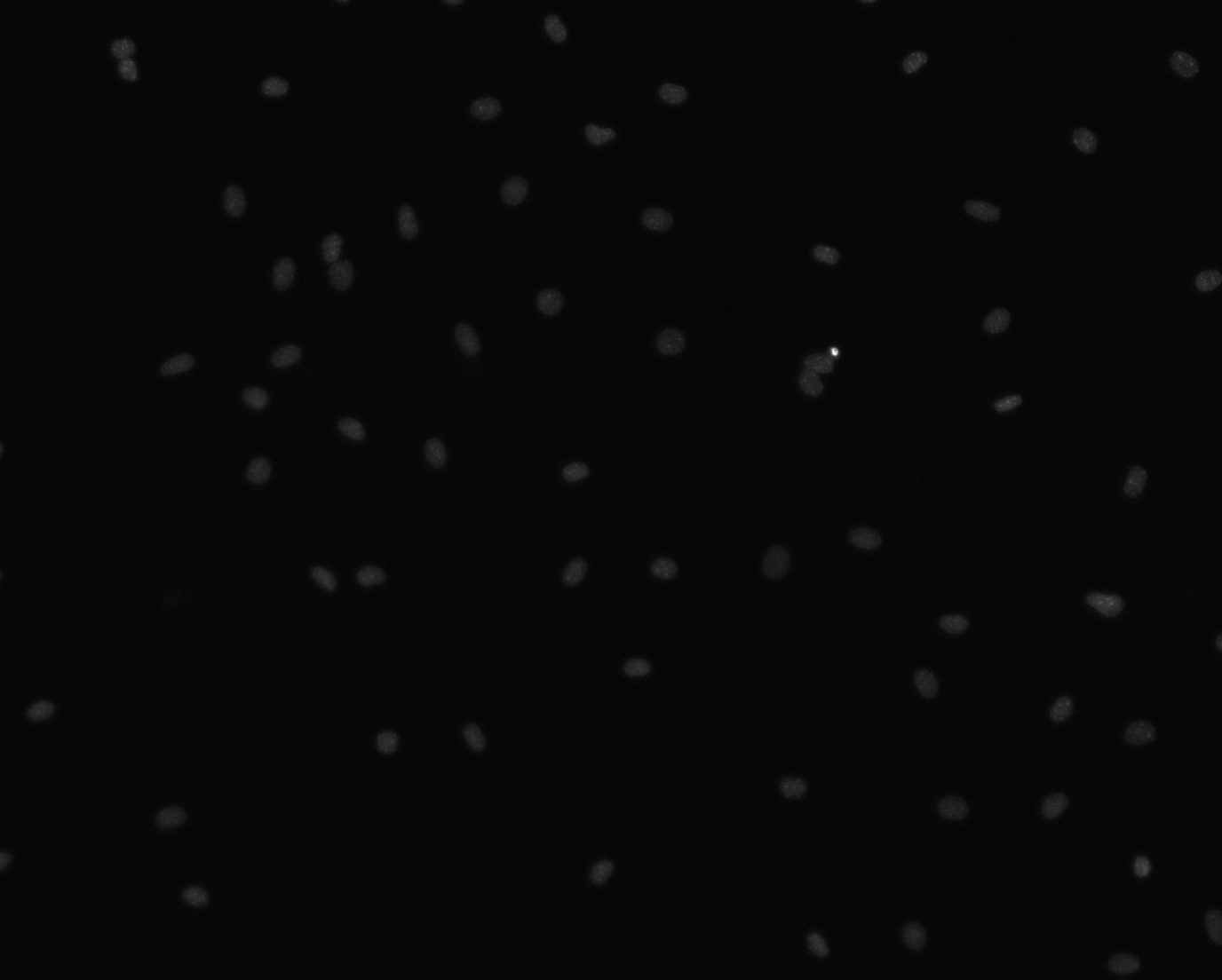

Supplement: Supplementary file 3 — Source Data for Expanded View [file EMBJ-42-e110384-s001.zip › Fig EV3B control/C4-Fig EV3B control.tif]

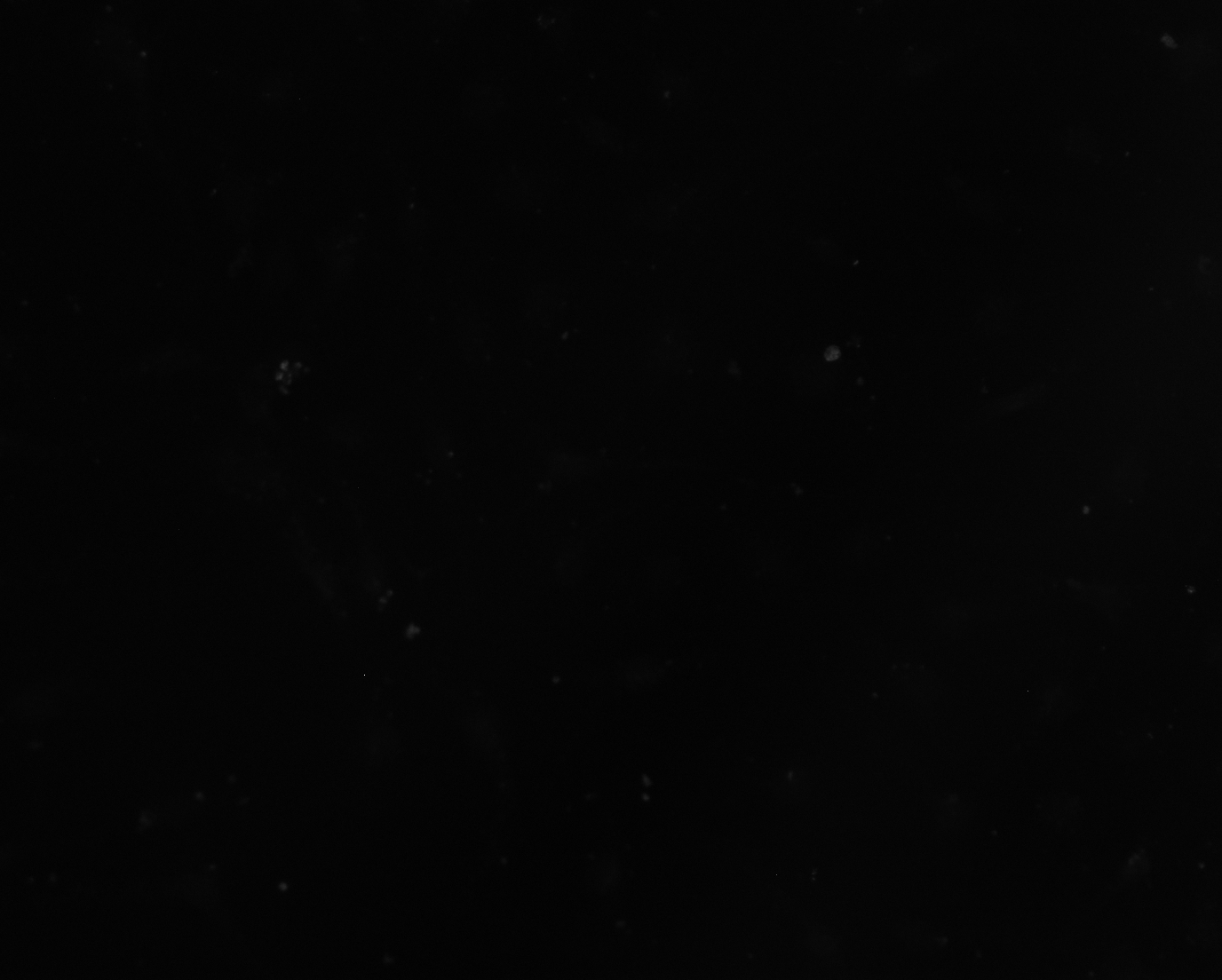

Supplement: Supplementary file 3 — Source Data for Expanded View [file EMBJ-42-e110384-s001.zip › Fig EV3B control/Fig EV3B control.tif]

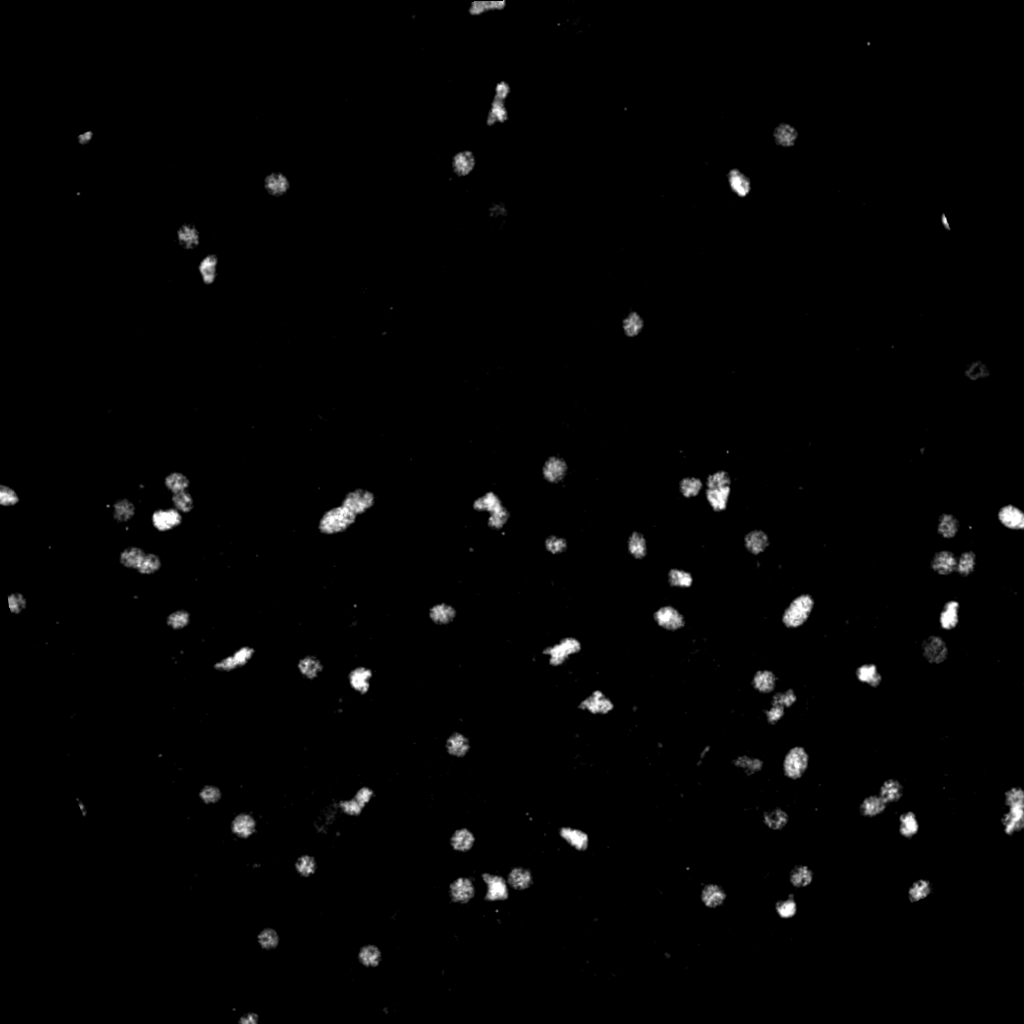

Supplement: Supplementary file 5 — Source Data for Figure 1 [file EMBJ-42-e110384-s008.zip › Figure 1 Source Data/D/Fig1D.tif]

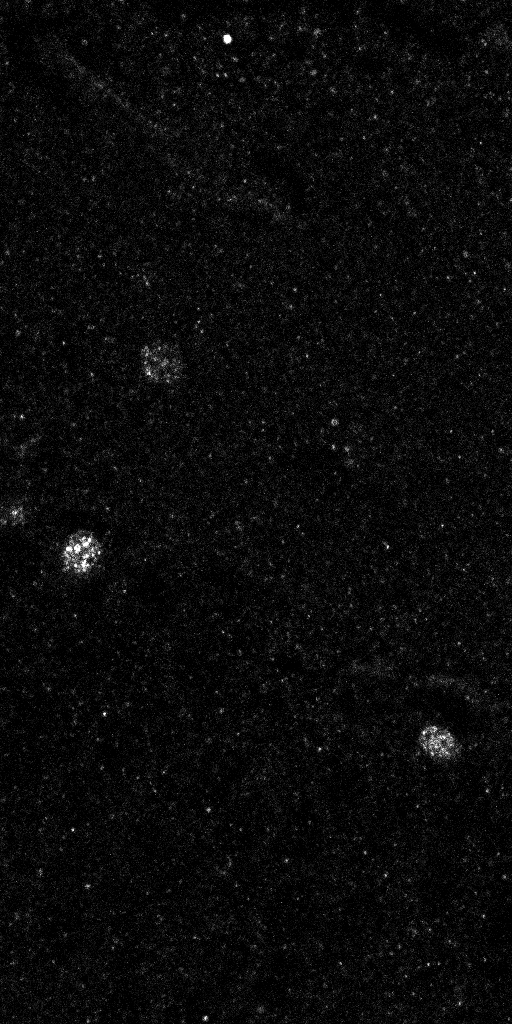

Supplement: Supplementary file 5 — Source Data for Figure 1 [file EMBJ-42-e110384-s008.zip › Figure 1 Source Data/E/Yap1-active NSCs.tif]

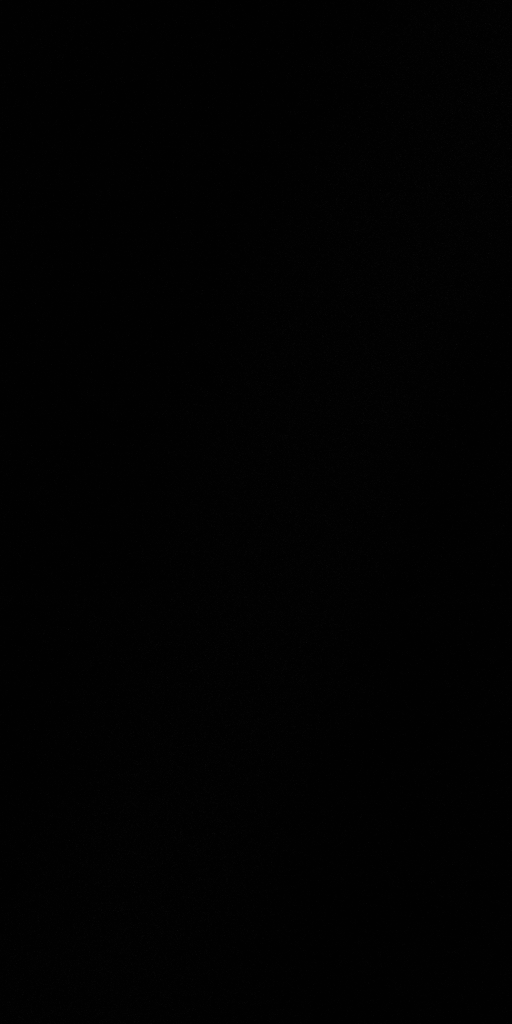

Supplement: Supplementary file 5 — Source Data for Figure 1 [file EMBJ-42-e110384-s008.zip › Figure 1 Source Data/E/Yap1-NRPs.tif]

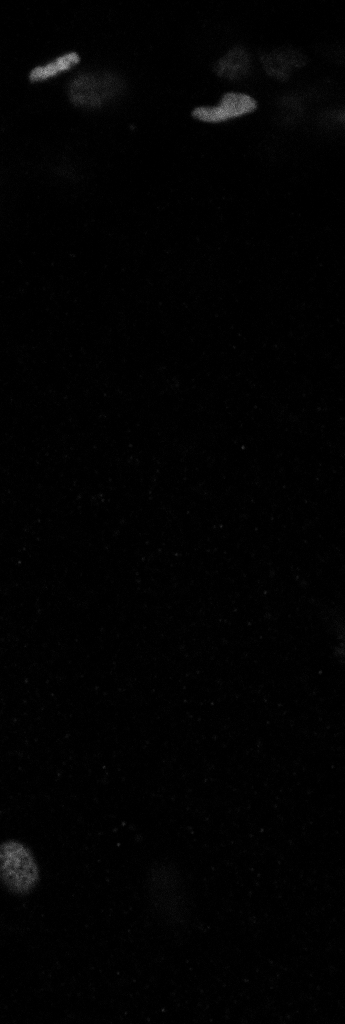

Supplement: Supplementary file 5 — Source Data for Figure 1 [file EMBJ-42-e110384-s008.zip › Figure 1 Source Data/E/Yap1-quiescent NSCs.tif]

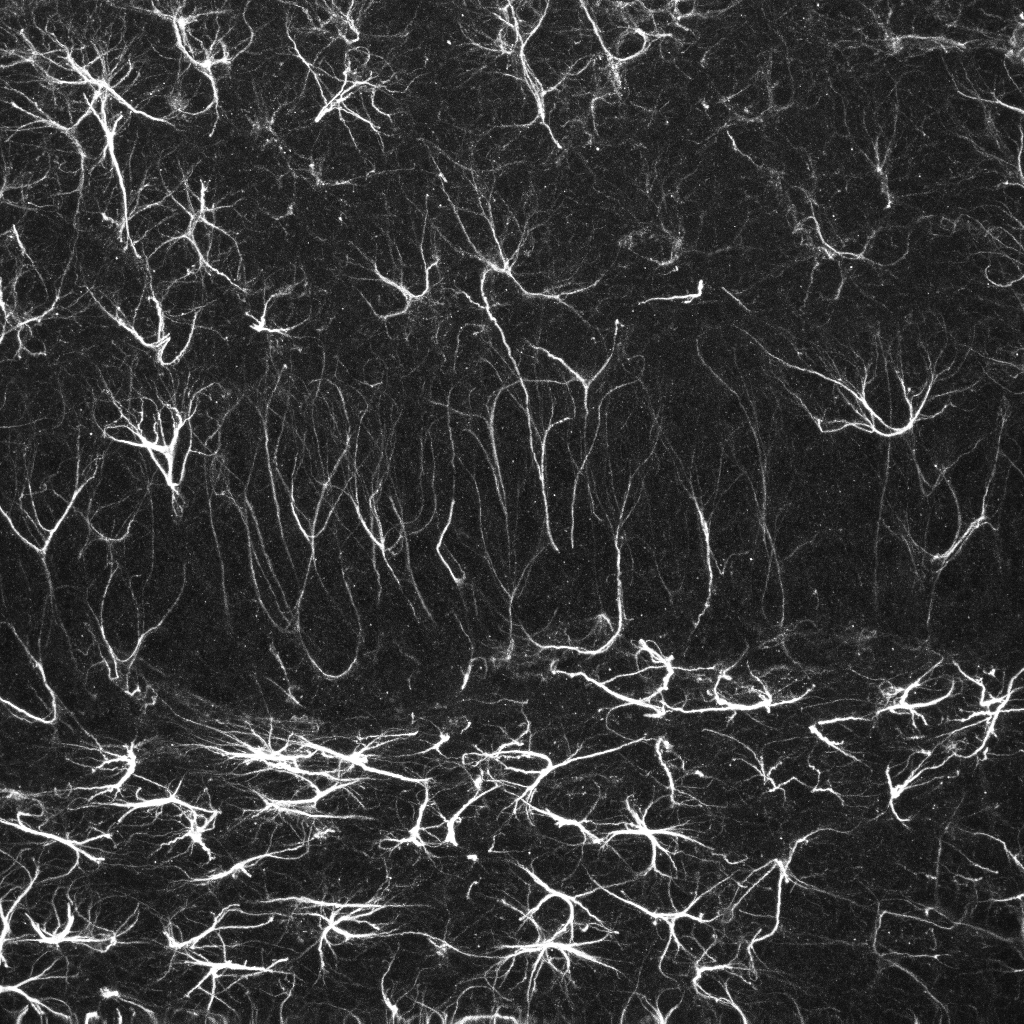

Supplement: Supplementary file 6 — Source Data for Figure 2 [file EMBJ-42-e110384-s006.zip › Figure 2 Source Data/G/Control.tif]

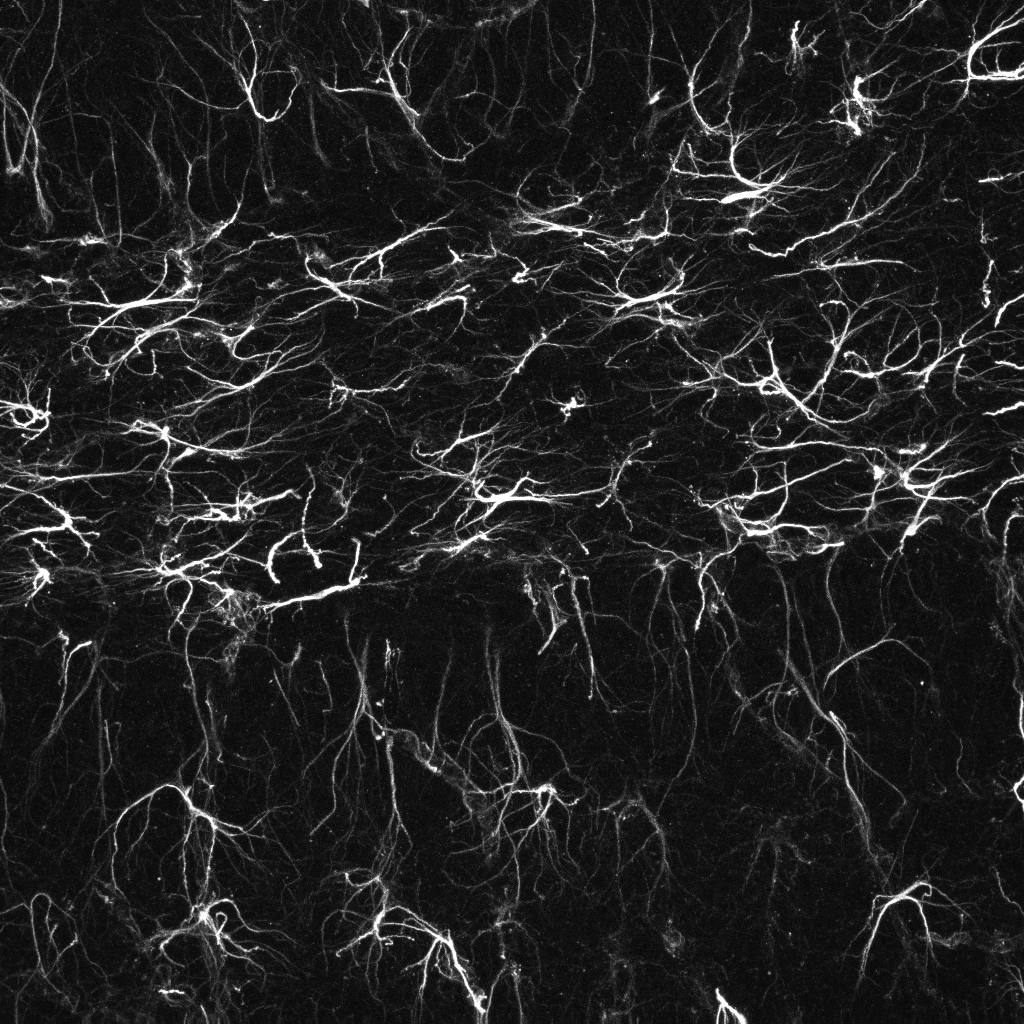

Supplement: Supplementary file 6 — Source Data for Figure 2 [file EMBJ-42-e110384-s006.zip › Figure 2 Source Data/G/Yap1 cKO.tif]
