# Supplementary material for: The transcriptional co‐activator Yap1 promotes adult hippocampal neural stem cell activation
Source: EMBO J. 2023 Apr 21;42(11):e110384. doi: 10.15252/embj.2021110384 (PMC10233373; doi:10.15252/embj.2021110384)
Supplement: Supplementary file 4 — PDF+ [file EMBJ-42-e110384-s003.pdf]

Article

SOURCE DATA | TRANSPARENT PROCESS | OPEN ACCESS

THE EMBO JOURNAL

The transcriptional co-activator Yap1 promotes adult hippocampal neural stem cell activation

Wenqiang Fan[1,†], Jerónimo Jurado-Arjona[1,2,*], Gregorio Alanis-Lobato[3,‡], Sophie Péron[1,2], Christian Berger[4], Miguel A Andrade-Navarro[3], Sven Falk[5] & Benedikt Berninger[1,2,6,7,8,**]

Abstract

Most adult hippocampal neural stem cells (NSCs) remain quiescent, with only a minor portion undergoing active proliferation and neurogenesis. The molecular mechanisms that trigger the transition from quiescence to activation are still poorly understood. Here, we found the activity of the transcriptional co-activator Yap1 to be enriched in active NSCs. Genetic deletion of Yap1 led to a significant reduction in the relative proportion of active NSCs, supporting a physiological role of Yap1 in regulating the transition from quiescence to activation. Overexpression of wild-type Yap1 in adult NSCs did not induce NSC activation, suggesting tight upstream control mechanisms, but overexpression of a gain-of-function mutant (Yap1-5SA) elicited cell cycle entry in NSCs and hilar astrocytes. Consistent with a role of Yap1 in NSC activation, single cell RNA sequencing revealed a partial induction of an activated NSC gene expression program. Furthermore, Yap1-5SA expression also induced expression of Taz and other key components of the Yap/Taz regulon that were previously identified in glioblastoma stem cell-like cells. Consequently, dysregulated Yap1 activity led to repression of hippocampal neurogenesis, aberrant cell differentiation, and partial acquisition of a glioblastoma stem cell-like signature.

Keywords hippocampus; neurogenesis; quiescence; radial glia; single cell RNA sequencing
Subject Categories Neuroscience; Stem Cells & Regenerative Medicine
DOI 10.15252/embj.2021110384 | Received 11 January 2022 | Revised 20 March 2023 | Accepted 27 March 2023 | Published online 21 April 2023
The EMBO Journal (2023) 42: e110384

Introduction

During embryonic development, neural stem cells (NSCs) migrate from the dentate epithelium to settle in the dentate gyrus anlage of the hippocampus. Some of these will then eventually give rise to NSCs in the adult dentate gyrus (Berg *et al*, 2019). Located in the subgranular zone (SGZ), many of these Sox2-positive NSCs are characterized by a glial fibrillary acidic protein (GFAP)-positive radial process that extends across the granule cell layer towards the molecular layer, lending them a radial glia-like appearance (Seri *et al*, 2001; Suh *et al*, 2007). Alongside these radial glia-like NSCs, the SGZ harbors also Sox2-positive nonradial neural progenitor cells shown to be derived from dividing radial glia-like cells and themselves capable of undergoing either symmetric neurogenic divisions or asymmetric divisions, yielding one renewed nonradial cell and one neuronal daughter cell (Pilz *et al*, 2018). A key process in the establishment of the adult NSC pool is the progressive entry of NSCs into a quiescent state in which many will persist before eventually becoming re-activated during adulthood and re-entering cell cycle (Berg *et al*, 2019). The regulation of both transitions, that is, the transition from actively dividing to quiescent and the converse, the *de novo* activation is one of the key mysteries of adult NSC biology (Urbán *et al*, 2019). Quiescence is defined as “reversible cell cycle arrest” and is believed to protect stem cells against damage and premature exhaustion of the stem cell pool (Urbán *et al*, 2019). Intriguingly, the maintenance of quiescence is dynamically regulated in that, during aging, the rate of activation decreases with more NSCs persisting in a quiescent state (Dulken *et al*, 2019; Kalamakis *et al*, 2019; Harris *et al*, 2021; Ibrayeva *et al*, 2021). Several signaling pathways have been implicated in the bidirectional traffic between quiescence and activation, including BMP (Mira *et al*, 2010), Notch (Kawai *et al*, 2017; Engler *et al*, 2018; Zhang *et al*, 2019; Harada *et al*, 2021), VEGFR3 (Han *et al*, 2015), and Wnt signaling (Lie

1 Institute of Physiological Chemistry, University Medical Center of the Johannes Gutenberg University Mainz, Mainz, Germany
2 Centre for Developmental Neurobiology, Institute of Psychiatry, Psychology & Neuroscience, King’s College London, London, UK
3 Faculty of Biology, Johannes Gutenberg University Mainz, Mainz, Germany
4 Institute of Genetics, Johannes Gutenberg University Mainz, Mainz, Germany
5 Institute of Biochemistry, Friedrich-Alexander-Universität Nürnberg-Erlangen, Erlangen, Germany
6 MRC Centre for Neurodevelopmental Disorders, Institute of Psychiatry, Psychology & Neuroscience, King’s College London, London, UK
7 The Francis Crick Institute, London, UK
8 Focus Program Translational Neuroscience, Johannes Gutenberg University Mainz, Mainz, Germany
*Corresponding author. Tel: +44 (0) 207 848 6532; E-mail: benedikt.berninger@kcl.ac.uk
**Corresponding author. Tel: +44 (0) 207 848 6548; E-mail: jjurado@kcl.ac.uk
†Present address: Neuroscience Therapeutic Area, New Medicines, UCB Biopharma SPRL, Braine-l’Alleud, Belgium
‡Present address: Global Computational Biology and Data Sciences, Boehringer Ingelheim Pharma GmbH & Co. KG, Biberach an der Riss, Germany

© 2023 The Authors. Published under the terms of the CC BY 4.0 license.

et al, 2005; Wexler et al, 2009) but also include circuit-specific mechanisms (Song et al, 2012). At a transcriptional level, previous work has shown that in the adult hippocampus expression of Ascl1 is a prerequisite for activation of quiescent NSCs (Andersen et al, 2014), while degradation of Ascl1 protein is an important step in controlling return to quiescence (Urbán et al, 2016). However, the full spectrum of transcriptional mechanisms underpinning NSC activation is still incompletely understood.

Accruing evidence points to the importance of the Hippo signaling pathway in regulating stem cell renewal and reactivation during regenerative processes in various tissues (Mo et al, 2014; Moya & Halder, 2019). The Hippo pathway is an evolutionarily conserved kinase cascade that regulates the activity of its effector proteins, the transcriptional activators Yap1 (Yes-associated protein 1) and Taz, through phosphorylation, thereby causing their cytoplasmic retention and degradation (Yu & Guan, 2013). Several mechanisms converge on this kinase cascade, including mechanical stimuli, cell polarity, and other signaling pathways (Yu & Guan, 2013).

Recent work has implicated the Hippo effector protein Yorkie (Yki) in the control of activation of quiescent NSCs in the brain of *Drosophila* larvae (Ding et al, 2016; Gil-Ranedo et al, 2019). For instance, loss of Yki has been shown to result in the failure of larval NSCs to grow in response to hormone stimulation and to re-enter cell cycle (Ding et al, 2016). The Hippo pathway and the Yki orthologue Yap1 have been shown to be highly active in the developing mammalian forebrain and to regulate radial glial cell proliferation (Cappello et al, 2013; Lavado et al, 2018, 2021; Kostic et al, 2019; Han et al, 2020; Mukhtar et al, 2020; Najas et al, 2020), but its role in the functional state of NSCs in adult neurogenic niches has not been addressed so far.

Thus, we wondered whether Yap1 activation is an evolutionarily conserved mechanism in the regulation of NSC activity. Towards this, here we have re-analyzed published single cell RNA-sequencing data (Hochgerner et al, 2018), which revealed that Yap1 is highly enriched in activated NSCs of the adult hippocampus. This has prompted us to perform loss- and gain-of-function studies in the adult hippocampus, which provide evidence for an important role of this transcriptional co-activator in the regulation of adult hippocampal NSC activity.

Results

Evidence for Yap1 activity in active neural stem cells of the adult hippocampus

To obtain first evidence for ongoing *Yap1* activity in the lineage of adult hippocampal neural stem cells (NSCs), we first re-analyzed a published dataset of single cell transcriptomes comprising NSCs and their early-stage progeny isolated from the dentate gyrus of the adult hippocampus (Data ref: Hochgerner et al, 2018). This identified a cell population enriched in a reference signature of active NSCs (Shin et al, 2015), originally classified as neuronal intermediate progenitor cells (nIPCs) (Appendix Fig S1). We next assessed which of these cell populations, if any, exhibited expression of a reference signature of *Yap1* activity (Cordenonsi et al, 2011). This revealed a specific enrichment of *Yap1* activity signature in the same cell population enriched for the active NSC signature (Appendix Fig S1). This analysis, thus, suggests that activation of NSC may involve Yap1-mediated transcription.

Based on this analysis, we next examined whether we could detect Yap1 protein expression within the NSC lineage (Fig 1). We found Yap1 specifically expressed in Sox2-positive cells within the subgranular zone (SGZ) (Fig 1A) and hilar astrocytes (identified by GFP expression driven from human GFAP [hGFAP] gene regulatory elements and hilar localization; Fig 1B) but virtually absent in doublecortin (DCX)-positive young neurons (Fig 1C) or Olig2-positive cells (i.e., comprising cells of oligodendroglial lineage; Fig 1D). While in hilar astrocytes Yap1 immunoreactivity appeared to be largely cytoplasmic, within the SGZ, some of the Yap1 immunoreactivity co-localized with Sox2 expression, demarcating the nuclei of these Sox2-positive cells (Fig 1A, inset). These data suggest that Sox2-positive SGZ cells (i.e., NSCs and intermediate progenitor cells) are enriched in Yap1 expression and that Yap1 may have undergone nuclear translocation in a subpopulation of these cells. To further characterize Yap1 expression dynamics during the lineage progression from quiescent NSCs to early neuronally-restricted progenitor cells, we assessed nuclear Yap1 co-localization with the quiescent NSC marker Hopx, the activated NSC marker Ascl1 and the neuronal progenitor marker Tbr2, respectively (Fig 1E). This indicated a trend towards increased nuclear Yap1 expression following NSC activation and a significant drop in nuclear Yap1 following neuronal commitment (Fig 1E and F).

In order to confirm that NSC activation is associated with nuclear enrichment of Yap1, we took advantage of primary adult hippocampal NSC cultures (Babu et al, 2011; Fig EV1A), which have been successfully used to study the transition between NSC states of activation and quiescence (Martynoga et al, 2013; Fig EV1B–D). Immunofluorescence analysis for Yap1 location revealed a clear enrichment in nuclear Yap1 in active versus quiescent NSCs (Fig EV1E and F). Conversely, when previously activated adult hippocampal NSC cultures were treated with BMP4 for a brief period to induce the transition towards quiescence, we observed nuclear Yap1 enrichment in those cells still in cell cycle (i.e., Ki67-positive), while Ki67-negative cells exhibited a significant reduction in nuclear Yap1 (Fig EV1G and H). These data support the notion that NSC activation is accompanied by the transfer of Yap1 from the cytoplasm to the nucleus, while the reverse process takes place during the return to quiescence.

Long-term *Yap1* loss-of-function compromises NSC activation

To study the role of *Yap1* in regulating the NSC transition from quiescence to activation, we next analyzed the impact of *Yap1* loss-of-function on the ratio of quiescent and active NSCs. Towards this, we deleted *Yap1* specifically in NSC (and astrocytes) and their lineage by crossing *Yap1* conditional knockout mice (Yap1fl/fl) (Zhang et al, 2010) with Glast-CreERT2 and CAG-CAT-EGFP mice (Mori et al, 2006; Nakamura et al, 2006) followed by treatment with tamoxifen (Fig 2A). Immunofluorescence for Yap1 revealed a reduction in expression by day 7 following induction of recombination, indicating successful *Yap1* deletion (Fig 2B and C). To assess the proportion of actively dividing cells among NSCs 7 days after induction of recombination, we first determined the number of recombination reporter-positive (GFP-positive) cells among radial glia-like cells (RGLs, i.e., the major NSC population, identified by their localization in the SGZ, GFAP immunoreactivity, and the presence of a radially-oriented process) and then quantified those RGLs that were engaged in cell cycle (minichromosome maintenance complex component-2, Mcm2-positive

 © 2023 The Authors

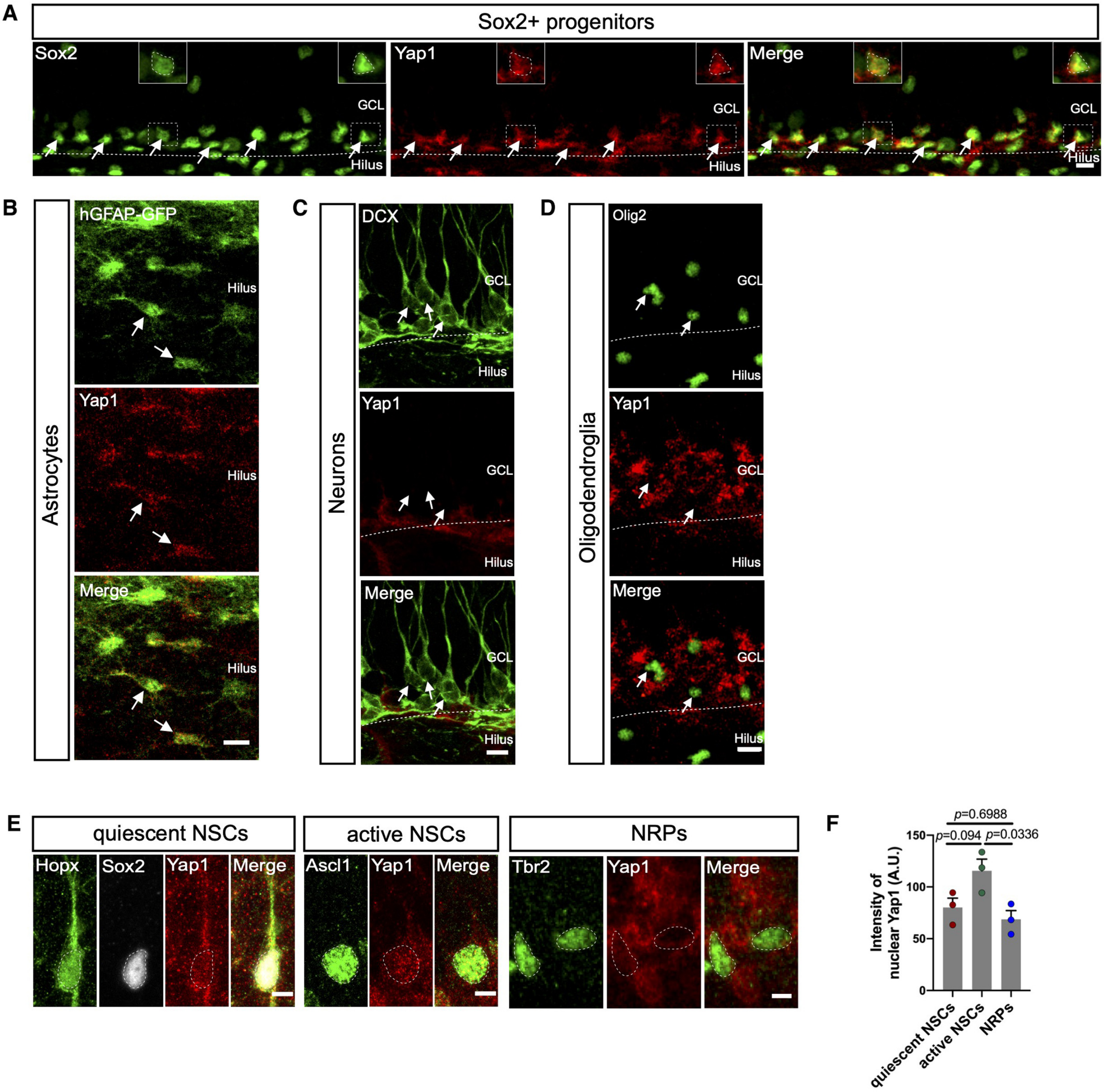

Figure 1. Expression pattern of Yap1 in the DG of the adult hippocampus.

A Representative images of immunostainings of adult mouse brain coronal sections showing that Yap1 (red) is expressed in Sox2-positive (green) neural progenitors of SGZ in the DG. Arrows indicate double-positive cells.

B Immunofluorescence for hGFAP-GFP and Yap1. Arrows indicate GFP-positive astrocytes. Note the presence of Yap1 protein in hilar astrocytes.

C Immunofluorescence for DCX and Yap1. Arrows indicate DCX-positive immature neurons, which do not express Yap1.

D Immunofluorescence for Olig2 and Yap1. Arrows indicate Olig2-positive oligodendroglial cells. Note the absence of Yap1 protein in oligodendroglial cells.

E Representative images of expression of Yap1 in quiescent NSCs (Sox2 and Hopx-positive cells), active NSCs (Ascl1-positive cells), and Tbr2-positive cells neuronally-restricted progenitor cells (NRPs).

F Quantification of Yap1 intensity in (B). n = 23 quiescent NSCs from three mice (biological replicates), n = 14 active NSCs from 3 mice, and n = 30 NRPs from three mice (biological replicates).

Data information: Data are represented as mean ± SEM. One-way ANOVA. GCL, granule cell layer. Scale bars: 10 μm for (A and D); 5 μm for (B).
Source data are available online for this figure.

© 2023 The Authors

cells; Fig 2D). Quantitative, double-blind analysis revealed that the proportion of active versus quiescent (Mcm2-positive versus Mcm2-negative) RGLs had not changed on day 7 following the deletion of *Yap1* as compared to controls (Fig 2E and F). Likewise, when we performed the same double-blind analysis 30 days after inducing the deletion of *Yap1*, we did not observe significant changes in active versus quiescent RGLs (Fig EV2A and B). By contrast, on day 60 following the deletion of *Yap1* (Fig EV2G), we noted a significant drop in the number of active RGLs as compared to controls (Fig 2G–I). This set of experiments indicates that loss of *Yap1* does not immediately impact on the levels of NSC transition from quiescence to activation but that long-term *Yap1* loss-of-function results in decreased NSC activation, evidencing an important physiological role of *Yap1* in this process. One possible explanation for this delayed manifestation of *Yap1* loss-of-function maybe dependence on other mechanisms for NSC activation. Given the fact that nuclear expression of Yap1 was even more conspicuous in hippocampal NSCs treated with EGF and FGF2 *in vitro*, we tested the impact of *Yap1* deletion on NSC proliferation in culture by assessing the incorporation of the thymidine analog EdU (Fig EV2H). This revealed a drastic drop in DNA synthesis following *Yap1* loss-of-function (Fig EV2I). These *in vitro* data qualitatively support our observations *in vivo*, while at the same time suggesting quantitative differences in the dependence of NSC activation on *Yap1* function.

Interestingly, the deletion of *Yap1* did not negatively impact lineage progression towards neurogenesis *in vivo* as *Yap1*-deleted NSCs gave rise to DCX-positive neurons to a similar extent as control NSCs when assessed at either 30 or 60 days following tamoxifen administration (Fig EV2C–F).

Active Yap1 promotes cell cycle entry of quiescent hippocampal NSCs

We next investigated the effect of *Yap1* gain-of-function on the activation state of adult hippocampal NSCs. Towards this, we targeted adult NSCs (as well as hilar astrocytes) using lentiviruses driving transgene expression under the control of hGFAP regulatory elements (LV-hGFAP-IRES-EGFP, LV-hGFAP-Yap1WT-IRES-EGFP, LV-hGFAP-Yap1-5SA-IRES-EGFP; Appendix Fig S2). Interestingly, 7 days following lentivirus-mediated expression of the wild-type form of *Yap1* (Yap1 WT) in adult hippocampal SGZ, no significant change in the number of proliferative Sox2-positive progenitors (i.e., lentivirus-transduced Sox2-positive cells in the SGZ) was observed (Fig 3A–C). By contrast, lentivirus-mediated expression of a mutant form of *Yap1*, encoding a protein in which the serines of the five HXRXXS Lats motifs had been replaced by alanine (Yap1-5SA), rendering the protein insensitive to phosphorylation-dependent inhibition by Lats and thereby constitutively active (Zhao *et al*, 2007), caused a dramatic rise in the number of lentivirus-transduced Ki67-positive/Sox2-positive cells (Fig 3B and C). Finally, we also observed an overall increase in the density of Sox2-positive cells in the SGZ (Fig 3B and D). These data show that active Yap1 is sufficient to drive quiescent NSCs towards cell cycle entry. It also supports the notion that Yap1 activity is under strict control as a forced expression of Yap1 WT fails to induce NSC activation, and only a regulation-deficient form of Yap1 overcomes this control. In line with the Yap1-mediated activation of hippocampal NSC *in vivo*, we found that lentivirus-mediated expression of Yap1-5SA promoted dramatic cell cycle entry in primary cultures of hippocampal NSCs kept in quiescence conditions as assessed by phosphohistone-H3 staining and EdU incorporation (Fig EV3A–D).

Finally, in these injections, some astrocytes in the hilus of the dentate gyrus were targeted by the lentiviruses encoding GFP only or Yap1-5SA and GFP. While we did not observe any Ki67-positive astrocytes following control virus injection, many astrocytes were Ki67-positive following the expression of Yap1-5SA. This indicates that Yap1-5SA also stimulates cell cycle re-entry in postmitotic astrocytes (Fig EV3E and F).

Constitutively active Yap1 induces an aberrant active NSC-like signature in adult hippocampal NSCs and/or astrocytes

Given our observation that forced expression of Yap1-5SA appears to activate quiescent NSCs, we finally addressed how this molecular perturbation alters gene expression using single cell RNA-sequencing. Yap1-5SA was targeted to adult hippocampal NSCs and hilar astrocytes by injection of lentiviruses encoding Yap1-5SA and EGFP or EGFP alone for control, under hGFAP regulatory elements, into the SGZ of the adult dentate gyrus. We performed fluorescence-activated cell sorting (FACS) to isolate EGFP-positive cells on day 3 and 7 following lentivirus transduction (Appendix Fig S3). We selected isolation on day 3 as this was the earliest time point when we could detect reliable EGFP expression, while day 7 corresponds to the time point when we had previously observed massive activation of quiescent NSCs (Fig 3). Single cell transcriptomes were measured following library preparation using Smart-seq2 methodology (Fig 4A) (Picelli *et al*, 2014). This yielded 121 (day-3) and 258 (day-7) control and 273 (day-3) and 320 (day-7) Yap1-5SA-expressing single cell transcriptomes. Using Scanpy (Wolf *et al*, 2018) we identified 13 distinct clusters (Appendix Fig S4A) and employed force-directed graph embedding for visualization. Clusters expressing astrocyte, quiescent NSC, neuron, oligodendrocyte precursor cell (OPC), oligodendrocyte, and microglia markers consisted exclusively of control cells (Fig EV4A–H). Conspicuously, none of these clusters contained cells expressing Yap1-5SA, suggesting that this perturbation exerts a drastic effect on the targeted cells forcing them to alter their cell state within a 3-day time window. Among the remaining clusters, several contained both control and Yap1-5SA-expressing cells, while a final set of clusters consisted of Yap1-5SA cells only. Consistent with Yap1-5SA being a transcriptional co-activator (Zhao *et al*, 2007), clusters containing Yap1-5SA-expressing cells exhibited an increased number of transcripts compared with control cell clusters (Fig EV4B). To further reveal the identity of Yap1-5SA-expressing cells and their lineage relationship to quiescent NSC and astrocytes, we performed re-clustering after removal of microglia, OPCs, oligodendrocytes, as well as a cluster enriched in cell death-related genes (Mdm2, Trp53), while the cluster identified with neurons was retained as the main natural progeny of the adult hippocampal NSC lineage (Fig 4B–F). This re-analysis yielded 12 clusters comprising astrocytes, quiescent NSCs, neurons, none of which contained Yap1-5SA cells, and nine clusters entirely consisting of Yap1-5SA-expressing cells (Fig 4B and E; Appendix Fig S4). To obtain insights into the lineage dynamics between the different clusters, we employed RNA velocity with scVelo (La Manno *et al*, 2018; Bergen *et al*, 2020), supporting the notion that the overall lineage progression vector originated from

 © 2023 The Authors

the cluster comprising the NSC population (Fig 4C and E). Indeed, pseudotime analysis identified NSCs as a source for all other cells, with neurons representing the most distant differentiated fate (Fig 4C and E), consistent with the natural lineage progression of adult hippocampal neurogenesis. As expected, control-transduced cells showed low levels of gene expression related to cell cycle (*cell cycle signature* (Hao *et al*, 2021), Fig 4D) or Yap1 activity (*Yap1 signature* (Cordenonsi *et al*, 2011), Fig 4D). Moreover, only few cells were enriched in gene expression related to an activated NSC state (*activated NSC signature*; Shin *et al*, 2015), consistent with the fact that most adult hippocampal NSCs are quiescent (*quiescent NSC signature* (Shin *et al*, 2015), Fig 4D and Appendix Fig S4). By contrast, following Yap1 gain-of-function, we observed an enrichment in Yap1 signature across all Yap1-5SA-expressing clusters (Fig 4D).

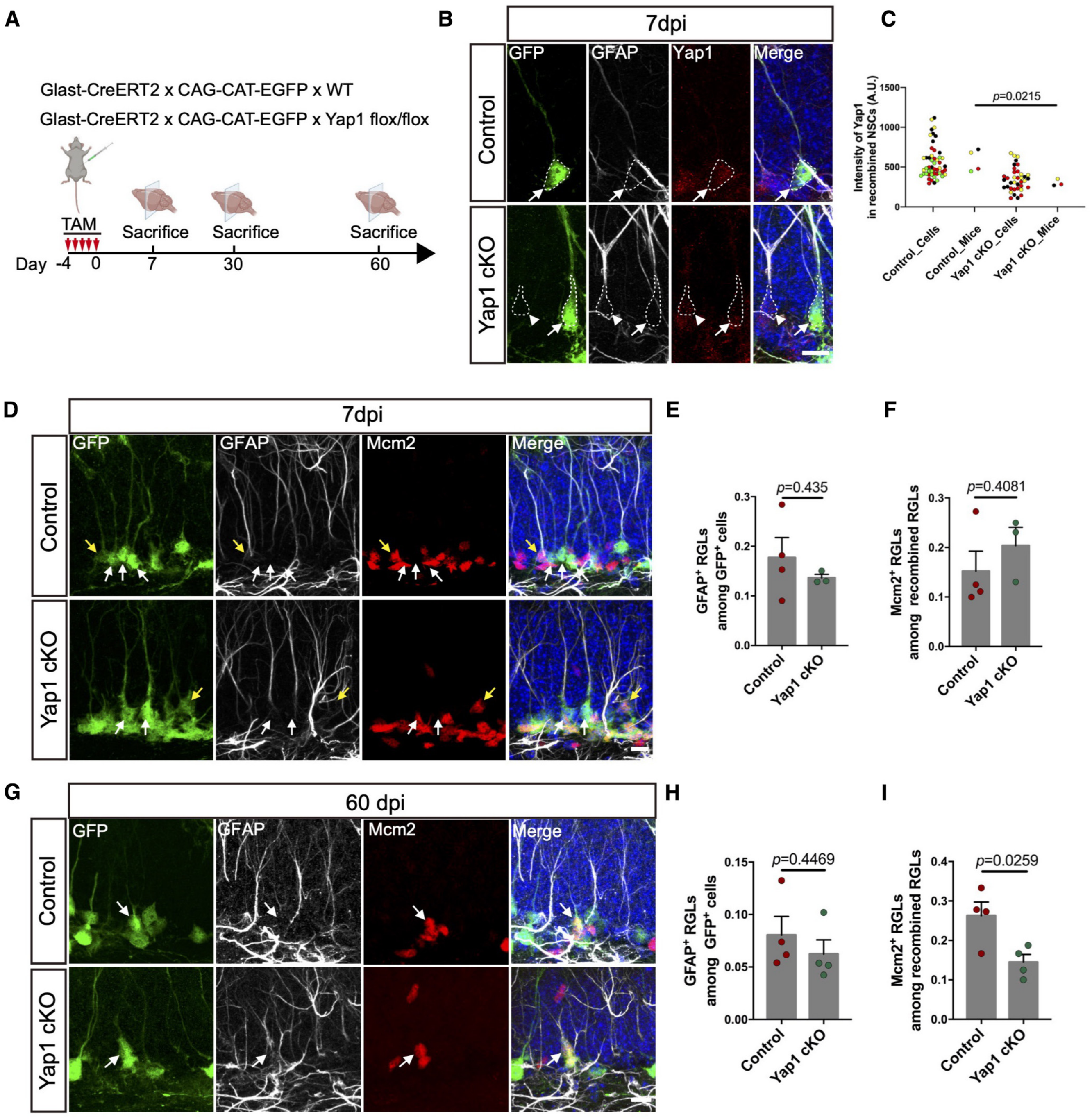

Figure 2.

Figure 2. Yap1 regulates the balance between quiescent and active NSCs.

A Schematic diagram of the experimental design. Adult (8-week-old) Yap1 cKO mice or Control mice were injected with Tamoxifen for 5 consecutive days and sacrificed 7, 30, or 60 days later for immunohistological analysis.

B Immunofluorescence for GFP (recombination reporter), GFAP, and Yap1 in control and Yap1 cKO RGLs at 7 days after tamoxifen administration. Arrowhead indicates nonrecombined RGL, and arrow indicates recombined RGL.

C Quantification of Yap1 intensity in (B). Yap1 levels are significantly decreased in Yap1 cKO RGLs at 7dpi. $n = 4$ (50 cells, four mice, biological replicates) in the control group, $n = 3$ (37 cells, three mice, biological replicates) in the Yap1 cKO group.

D Immunofluorescence for GFP, GFAP, and Mcm2 in control and Yap1 cKO RGLs at 7 days after tamoxifen administration. Yellow arrow indicates proliferating adult RGL, and white arrows indicate quiescent adult RGLs.

E, F Quantification of the data in (D). Loss of Yap1 does not induce any changes neither in the number of proliferative RGLs nor the overall number of RGLs. $n = 4$ mice (biological replicates) for control group, $n = 3$ mice (biological replicates) for Yap1 cKO group.

G Immunofluorescence for GFP, GFAP, and Mcm2 in control and Yap1 cKO RGLs at 60 days after tamoxifen administration. Arrows indicate Mcm2-positive, proliferating RGLs.

H, I Quantification of the data in (G). Loss of Yap1 results in the decrease in proliferative RGLs, while the overall number of RGLs does not change. $n = 4$ mice (biological replicates) in control, $n = 4$ mice (biological replicates) in Yap1 cKO.

Data information: Data are represented as mean ± SEM. Unpaired Student's *t*-test. Scale bars: 10 μm. dpi, days postinjection.
Source data are available online for this figure.

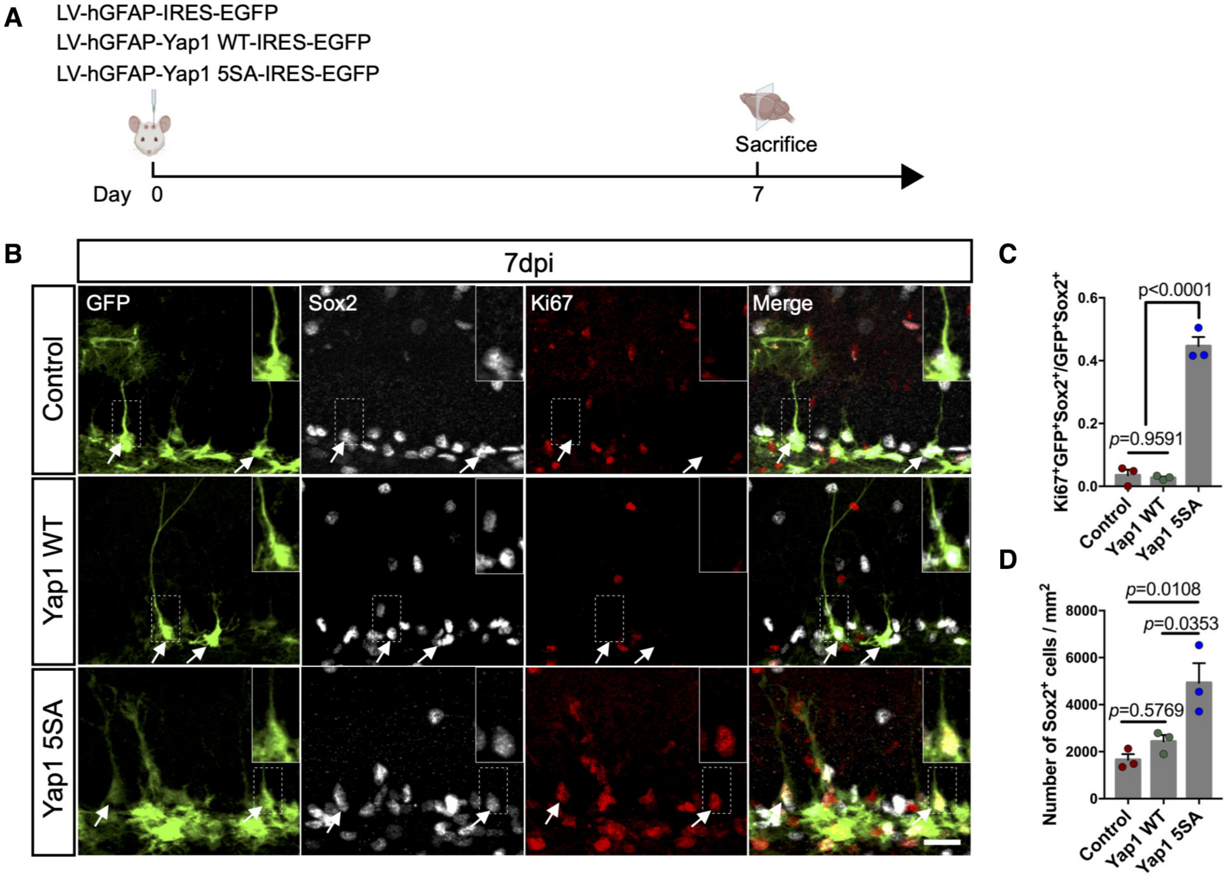

Figure 3. Yap1 gain-of-function induces adult hippocampal NSC proliferation *in vivo*.

A Schematic diagram of experimental design. P60 mice were analyzed at 7 days after lentivirus injection of control, wild-type Yap1 and Yap1-5SA.

B Immunofluorescence for GFP, Sox2 (to identify NSCs, arrows), and Ki67 (to identify proliferative cells) in control conditions or overexpression of wild-type Yap1 or Yap1-5SA 7 days after lentivirus injection. Insets show a magnified view of the areas outlined by the dashed lines.

C, D Quantification of proliferative NSCs. Yap1-5SA induces the proliferation of adult NSCs and increases the number of Sox2-positive cells. $n = 3$ mice (biological replicates) for each group.

Data information: Data are represented as mean ± SEM. One-way ANOVA. Scale bars: 50 μm.
Source data are available online for this figure.

 © 2023 The Authors

Yap1-5SA-expressing clusters transcriptionally closer to control-transduced NSCs exhibited elevated cell cycle signature (Figs 4D and EV4J). Accordingly, these clusters (2,4-7) downregulated genes enriched in quiescent NSCs (Fig 4D), while up-regulating an activated NSC signature (Fig 4D; Appendix Fig S4). These data are thus consistent with the notion that *Yap1* gain-of-function can elicit activation of quiescent NSCs and cell cycle entry, as observed in our *in vivo* and *in vitro* experiments (Figs 3 and EV2). One intriguing observation was the absence of significant Ascl1 expression in Yap1-5SA-expressing cells, in sharp contrast to control NSCs, consistent with the fact that the former cells failed to follow a trajectory leading to adult neurogenesis. While Yap1-5SA-expressing did not contribute to neuronal fate (Fig EV4A), we observed at least two additional RNA velocity vector endpoints (clusters 9 and 11) comprising exclusively Yap1-5SA-expressing cells and characterized by reduced cell cycle and activated NSC signatures (Figs 4B and D, and EV4A). Intriguingly, these clusters were typified by high levels of Scg2 (cluster 11) and Pmp22 (cluster 9) expression, respectively (Fig 4E). However, these cells also expressed an arsenal of non-neural-specific genes, suggestive of aberrant differentiation (Appendix Fig S4A). Also, while less segregated in pseudotime, Yap1-5SA-expressing cluster 1 also showed a reduced cell cycle signature and exhibited a prominent expression of mesenchymal cell-type-related genes (e.g., Col1a1, Fig 4D and E and Appendix Fig S4A). Given the strong implication of Yap1 in brain tumor initiation, we finally scored control and Yap1-5SA-expressing cells for their expression of glioblastoma stem cell-related genes (*glioblastoma stem cell-like signature*; Castellan *et al*, 2021), such as the Yap1 paralogue transcriptional regulator Taz and some of their primary downstream transcriptional regulators (Fig 4F). While Yap1-5SA-expressing cells were overall enriched in glioblastoma stem cell-like signature across clusters, clusters 9 and 11 showed lower enrichment consistent with their partial, albeit aberrant differentiation. Finally, we noted that most Yap1-5SA-expressing clusters comprised cells isolated on days 3 or 7 but enrichment of day-7 cells in clusters 2 and 1 (Fig 4B). This may suggest that progression towards a more mesenchymal phenotype may involve prolonged Yap1 activity. In sum, our scRNAseq data indicate that Yap1 gain-of-function in adult hippocampal NSC and astrocytes can induce molecular hallmarks of an activated NSC. However, prolonged Yap1 activity disrupts the physiological molecular trajectory towards neurogenesis and instead induces a gene expression signature akin to glioblastoma stem cells and can lead to aberrant differentiation.

To address the long-term consequences of *Yap1* gain-of-function, we finally analyzed dentate gyri of animals 30 days after lentivirus-mediated control *GFP*, wild-type *Yap1*, or *Yap1-5SA* expression (Fig EV5A). While even after 30 days, wild-type *Yap1* did not cause any visible alterations in the cytoarchitecture of the dentate gyrus, the latter was massively disrupted following *Yap1-5SA* gain-of-function (Fig EV5B), accompanied by a drastic increase in the number of Sox2-positive cells (Fig EV5B and C). Moreover, whereas control reporter- or wild-type *Yap1*-expressing cells were capable of undergoing neurogenesis (as assessed by morphology and location in the granule cell layer), *Yap1-5SA*-expressing cells maintained Sox2 expression (Fig EV5C) and failed to acquire NeuN expression (Fig EV5D). Likewise, *Yap1-5SA*-expressing cells remained negative for the astrocytic protein S100ß indicating that these cells also did not differentiate into astrocytes (Fig EV5E).

Discussion

In this study, we examined the hypothesis that the transcriptional co-activator Yap1 plays a role in the transition of adult hippocampal NSCs from quiescence to activation. Consistent with this hypothesis, we find that Yap1 expression is enriched in activated NSCs and YAP1 gain-of-function can trigger cell cycle entry in quiescent NSCs. Conversely, Yap1 gene deletion in adult hippocampal NSCs results in a reduction in the ratio of activated vs. quiescent NSCs. Finally, prolonged Yap1 activity in adult hippocampal NSCs disrupts physiological neurogenesis promoting aberrant cell differentiation and partial acquisition of a glioblastoma stem cell-like signature.

Our study set out from the re-analysis of published scRNAseq data (Data ref: Hochgerner *et al*, 2018) showing that Yap1 activity is highly enriched in activated NSCs, suggesting that Yap1 may play an important role as a regulator of the transition of adult hippocampal NSCs from a quiescent to an activated state. A key step in the regulation of Yap1 activity consists of its translocation from the cytoplasm to the nucleus. Indeed, we did not only find that Yap1 is selectively expressed in adult hippocampal NSCs *in vivo*, but a hippocampal NSC culture allowed us to reveal that activation of quiescent NSCs is accompanied by a marked nuclear translocation of Yap1 protein. Intriguingly, *in vivo* overexpression of wild-type Yap1 did not elicit overt NSC activation. These data indicate that in our *in vivo* experiments, regulatory mechanisms upstream of Yap1 were not overwhelmed by the elevated Yap1 protein levels, suggesting tight control over wild-type Yap1 activity in adult hippocampal NSCs under normal conditions. Interestingly, when quiescent NSCs in culture are experimentally activated by removal of BMP4 and treatment with EGF/FGF2 (Martynoga *et al*, 2013), NSCs are relieved from this tight control, as illustrated by the drastic increase in nuclear Yap1 protein. In contrast to wild-type Yap1, overexpression of a Yap1 mutant defective for phosphorylation-mediated inhibition (Yap1-5SA; Zhao *et al*, 2007) induced cell cycle entry in adult hippocampal NSCs. This clearly shows the potency of Yap1 in promoting NSC activation once relieved from inhibitory control. Likewise, we found that hilar astrocytes commence to proliferate in response to Yap1-5SA overexpression.

In the present study, we did not address the upstream mechanisms responsible for the effective control of Yap1 activity in NSCs or astrocytes. In the mouse retina, Müller glia have been shown to exit quiescence and commence proliferation upon deletion of Lats1 and 2, that is, key components of the canonical Hippo pathway, indicating that Hippo signaling exerts inhibitory control under physiological conditions (Hamon *et al*, 2019; Rueda *et al*, 2019). While it is likely that similar mechanisms also operate in the adult NSC niche, it remains to be shown what molecular signals converge onto the Hippo pathway to maintain Yap1 activity at bay. Conversely, it is likely that multiple extracellular and intracellular signaling mechanisms converge to activate Yap1 in adult NSCs. Yap1 and Taz have been shown to act as signal transducers of tyrosine kinase receptor pathways such as the VEGF-VEGFR2 pathway in angiogenesis (Wang *et al*, 2017), and it is conceivable that related tyrosine kinase pathways exert their effects on adult NSCs via Yap1 (Han *et al*, 2015).

Given the apparent tight control of Yap1 in adult hippocampal NSCs, we were somewhat surprised to see the initially only very

© 2023 The Authors

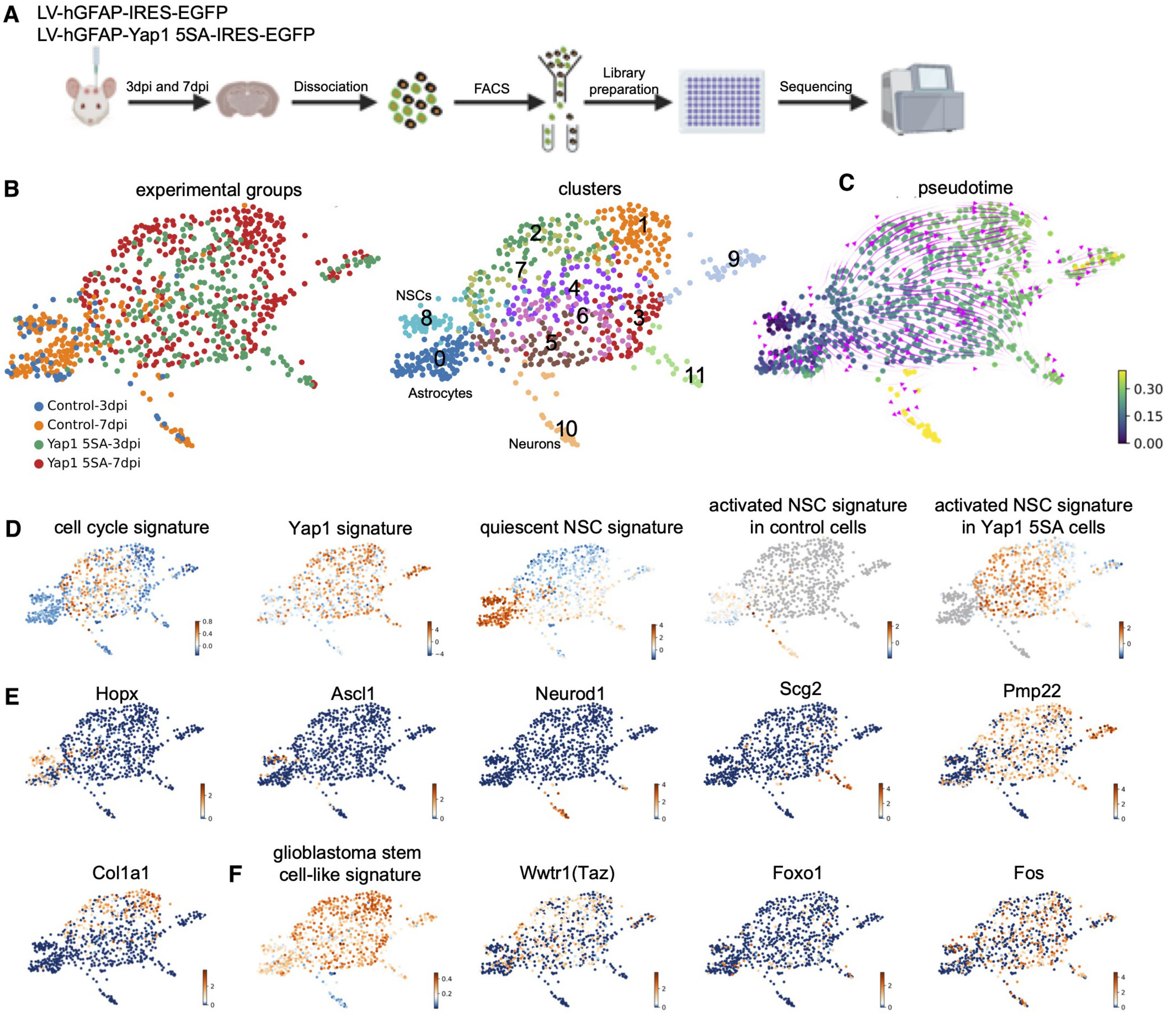

Figure 4. Overexpression of active Yap1 induces an aberrant active NSC-like signature.

A Schematic diagram of experimental design of single cell RNA sequencing at 3 and 7 days after viral injection.

B Clustering after force-directed graph embedding and removal of cells with microglia, OPCs, oligodendrocytes, or cell death signatures.

C Diffusion pseudotime and RNA velocity analysis indicating cell fate trajectories.

D Force-directed graph plots showing signatures of cell cycle-related, Yap1 activity-related, quiescent NSC-related, and activated NSC-related gene expression.

E Force-directed graph showing expression of Hopx, Ascl1, Neurod1, Scg2 and Pmp22, and Col1a1.

F Force-directed graph displaying glioblastoma stem cell-like signature and expression of Yap/Taz hub genes Wwtr1 (Taz), Foxo1, and Fos.

subtle effect of Yap1 deletion (i.e., at seven or 30 days after deletion). A significant change in the ratio of activated vs. quiescent NSCs was only detected after 60 days postdeletion. One reason for this delayed response may consist of the temporary compensation by the Yap1 paralogue Taz (Plouffe *et al*, 2018). In fact, while the deletion of Yap1 did not significantly alter the proliferation of cortical radial glia (Park *et al*, 2016; Huang *et al*, 2016a), Lavado *et al* (2021) observed a significant reduction in radial glia proliferation and neurogenesis following combined Yap1 and Taz deletion. A second reason may be related to the fact that even with early aging, the frequency of quiescent NSCs to become activated decreases significantly (Kalamakis *et al*, 2019; Harris *et al*, 2021; Ibrayeva *et al*, 2021), possibly due to the downregulation of activation-promoting and/or upregulation of quiescence-retaining mechanisms (Harris *et al*, 2021). Such changes could render the activation of quiescent adult hippocampal NSCs increasingly dependent on Yap1 activity and thereby enhance the consequences of Yap1 deletion compared with earlier stages. Finally, Yap1 loss-of-

 © 2023 The Authors

function may cause a gradual but indirect decline in NSC activation by promoting an inflammatory response in local astrocytes or NSCs themselves (Huang *et al*, 2016b), which in turn could promote a progressive increase in NSC quiescence as shown during aging (Kalamakis *et al*, 2019). In any case, the physiological requirement of Yap1 in adult NSC activation observed in our study is highly reminiscent of the impaired reactivation of Müller glia in the injured adult mouse retina following Yap1 gene deletion (Hamon *et al*, 2019, Rueda *et al*, 2019). Also, given its proliferation-promoting effect on hilar astrocytes, it will be interesting to decipher the physiological contribution of Yap1 to the, albeit limited, proliferative activity of parenchymal astrocytes during reactive gliosis. Intriguingly, in the early postnatal cerebral cortex Yap1 loss-of-function was suggested to reduce astrocyte proliferation while at the same time promoting an activated phenotype due to loss of suppressor of cytokine signaling 3, SOCS3, and subsequently increased JAK–STAT signaling in astrocytes (Huang *et al*, 2016b). Thus, reactive gliosis may require dynamic regulation of Yap1 activity to allow for temporally limited cell cycle entry.

Compared with its role in regulating adult NSC activity, Yap1 activity appears to play an even more prominent role during embryonic neurogenesis (Cappello *et al*, 2013; Lavado *et al*, 2018, 2021; Kostic *et al*, 2019; Han *et al*, 2020; Mukhtar *et al*, 2020; Najas *et al*, 2020). Using Yap1 loss-of-function approaches, Kostic *et al* observed a significant reduction in the proliferation of basal progenitors in ferret and human cortex. Intriguingly, there seems to be an increase in nuclear Yap1 levels alongside an increase in outer radial glia (ORG) from lissencephalic (mouse) to gyrencephalic (ferret and human) brains, implicating differential regulation of Yap1 activity in brain evolution. Recent data suggest a close relationship between ORG and adult hippocampal NSCs (Berg *et al*, 2019). Future studies may address whether differential Yap1 activity contributes to differences in initial pool size, maintenance, activation rates, and age-dependent exhaustion of adult hippocampal NSCs across mammalian species.

Consistent with a role of Yap1 in inducing NSC activation, scRNAseq from Yap1-5SA-expressing NSCs and astrocytes revealed a reduction in the expression of genes normally transcribed in quiescent NSCs and a concomitant increase in genes related to cell cycle and NSC activation. However, we noted that both on day 3 and day 7, transcriptomes of Yap1-5SA-expressing cells deviated substantially from the trajectory leading to neurons, indicative of a more profound and most likely aberrant reprogramming of gene expression. One particularly intriguing observation was the conspicuous absence of Ascl1 expression in Yap1-5SA-expresing cells. The onset of Ascl1 expression is a hallmark of adult hippocampal NSC activation and its genetic deletion results in NSC activation failure (Andersen *et al*, 2014; Urbán *et al*, 2016). While our data indicate that Ascl1 expression may not be an absolute prerequisite for entering cell cycle, it is likely critical for endowing proliferative NSCs with a neurogenic program. It would be interesting to learn in future how Yap1 gain-of-function results in the suppression of Ascl1 induction. ASCL1 expression was reported to be negatively correlated with YAP and TAZ expression in large-cell neuroendocrine carcinoma and small-cell lung cancer cells (Horie *et al*, 2016). Conversely, proneural factors such as Neurog2 and Ascl1 inhibit Yap1 nuclear localization by triggering phosphorylation of Yap1 at S112 (Zhang *et al*, 2012). This may suggest that Yap1 and Ascl1 exert a cross-inhibitory effect on each other. Intriguingly, instead of neurogenesis, Yap1-5SA-expressing cells may engage with various alternative differentiation programs as indicated by pseudotime analysis incorporating RNA splicing information. Partial differentiation of Yap1-5SA-expressing cells is further supported by the decrease in the expression of cell cycle-relevant genes despite an elevated Yap1 activity signature. One peculiar observation is that Yap1-5SA-expressing cells can adopt distinct differentiated states, highlighted by the differential expression of Scg2 and Pmp22 in subpopulations of Yap1-5SA-expressing cells. Also, cluster 1 appeared to be another (less clearly segregated) endpoint in pseudotime, enriched in collagen gene expression, suggestive of the acquisition of a more mesenchymal phenotype. However, none of these states could be directly identified with existing cell types. Whatever, the differentiation programs induced in Yap1-5SA-expressing cells, canonical lineage progression towards neurogenesis was severely disrupted. This suggests that sustained Yap1 activity occludes a canonical neurogenic program, which is in line with the fact that Yap1 activity signature and Yap1 protein expression were downregulated in differentiating immature neurons. Moreover, prolonged Yap1 gain-of-function in astrocytes seems to drive these cells away from their native lineage. While entirely conceivable, our scRNAseq analysis did not allow us to distinguish whether specific Yap1 gain-of-function cell clusters were preferentially or selectively generated from adult hippocampal NSCs or hilar astrocytes.

The highly aberrant transcriptional programs elicited by Yap1 gain-of-function may reflect processes that occur during glioblastoma formation. Work by Castellan *et al* (2021) recently showed that Yap1 and Taz are master regulators of stemness in glioblastoma stem-like cells (GSCs) and their gain-of-function represent a roadblock to GSC differentiation. Indeed, several of the genes identified by Castellan *et al* as components of the Yap/Taz regulon in GSCs were also induced following Yap1-5SA expression in hippocampal NSCs and/or astrocytes in our study (Fig 4F). This included the Yap1 paralogue Taz (Wwtr1) itself, as well as FoxO1 and Fos. Consistent with these findings, Yap1-5SA-expressing cells exhibited significant levels of expression of glioblastoma stem cell-related genes (Fig 4F). Future studies may reveal whether dysregulation of normally tightly regulated Yap1 activity induces the conversion of adult NSCs or postmitotic astrocytes into deadly GSCs.

Material and Methods

Animals and tamoxifen treatment

The study was performed in accordance with the guidelines of the German Animal Welfare Act and the European Directive 2010/63/EU for the protection of animals used for scientific purposes and was approved by the Rhineland-Palatinate State Authority (permit number 23177 07-G15-1-031 and 23177-07-G22-1-007). Mice were housed on a 12-h light/dark cycle in standard cages with free access to food and water. The study was conducted in both male and female mice. Wild-type C57BL/6J mice were purchased from Janvier at 8 weeks of age (Janvier Labs). Yap1 conditional knockout mice (*GlastCre*^{ERT2}; *Yap1* $^{flox/flox}$; *CAG-CAT-EGFP*, referred to as Yap1 cKO) were generated by crossing the *GlastCre*^{ERT2} (kind gift from Magdalena Götz, Helmholtz Zentrum München) with *Yap1* $^{flox/flox}$ (Jackson laboratory Stock NO. 027929) and *CAG-CAT-EGFP* mouse lines.

Tamoxifen (Sigma, T5648) was dissolved in corn oil with 10% ethanol to prepare a 20 mg/ml solution and administered once daily to both control (*GlastCre*^{ERT2}*; Yap1*$^{wt/wt}$*; CAG-CAT-EGFP*) and Yap1 cKO mice via intraperitoneal injections with 100 mg/kg for 5 consecutive days. Mice were sacrificed at indicated times as described in this article. Quantifications of radial glia-like cells (RGLs) in Figs 2E, F, H, and I, and EV3B were performed double-blind.

Tissue preparation

Mice were deeply anesthetized with a combination of 200 mg/kg Ketamine (Zoetis) and 20 mg/kg Xylazine (Bayer) (in 0.9% NaCl, i.p.) and transcardially perfused with saline, followed by 4% paraformaldehyde (PFA, Sigma, P6148). Brains were harvested and postfixed in 4% PFA overnight at 4°C. Coronal brain sections were prepared at a thickness of 50 μm using a Thermo Scientific vibrating blade microtome (Microm HM650V). Brain sections were stored in a cryoprotective solution (20% glucose, 40% ethylene glycol, 0.025% sodium azide, and 0.05 M phosphate buffer, pH 7.4) at −20°C.

Plasmids cloning and lentivirus production

To generate lentiviral destination plasmids (LV-hGFAP-IRES-EGFP, LV-hGFAP-Yap1-IRES-EGFP, LV-hGFAP-Yap1(5SA)-IRES-EGFP), Yap1 and Yap1-5SA with attB sites were amplified PCR (polymerase chain reaction) from pQCXIH-Myc-Yap1(5SA) (a kind gift from Kunliang Guan, Addgene plasmid # 33093) and pcDNA3-Yap1 (a kind gift from Stefano Piccolo, University of Padova). The amplified fragments were cloned into pDNR221 to generate p-entry plasmids using Gateway cloning (ThermoFisher Scientific). Lentiviral destination plasmids were generated by the LR recombination between p-entry and destination plasmids.

Lentivirus production was performed as described previously (Tiscornia *et al*, 2006). Second-generation lentiviral packaging plasmids psPAX and VSV-G envelope expressing plasmid pMD2.G (kind gifts from Didier Trono, Addgene plasmid #12260 and #12259) were used for the virus production. Viral pellets were resuspended in phosphate-buffered saline (PBS) and stored at −80°C.

Lentivirus injection

Stereotactic injections of lentivirus were performed in 8-week-old C57BL/6J mice. Prior to stereotactic injections and on the day following the surgery, mice were treated with analgesics (Rimadyl®, Zoetis, 10 mg/kg of body weight in 0.9% NaCl) by subcutaneous injection and then anesthetized with a combination of 0.5 mg/kg Medetomidine (Pfizer), 5 mg/kg Midazolam (Hameln), and 0.05 mg/kg Fentanyl (Albrecht) (in 0.9% NaCl) by intraperitoneal injection. Mice were placed in a stereotactic frame and kept on an animal heating pad to control body temperature during surgery. A small craniotomy was performed and 1 μl of the lentivirus was slowly injected using a pulled glass capillary (Hirschmann, 9600105) into the dentate gyrus at the following coordinates relative to Bregma: −2.0 AP (anterior–posterior), 1.4 ML (medio-lateral), −2.0 DV (dorso-ventral). The capillary was left in place for another 5 min before withdrawal to allow diffusion of the virus. Then, anesthesia was antagonized by intraperitoneal injection of 2.5 mg/kg Atipamezol (Pfizer), 0.5 mg/kg Flumazenil (Hameln), and 0.1 mg/kg Buprenorphine (RB Pharmaceuticals) (in 0.9% NaCl). Animals were allowed to recover, returned to their home cages, and their physical condition was monitored daily. Rimadyl®, Zoetis 15 mg/kg was administered via drinking water for 2 days after surgery.

Adherent adult neural stem cell culture

Adult neural stem cell cultures were prepared from the DG of young adult (8-week-old) mice as previously reported with minor modifications (Babu *et al*, 2011). Briefly, DG was dissected from an adult mouse brain in calcium and magnesium-free Hank's Balanced Salt Solution (HBSS, ThermoFisher Scientific, cat# 14170112), and the tissue was enzymatically dissociated using the Neural Tissue Dissociation Kit (Miltenyi Biotec, Germany, cat# 130-092-628) according to the manufacturer's instructions. For the fluorescence-activated cell sorting (FACS), the cell pellet was resuspended with PBS containing 5% fetal bovine serum (FBS, Life Technology, cat# 10270-106).

For the cell culture, the cells were purified as previously described (Ortega *et al*, 2013). The cell pellet was finally resuspended in Neurobasal Glutamax (Invitrogen, cat# 21103049) supplemented with B27 (Invitrogen, cat# 17504001), 20 ng/ml epidermal growth factor (EGF, Peprotech, cat#315-09), 20 ng/ml fibroblast growth factors (FGF2, Peprotech, cat# 100-18B), 100 units/ml penicillin and 100 μg/ml streptomycin (Invitrogen, cat# 15140122), and cells were plated in Poly-D-lysine hydrobromide (PDL, Sigma, cat# P0899) precoated 24-well plates (VWR, cat# 7340020). For the NSC passaging, the cells were trypsinized using 0.05% Trypsin–EDTA (ThermoFisher Scientific, cat# 25300054) for 5 min at 37°C. Neural stem cells were used for experiments for up to 10 passages.

Immunohistochemistry and immunocytochemistry

For immunohistochemistry, brain sections were blocked with 5% donkey serum (Sigma, S30) and 0.5% Triton-100 (Sigma, cat# X100-500 ml) in Tris-buffered saline (TBS, 50 mM Tris–Cl, pH 7.5; 150 mM NaCl) and stained with primary antibodies overnight at 4°C followed by secondary antibodies for 1 h at room temperature. Primary antibodies used in this study were as follows: mouse anti-Ascl1 (1:100, Santa Cruz, sc-374550), goat anti-DCX (1:300, Santa Cruz, sc-8066), guinea pig anti-DCX (1:500, Millipore, AB2253), mouse anti-GFAP (1:400, Millipore, MAB360), goat anti-GFAP (1:800, Abcam, ab53554), chicken anti-GFP (1:1,000, Aves Lab, GFP-1020), rabbit anti-Hopx (1:500, Proteintech, 11419-1-AP), mouse anti-Ki67 (1:200, BD Biosciences, 556003), rabbit anti-Mcm2 (1:800, Cell Signaling, 4007S), rabbit anti-Olig2 (1:1,500, Millipore, AB9610), goat anti-Sox2 (1:500, Santa Cruz, sc-17320), rabbit anti-Sox2 (1:500, Abcam, ab137385), rabbit anti-Tbr2 (1:200, Abcam, ab23345), rabbit anti-Yap1 (1:200, Cell Signaling, 14074S), mouse anti-Yap1 (1:200, Sigma, WH0010413M1). Secondary antibodies used were as follows: donkey anti-chicken Alexa 488 (1:200, Jackson ImmunoResearch, 703545155), donkey anti-goat Cy3 (1:500, Dianova, 705165147), donkey anti-goat Cy5 (1:300, Dianova, 705165147), donkey anti-Guinea pig Cy5 (1:300, Dianova, 705175148), donkey anti-mouse Alexa 647 (1:300, Invitrogen, A31571), donkey anti-rabbit Cy3 (1:500, Dianova, 711165152).

Images were acquired with a LEICA TCS SPE confocal microscope equipped with a 40× oil objective (NA 1.3) or an upright ZEISS Axio Imager.M2 epifluorescent microscope equipped with a

© 2023 The Authors

40× dry objective (NA 0.75) and an ApoTome.2 system (Zeiss GmbH). 4–5 images were taken from every brain for quantifications. Images were analyzed using Image J software (NIH).

For immunocytochemistry, adult neural stem cells were fixed in 4% PFA for 10 min at room temperature, followed by washing with PBS for 3 times. Cells were blocked with 2% bovine serum albumin (BSA) (Sigma, A9418) and 0.2% Triton-100 for 45 min in TBS and incubated with primary antibodies overnight at 4°C followed by incubation with secondary antibodies for 1 h at room temperature. For BrdU (5-bromo-2′-deoxyuridine, Sigma, cat# B5002) staining, cells were incubated in 2 N HCl at 37°C for 10 min, followed by 0.1 M boric acid for 10 min at room temperature. EdU (5-ethynyl-2′-deoxyuridine, ThermoFisher Scientific, cat# A10044) staining was performed by Click-it Alexa Fluor 647 imaging kit (Thermo Fisher Scientific, cat# C10086) according to the manufacturer's instructions. Primary antibodies used in this study were as follows: rat anti-BrdU (1:500, ABD Serotec, OBT0030), chicken anti-GFP (1:1,000, Aves Lab, GFP-1020), mouse anti-Ki67 (1:200, BD Biosciences, 556,003), rabbit anti-Nestin (1:500, BioLegend, 839801), rabbit anti-PH3 (1:300, Millipore, 06570), rabbit anti-Yap1 (1:200, Cell Signaling, 14074S), mouse anti-Yap1 (1:200, Sigma, WH0010413M1). Secondary antibodies used were as follows: donkey anti-chicken Alexa 488 (1:1,000, Jackson ImmunoResearch, 703545155), donkey anti-rabbit Cy3 (1:1,000, Dianova, 711165152), donkey anti-rat Alexa 547 (1:1,000, Interchim, FP-SB6120).

Images of immunostained cells in culture were acquired with an upright ZEISS Axio Imager.M2 epifluorescent microscope equipped with 40× dry objective (NA 0.75) and an ApoTome.2 system (Zeiss GmbH). 4–5 images were selected randomly based on the presence of DAPI staining from each independent experiment for quantification. Images were analyzed using Image J software (NIH).

Flow cytometry

For flow cytometry of lentivirus-transduced cells, animals were sacrificed by cervical dislocation and their brains immediately placed on ice. The DG was microdissected and the tissue was enzymatically dissociated using the Neural Tissue Dissociation Kit (Miltenyi Biotec, Germany, cat# 130-092-628) according to the manufacturer's instructions. Sytox Blue (1/1000, Life Technologies, cat# S34857) was added to the cell suspension prior to sorting to exclude dead cells. Gates and compensations were set before every experiment using control samples without any fluorochromes. Single cell suspensions were sorted in a FACSAria II SORP (BD Bioscience) using a 100 μm nozzle at 20 psi. EGFP-positive cells from control and Yap1-5SA groups were directly collected into ice-cold lysis buffer (0.2%Triton X-100, containing RNAse inhibitors, Clontech, cat# 635013) in 96-well plates (Eppendorf, cat# 0030128680), and frozen at −80°C until library preparation.

Library preparation and single cell RNA sequencing

An adapted protocol from the Smart-seq2 method (Picelli *et al*, 2014) was used. Upon thawing the plates on ice, reverse transcription was performed using an oligo(dT) primer and a locked nucleic acid (LNA)-containing template-switching oligonucleotide (IDT; custom oligo). For all samples, we included ERCC spike-in controls (Ambion, cat#4456740) at a 1:10^6 dilution. Full-length cDNAs were amplified by 18 cycles of PCR using KAPA HiFi DNA polymerase (Roche, cat#7958935001). Amplified cDNAs for a set of randomly selected samples were quantified and run on a High Sensitivity Bioanalyzer chip (Agilent, cat# 5067-4626) on a 2,100 Bioanalyzer (Agilent).

Libraries were prepared using the Illumina Nextera XT DNA sample preparation kit (Illumina, cat#FC-131-1096) with a minor modification. The modification is based on the tagmentation protocol from Fluidigm, which uses 25% of the reagents per tagmentation reaction with a starting amount of 100–125 pg of cDNA. Libraries were amplified in 12 PCR cycles. Three microliters of each library were pooled into a single tube and the pool was subsequently bead-purified using Agencout AMPure XP beads (Beckman Coulter GmbH, cat# A63882). The purified pool was profiled in a High Sensitivity DNA chip on a 2100 Bioanalyzer (Agilent) and quantified using the Qubit dsDNA HS Assay Kit (Invitrogen, cat#Q32854) in a Qubit 2.0 Fluorometer (Life technologies). Four pools (each one from one of four 96-well plates) were pooled together at equal concentrations and sequenced on a single NextSeq 500 High Output Flow Cell with single reads for 1 × 75 cycles for read 1 plus 2 × 8 cycles for each index read.

Single cell RNA-sequencing data processing

All downstream analysis was performed using the open-source R software accessed via RStudio server (R version 3.6.3). Smart-Seq2 raw data demultiplexing were performed using Illumina's bcl2fastq conversion software v.2.19.1 and overall sequence quality was assessed with FastQC v0.11.5. STAR v.2.5.2b with default parameters (except –outFilterMismatchNoverLmax 0.04) was used to align reads to the mouse reference genome GRCm38 (mm10), supplemented with ERCC RNA Spike-In Mix (ThermoFischer) control sequences and considering the GTF gene annotation from Ensembl release 88. Read summarization at the gene level was performed using Subread featureCounts v.1.6.2 with default parameters.

The quality control and normalization pipeline were applied as previously described (Amezquita *et al*, 2020). Briefly, cells with log-transformed library sizes and number of genes below 3 median absolute deviations (MADs) from the median of the respective distributions were removed. In addition, we removed cells in which the proportion of mitochondrial genes and spike-in RNAs was 3 MADs above the respective median proportions. Lastly, genes with average counts across samples below a threshold of 1 were filtered out.

Single cell transcriptome analysis

Using python (3.8.1) and standard python libraries (ipykernel 5.3.0. matplotlib 3.2.2, numpy 1.17.3, pandas 1.0.5, scipy 1.4.1), we employed scanpy (1.5.1), anndata (0.7.3), and anndata2ri (1.0.2) (Wolf *et al*, 2018) to perform single cell analysis. We filtered out cells with < 500 or more than 6,000 genes detected and cells with < 5% mitochondrial genes. Moreover, we filtered out genes that are expressed in < 10 cells. After normalization and log transformation, we regressed out differences in cell cycle scores, percent mitochondrial genes, number of transcripts per cell, and percentage of spike-in. We identified highly variable genes using default parameters, which were further used for exploratory analysis via principal component analysis and force-directed graph embedding (python-igraph

© 2023 The Authors

0.8.2). Clustering of cells was done using the Leiden algorithm (0.8.1) implementation within scanpy. Differential gene expression analysis between Leiden clusters was performed using function sc.tl.rank_genes_groups with Wilcoxon testing. For quantification of RNA splicing from the raw sequencing data, we used velocyto (0.17) (La Manno *et al*, 2018) with default smartseq2 parameters. Subsequently, we employed scvelo (0.2.3) (Bergen *et al*, 2020) for RNA velocity estimations using the stochastic algorithm with default parameters after recovering the dynamics and using latent time. For the estimation of a trajectory pseudotime, we employed the diffusion pseudotime algorithm implementation in scvelo.

The analysis of adult hippocampal dataset (Data ref: Hochgerner *et al*, 2018) was performed using the open-source R software accessed via RStudio server (R version 3.6.3). Briefly, normalization was performed using SCTransform with default parameters in Seurat (v3.1). We selected different cut-offs of the number of PCs and empirically found that downstream clustering analyses were optimized when using a 15-PC cutoff. The first 15 PCs were selected and used for two-dimension Uniform Manifold Approximation and Projection (UMAP), implemented by the Seurat software with default parameters. Based on the UMAP map, 5 clusters were identified using the function FindClusters in Seurat with the resolution parameter of 0.5. Radial glia-like cells, astrocytes, nIPCs, and neuroblasts were identified using FindAllMakers with default parameters. Differentially expressed genes that were expressed in at least 25% of cells within the cluster and with a fold change of more than 0.5 (log scale) were considered to be marker genes. To calculate Yap signature and active signature, AddModuleScore function was used in Seurat with default parameters.

Statistics

Statistical analysis was performed by a two-tailed unpaired Student's *t*-test using GraphPad Prism version 7 (GraphPad Software, Inc.). Data are represented as mean ± SEM. *P*-values are indicated in the graphs.

Data availability

Sequencing data have been deposited at GEO accession GSE144967. Source data are provided with this paper. Additional data related to this paper can be requested from the authors.

Expanded View for this article is available online.

Acknowledgements

This research was funded in part by the Wellcome Trust (206410/Z/17/Z) to BB. For the purpose of open access, the author has applied a CC BY public copyright license to any author-accepted manuscript version arising from this submission. This study was also supported by funding from the German Research Foundation (DFG, CRC1080, project number 221828878, CRC1193 project number 26410226) to BB; core funding to the Francis Crick Institute from Cancer Research UK, The Medical Research Council and the Wellcome Trust (FC001002); funding from the Naturwissenschaftlich-Medizinischen Forschungszentrum (NMFZ2016) of the Johannes Gutenberg University Mainz to CB. JJ-A was supported by research fellowships from Fundación Alfonso Martín Escudero, Fundación Ramón Areces, and the European Commission (MSCA-IF-841260). We are grateful to Flow Cytometry Core Facility, Genomic Core Facility, Microscopy Core Facility, and Bioinformatics Core Facility of the Institute of Molecular Biology (IMB) Mainz for their support regarding single cell RNA-sequencing experiments and raw data processing. Parts of the schematic diagram were created with BioRender.com. Open Access funding enabled and organized by Projekt DEAL.

Author contributions

Benedikt Berninger: Conceptualization; supervision; funding acquisition; writing – original draft; project administration; writing – review and editing. **Wenqiang Fan:** Conceptualization; data curation; formal analysis; investigation; visualization; methodology; writing – original draft; writing – review and editing. **Jerónimo Jurado-Arjona:** Conceptualization; data curation; formal analysis; funding acquisition; investigation; visualization; methodology; writing – original draft; writing – review and editing. **Gregorio Alanis-Lobato:** Formal analysis; investigation; visualization; methodology; writing – review and editing. **Sophie Péron:** Data curation; methodology; project administration; writing – review and editing. **Christian Berger:** Conceptualization; resources; funding acquisition; writing – review and editing. **Miguel A Andrade-Navarro:** Formal analysis; methodology; writing – review and editing. **Sven Falk:** Formal analysis; investigation; methodology; writing – review and editing.

Disclosure and competing interests statement

The authors declare that they have no conflict of interest.

References

Amezquita RA, Lun AT, Becht E, Carey VJ, Carpp LN, Geistlinger L, Marini F, Rue-Albrecht K, Risso D, Soneson C (2020) Orchestrating single-cell analysis with Bioconductor. *Nat Methods* 17: 137–145

Andersen J, Urbán N, Achimastou A, Ito A, Simic M, Ullom K, Martynoga B, Lebel M, Göritz C, Frisén J (2014) A transcriptional mechanism integrating inputs from extracellular signals to activate hippocampal stem cells. *Neuron* 83: 1085–1097

Babu H, Claasen J-H, Kannan S, Rünker AE, Palmer T, Kempermann G (2011) A protocol for isolation and enriched monolayer cultivation of neural precursor cells from mouse dentate gyrus. *Front Neurosci* 5: 89

Berg DA, Su Y, Jimenez-Cyrus D, Patel A, Huang N, Morizet D, Lee S, Shah R, Ringeling FR, Jain R (2019) A common embryonic origin of stem cells drives developmental and adult neurogenesis. *Cell* 177: 654–668.e15

Bergen V, Lange M, Peidli S, Wolf FA, Theis FJ (2020) Generalizing RNA velocity to transient cell states through dynamical modeling. *Nat Biotechnol* 38: 1408–1414

Cappello S, Gray MJ, Badouel C, Lange S, Einsiedler M, Srour M, Chitayat D, Hamdan FF, Jenkins ZA, Morgan T (2013) Mutations in genes encoding the cadherin receptor-ligand pair DCHS1 and FAT4 disrupt cerebral cortical development. *Nat Genet* 45: 1300–1308

Castellan M, Guarnieri A, Fujimura A, Zanconato F, Battilana G, Panciera T, Sladitschek HL, Contessotto P, Citron A, Grilli A (2021) Single-cell analyses reveal YAP/TAZ as regulators of stemness and cell plasticity in glioblastoma. *Nat cancer* 2: 174–188

Cordenonsi M, Zanconato F, Azzolin L, Forcato M, Rosato A, Frasson C, Inui M, Montagner M, Parenti AR, Poletti A (2011) The hippo transducer TAZ confers cancer stem cell-related traits on breast cancer cells. *Cell* 147: 759–772

 © 2023 The Authors

Ding R, Weynans K, Bossing T, Barros CS, Berger C (2016) The Hippo signalling pathway maintains quiescence in *Drosophila* neural stem cells. *Nat Commun* 7: 1 – 12

Dulken BW, Buckley MT, Negredo PN, Saligrama N, Cayrol R, Leeman DS, George BM, Boutet SC, Hebestreit K, Pluvinage JV (2019) Single-cell analysis reveals T cell infiltration in old neurogenic niches. *Nature* 571: 205 – 210

Engler A, Rolando C, Giachino C, Saotome I, Erni A, Brien C, Zhang R, Zimber-Strobl U, Radtke F, Artavanis-Tsakonas S (2018) Notch2 signaling maintains NSC quiescence in the murine ventricular-subventricular zone. *Cell Rep* 22: 992 – 1002

Gil-Ranedo J, Gonzaga E, Jaworek KJ, Berger C, Bossing T, Barros CS (2019) STRIPAK members orchestrate hippo and insulin receptor signaling to promote neural stem cell reactivation. *Cell Rep* 27: e5

Hamon A, García-García D, Ail D, Bitard J, Chesneau A, Dalkara D, Locker M, Roger JE, Perron M (2019) Linking YAP to Müller glia quiescence exit in the degenerative retina. *Cell Rep* 27: e6

Han J, Calvo C-F, Kang TH, Baker KL, Park J-H, Parras C, Levittas M, Birba U, Pibouin-Fragner L, Fragner P (2015) Vascular endothelial growth factor receptor 3 controls neural stem cell activation in mice and humans. *Cell Rep* 10: 1158 – 1172

Han D, Lee SM, Kwon M, Noh H, Lee JH, Yoon Y, Cho JY, Yoon K (2020) YAP enhances FGF2-dependent neural stem cell proliferation by induction of FGF receptor expression. *Stem Cells Dev* 29: 1240 – 1246

Hao Y, Hao S, Andersen-Nissen E, Mauck WM III, Zheng S, Butler A, Lee MJ, Wilk AJ, Darby C, Zager M (2021) Integrated analysis of multimodal single-cell data. *Cell* 184: 3573 – 3587.e29

Harada Y, Yamada M, Imayoshi I, Kageyama R, Suzuki Y, Kuniya T, Furutachi S, Kawaguchi D, Gotoh Y (2021) Cell cycle arrest determines adult neural stem cell ontogeny by an embryonic notch-nonoscillatory Hey1 module. *Nat Commun* 12: 1 – 16

Harris L, Rigo P, Stiehl T, Gaber ZB, Austin SH, del Mar Masdeu M, Edwards A, Urbán N, Marciniak-Czochra A, Guillemot F (2021) Coordinated changes in cellular behavior ensure the lifelong maintenance of the hippocampal stem cell population. *Cell Stem Cell* 28: e6

Hochgerner H, Zeisel A, Lönnerberg P, Linnarsson S (2018) Gene Expression Omnibus GSE95753 (https://www.ncbi.nlm.nih.gov/geo/query/acc.cgi?acc=GSE95753). [DATASET]

Horie M, Saito A, Ohshima M, Suzuki HI, Nagase T (2016) YAP and TAZ modulate cell phenotype in a subset of small cell lung cancer. *Cancer Sci* 107: 1755 – 1766

Huang Z, Hu J, Pan J, Wang Y, Hu G, Zhou J, Mei L, Xiong W-C (2016a) YAP stabilizes SMAD1 and promotes BMP2-induced neocortical astrocytic differentiation. *Development* 143: 2398 – 2409

Huang Z, Wang Y, Hu G, Zhou J, Mei L, Xiong W-C (2016b) YAP is a critical inducer of SOCS3, preventing reactive astrogliosis. *Cereb Cortex* 26: 2299 – 2310

Ibrayeva A, Bay M, Pu E, Jörg DJ, Peng L, Jun H, Zhang N, Aaron D, Lin C, Resler G (2021) Early stem cell aging in the mature brain. *Cell Stem Cell* 28: e7

Kalamakis G, Brüne D, Ravichandran S, Bolz J, Fan W, Ziebell F, Stiehl T, Catalá-Martinez F, Kupke J, Zhao S (2019) Quiescence modulates stem cell maintenance and regenerative capacity in the aging brain. *Cell* 176: 1407 – 1419.e14

Kawai H, Kawaguchi D, Kuebrich BD, Kitamoto T, Yamaguchi M, Gotoh Y, Furutachi S (2017) Area-specific regulation of quiescent neural stem cells by Notch3 in the adult mouse subependymal zone. *J Neurosci* 37: 11867 – 11880

Kostic M, Paridaen JT, Long KR, Kalebic N, Langen B, Grübling N, Wimberger P, Kawasaki H, Namba T, Huttner WB (2019) YAP activity is necessary and sufficient for basal progenitor abundance and proliferation in the developing neocortex. *Cell Rep* 27: e6

La Manno G, Soldatov R, Zeisel A, Braun E, Hochgerner H, Petukhov V, Lidschreiber K, Kastriti ME, Lönnerberg P, Furlan A (2018) RNA velocity of single cells. *Nature* 560: 494 – 498

Lavado A, Park JY, Paré J, Finkelstein D, Pan H, Xu B, Fan Y, Kumar RP, Neale G, Kwak YD (2018) The hippo pathway prevents YAP/TAZ-driven hypertranscription and controls neural progenitor number. *Dev Cell* 47: 576 – 591.e8

Lavado A, Gangwar R, Paré J, Wan S, Fan Y, Cao X (2021) YAP/TAZ maintain the proliferative capacity and structural organization of radial glial cells during brain development. *Dev Biol* 480: 39 – 49

Lie D-C, Colamarino SA, Song H-J, Désiré L, Mira H, Consiglio A, Lein ES, Jessberger S, Lansford H, Dearie AR (2005) Wnt signalling regulates adult hippocampal neurogenesis. *Nature* 437: 1370 – 1375

Martynoga B, Mateo JL, Zhou B, Andersen J, Achimastou A, Urbán N, van den Berg D, Georgopoulou D, Hadjur S, Wittbrodt J (2013) Epigenomic enhancer annotation reveals a key role for NFIX in neural stem cell quiescence. *Genes Dev* 27: 1769 – 1786

Mira H, Andreu Z, Suh H, Lie DC, Jessberger S, Consiglio A, San Emeterio J, Hortigüela R, Marqués-Torrejón MÁ, Nakashima K (2010) Signaling through BMPR-IA regulates quiescence and long-term activity of neural stem cells in the adult hippocampus. *Cell Stem Cell* 7: 78 – 89

Mo JS, Park HW, Guan KL (2014) The hippo signaling pathway in stem cell biology and cancer. *EMBO Rep* 15: 642 – 656

Mori T, Tanaka K, Buffo A, Wurst W, Kühn R, Götz M (2006) Inducible gene deletion in astroglia and radial glia—a valuable tool for functional and lineage analysis. *Glia* 54: 21 – 34

Moya IM, Halder G (2019) Hippo–YAP/TAZ signalling in organ regeneration and regenerative medicine. *Nat Rev Mol Cell Biol* 20: 211 – 226

Mukhtar T, Breda J, Grison A, Karimaddini Z, Grobecker P, Iber D, Beisel C, van Nimwegen E, Taylor V (2020) Tead transcription factors differentially regulate cortical development. *Sci Rep* 10: 1 – 19

Najas S, Pijuan I, Esteve-Codina A, Usieto S, Martinez JD, Zwijsen A, Arbonés ML, Martí E, Le Dréau G (2020) A SMAD1/5-YAP signalling module drives radial glia self-amplification and growth of the developing cerebral cortex. *Development* 147: dev187005

Nakamura T, Colbert MC, Robbins J (2006) Neural crest cells retain multipotential characteristics in the developing valves and label the cardiac conduction system. *Circ Res* 98: 1547 – 1554

Ortega F, Gascón S, Masserdotti G, Deshpande A, Simon C, Fischer J, Dimou L, Chichung Lie D, Schroeder T, Berninger B (2013) Oligodendrogliogenic and neurogenic adult subependymal zone neural stem cells constitute distinct lineages and exhibit differential responsiveness to Wnt signalling. *Nat Cell Biol* 15: 602 – 613

Park R, Moon UY, Park JY, Hughes LJ, Johnson RL, Cho S-H, Kim S (2016) Yap is required for ependymal integrity and is suppressed in LPA-induced hydrocephalus. *Nat Commun* 7: 1 – 14

Picelli S, Faridani OR, Björklund ÅK, Winberg G, Sagasser S, Sandberg R (2014) Full-length RNA-seq from single cells using smart-seq2. *Nat Protoc* 9: 171 – 181

Pilz G-A, Bottes S, Betizeau M, Jörg DJ, Carta S, Simons BD, Helmchen F, Jessberger S (2018) Live imaging of neurogenesis in the adult mouse hippocampus. *Science* 359: 658 – 662

Plouffe SW, Lin KC, Moore JL, Tan FE, Ma S, Ye Z, Qiu Y, Ren B, Guan K-L (2018) The hippo pathway effector proteins YAP and TAZ have both

© 2023 The Authors

distinct and overlapping functions in the cell. *J Biol Chem* 293: 11230–11240
Rueda EM, Hall BM, Hill MC, Swinton PG, Tong X, Martin JF, Poché RA (2019) The hippo pathway blocks mammalian retinal Müller glial cell reprogramming. *Cell Rep* 27: e6
Seri B, Garcıa-Verdugo JM, McEwen BS, Alvarez-Buylla A (2001) Astrocytes give rise to new neurons in the adult mammalian hippocampus. *J Neurosci* 21: 7153–7160
Shin J, Berg DA, Zhu Y, Shin JY, Song J, Bonaguidi MA, Enikolopov G, Nauen DW, Christian KM, Ming G-l (2015) Single-cell RNA-seq with waterfall reveals molecular cascades underlying adult neurogenesis. *Cell Stem Cell* 17: 360–372
Song J, Zhong C, Bonaguidi MA, Sun GJ, Hsu D, Gu Y, Meletis K, Huang ZJ, Ge S, Enikolopov G (2012) Neuronal circuitry mechanism regulating adult quiescent neural stem-cell fate decision. *Nature* 489: 150–154
Suh H, Consiglio A, Ray J, Sawai T, D'Amour KA, Gage FH (2007) *In vivo* fate analysis reveals the multipotent and self-renewal capacities of Sox2+ neural stem cells in the adult hippocampus. *Cell Stem Cell* 1: 515–528
Tiscornia G, Singer O, Verma IM (2006) Production and purification of lentiviral vectors. *Nat Protoc* 1: 241–245
Urbán N, van den Berg DL, Forget A, Andersen J, Demmers JA, Hunt C, Ayrault O, Guillemot F (2016) Return to quiescence of mouse neural stem cells by degradation of a proactivation protein. *Science* 353: 292–295
Urbán N, Blomfield IM, Guillemot F (2019) Quiescence of adult mammalian neural stem cells: a highly regulated rest. *Neuron* 104: 834–848
Wang X, Valls AF, Schermann G, Shen Y, Moya IM, Castro L, Urban S, Solecki GM, Winkler F, Riedemann L (2017) YAP/TAZ orchestrate VEGF signaling during developmental angiogenesis. *Dev Cell* 42: 462–478.e7
Wexler EM, Paucer A, Kornblum HI, Palmer TD, Geschwind DH (2009) Endogenous Wnt signaling maintains neural progenitor cell potency. *Stem Cells* 27: 1130–1141
Wolf FA, Angerer P, Theis FJ (2018) SCANPY: large-scale single-cell gene expression data analysis. *Genome Biol* 19: 1–5
Yu F-X, Guan K-L (2013) The hippo pathway: regulators and regulations. *Genes Dev* 27: 355–371
Zhang N, Bai H, David KK, Dong J, Zheng Y, Cai J, Giovannini M, Liu P, Anders RA, Pan D (2010) The Merlin/NF2 tumor suppressor functions through the YAP oncoprotein to regulate tissue homeostasis in mammals. *Dev Cell* 19: 27–38
Zhang H, Deo M, Thompson RC, Uhler MD, Turner DL (2012) Negative regulation of yap during neuronal differentiation. *Dev Biol* 361: 103–115
Zhang R, Boareto M, Engler A, Louvi A, Giachino C, Iber D, Taylor V (2019) Id4 downstream of Notch2 maintains neural stem cell quiescence in the adult hippocampus. *Cell Rep* 28: e6
Zhao B, Wei X, Li W, Udan RS, Yang Q, Kim J, Xie J, Ikenoue T, Yu J, Li L (2007) Inactivation of YAP oncoprotein by the hippo pathway is involved in cell contact inhibition and tissue growth control. *Genes Dev* 21: 2747–2761

License: This is an open access article under the terms of the Creative Commons Attribution License, which permits use, distribution and reproduction in any medium, provided the original work is properly cited.

© 2023 The Authors

Expanded View Figures

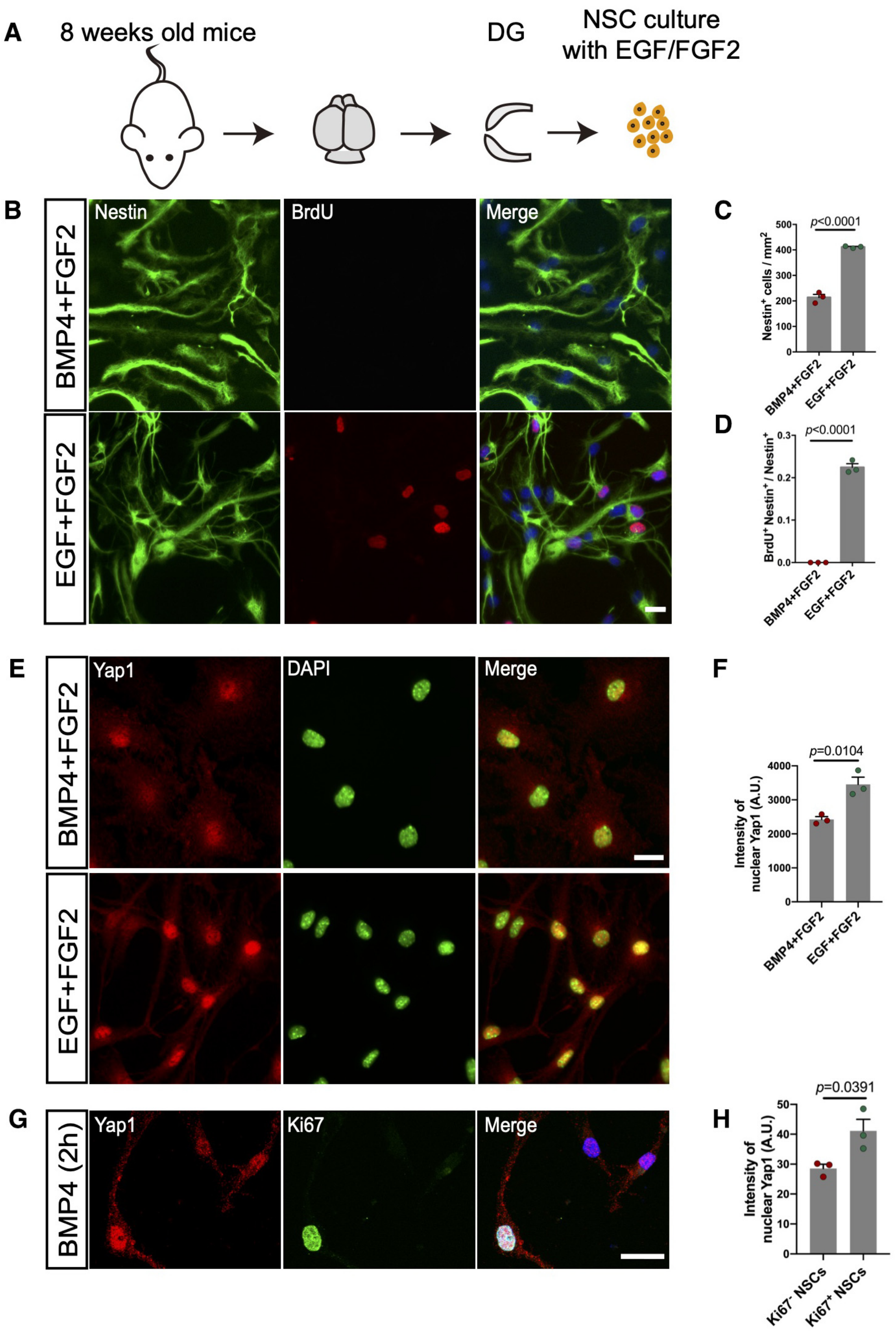

Figure EV1.

Figure EV1. Nuclear Yap1 level is increased upon adult NSCs activation.

A Schematic diagram of adult hippocampus derived NSCs culture.
B Cultured adult NSCs were treated with EGF + FGF2 (proliferative condition) or BMP4 + FGF2 (quiescent condition). Immunofluorescence for Nestin and BrdU indicates that BMP4 + FGF2 treatment efficiently blocks the cell division of adult NSCs, as evidenced by the fact that they did not incorporate the BrdU administered during the last 6 h prior to fixation.
C, D Quantification of the data in (B). No BrdU-positive cells were found in quiescent condition and resulted in a reduction of NSCs density. $n = 3$ (biological replicates) independent experiments.
E Immunofluorescence for Yap1 and DAPI in quiescent and proliferative NSCs.
F Quantification of the nuclear Yap1 intensity in (E). Nuclear Yap1 level increases upon activation of quiescent NSCs. $n = 3$ (biological replicates, 300 cells from three independent experiments) in proliferative condition, $n = 3$ (biological replicates, 249 cells from three independent experiments) in quiescent condition.
G Cultured adult NSCs were treated with BMP4 + FGF2 for 2 h. Immunofluorescence for Yap1 and Ki67 in proliferative and nonproliferative NSCs.
H Quantification of the data in (G). Proliferative NSCs (Ki67-positive) enriched higher level of nuclear Yap1. $n = 3$ (biological replicates, 85 Ki67-positive cells and 91 Ki67-negative cells from 3 independent experiments).

Data information: Data are represented as mean ± SEM. Unpaired Student's *t*-test. Scale bars: 20 μm.

Figure EV2. Conditional knockout Yap1 in adult NSCs at 30 dpi.

A Immunofluorescence for GFP (recombination reporter), GFAP, and Mcm2 in control and Yap1 cKO RGLs at 30 days after tamoxifen administration. Arrows indicate Mcm2-positive, proliferating RGLs.
B Quantification of the data in (A). Loss of Yap1 does not induce significant changes in the number of proliferative RGLs or the overall number of RGLs. $n = 3$ mice (biological replicates) for control group, $n = 4$ mice (biological replicates) for Yap1 cKO group.
C, D Immunofluorescence for GFP (recombination reporter) and DCX in control and Yap1 cKO RGLs at 30 and 60 days after tamoxifen administration. Arrows indicate DCX-positive immature neurons.
E, F Quantification of the data in (C and D). Loss of Yap1 does not induce significant changes in the number of immature neurons. 30 dpi group (E): $n = 3$ mice for control group, $n = 4$ mice (biological replicates) for Yap1 cKO group; 60 dpi group (F): $n = 4$ mice (biological replicates) for both control group and Yap1 cKO group.
G Immunofluorescence for GFP (recombination reporter), GFAP, and Yap1 in control and Yap1 cKO RGLs at 60 days after tamoxifen administration. Quantification of Yap1 intensity in recombined RGLs. Yap1 levels are significantly decreased in Yap1 cKO RGLs at 60 dpi. $n = 4$ (23 cells from four mice) in control group, $n = 4$ (21 cells from four mice) in Yap1 cKO group.
H Schematic diagram of experimental design.
I Immunofluorescence for GFP and EdU indicates that loss of Yap1 efficiently blocks the cell division of adult NSCs, as evidenced by the fact that they did not incorporate the EdU administered during the last 4 h prior to fixation. Quantification shown in the right panel. $n = 3$ independent experiments (biological replicates).

Data information: Data are represented as mean ± SEM. Unpaired Student's *t*-test. Scale bars: 20 μm in (A), (C), and (I); 10 μm in (D); 5 μm in (G).

© 2023 The Authors

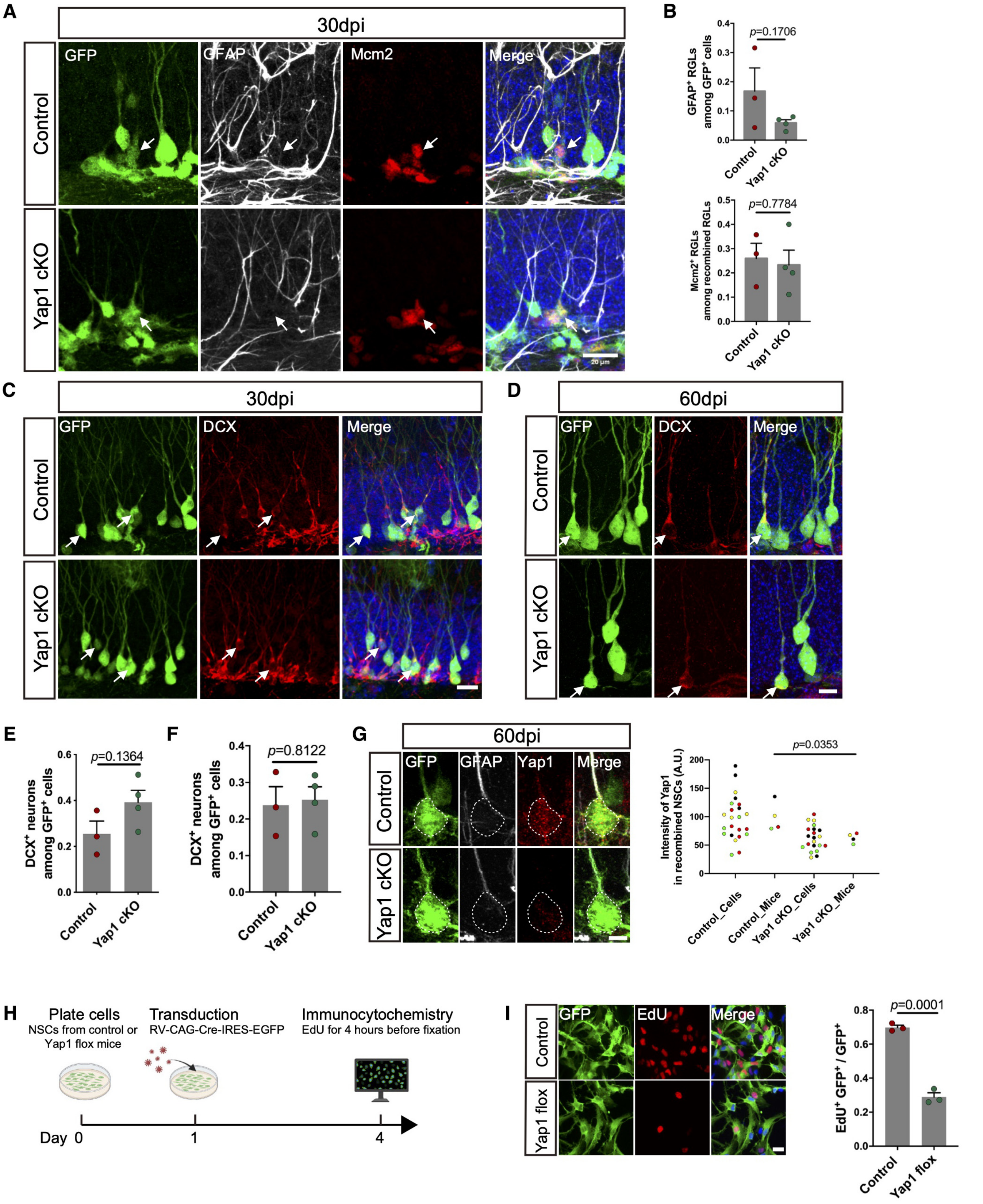
A
30dpi
GFP
GFAP
Mcm2
Merge
Control
Yap1 cKO
20 µm
B
p=0.1706
GFAP+ RGLs among GFP+ cells
p=0.7784
Mcm2+ RGLs among recombined RGLs
C
30dpi
GFP
DCX
Merge
D
60dpi
E
p=0.1364
DCX+ neurons among GFP+ cells
F
p=0.8122
G
60dpi
Yap1
p=0.0353
Intensity of Yap1 in recombined NSCs (A.U.)
Control_Cells
Control_Mice
Yap1 cKO_Cells
Yap1 cKO_Mice
H
Plate cells
NSCs from control or Yap1 flox mice
Transduction
RV-CAG-Cre-IRES-EGFP
Immunocytochemistry
EdU for 4 hours before fixation
Day 0
1
4
I
EdU
Yap1 flox
p=0.0001
EdU+ GFP+ / GFP+

Figure EV2.

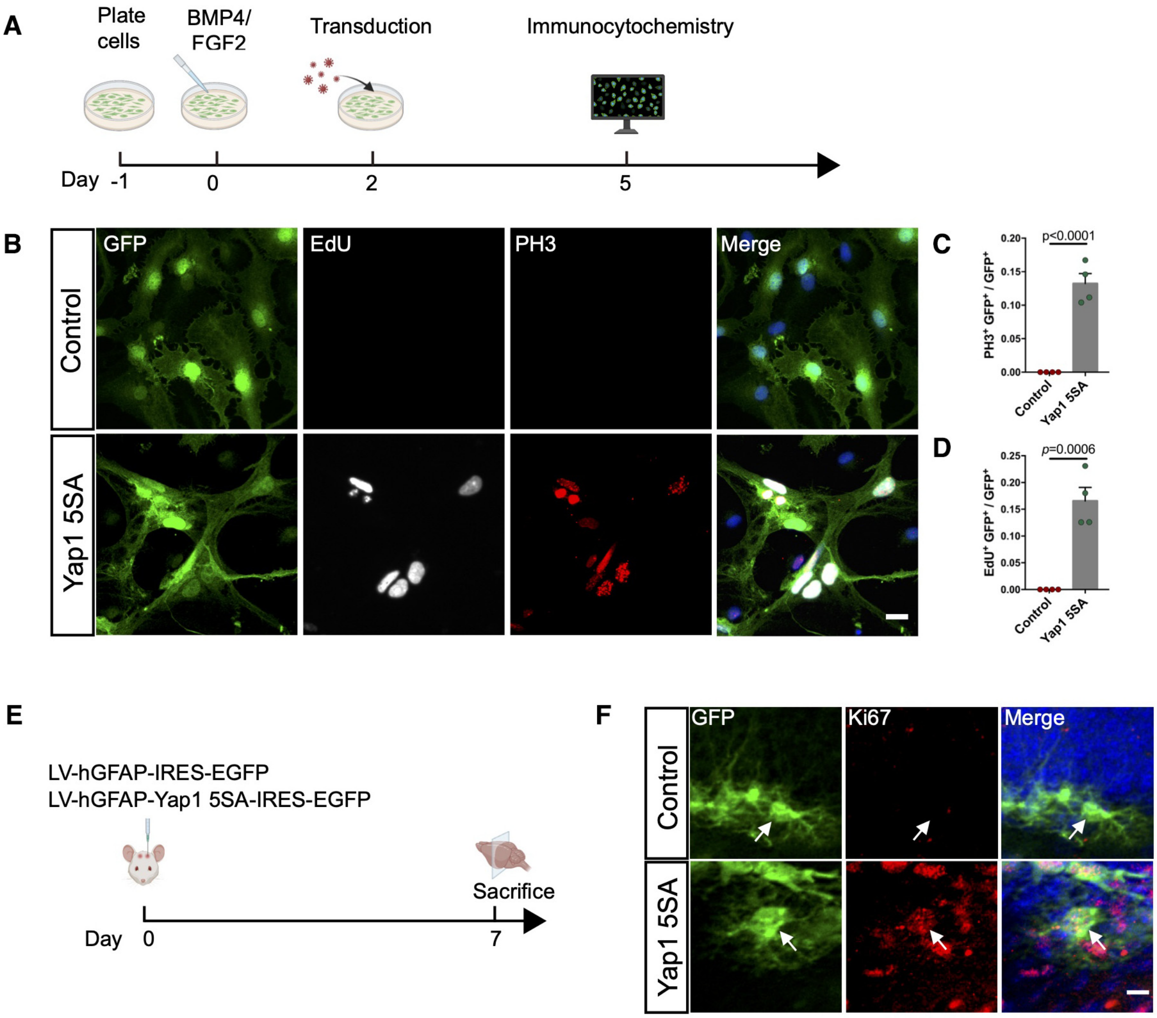

Figure EV3. Overexpression of Yap1-5SA induces the proliferation of quiescent adult NSCs *in vitro* and astrocytes *in vivo*.

A Schematic diagram of the experimental design for the analysis of the effect of Yap1 overexpression in quiescent NSCs *in vitro*.

B Immunofluorescence for GFP, EdU (administered 6 h before fixation), and PH3 (to identify proliferative cells) in control and Yap1-5SA overexpressing NSCs.

C, D Quantification of the data in (B). Yap1-5SA induces the proliferation of quiescent NSCs *in vitro*. $n = 4$ (biological replicates) independent cultures for each group.

E Schematic diagram of the experimental design for the analysis of the effect of Yap1 overexpression in astrocytes in hilus.

F Immunofluorescence for GFP and Ki67 in coronal brain sections. Arrows indicate astrocytes in hilus.

Data information: Data are represented as mean ± SEM. Unpaired Student's *t*-test. Scale bars: 20 μm.

Figure EV4. Clustering of single cell RNA-sequencing data and cell-type identification based on differential gene expression analysis.

A Experimental groups and 13 different cell clusters.

B Number of transcripts in both control and Yap1-5SA-expressing cells.

C-H Markers for each cell type including NSCs, astrocytes, dying cells, microglia, oligodendroglial cells, and neurons.

I Identification of top 10 markers for each cluster via differential gene expression analysis.

J Expression levels of cell cycle-related genes at 3 and 7 days after lentivirus injection.

 © 2023 The Authors

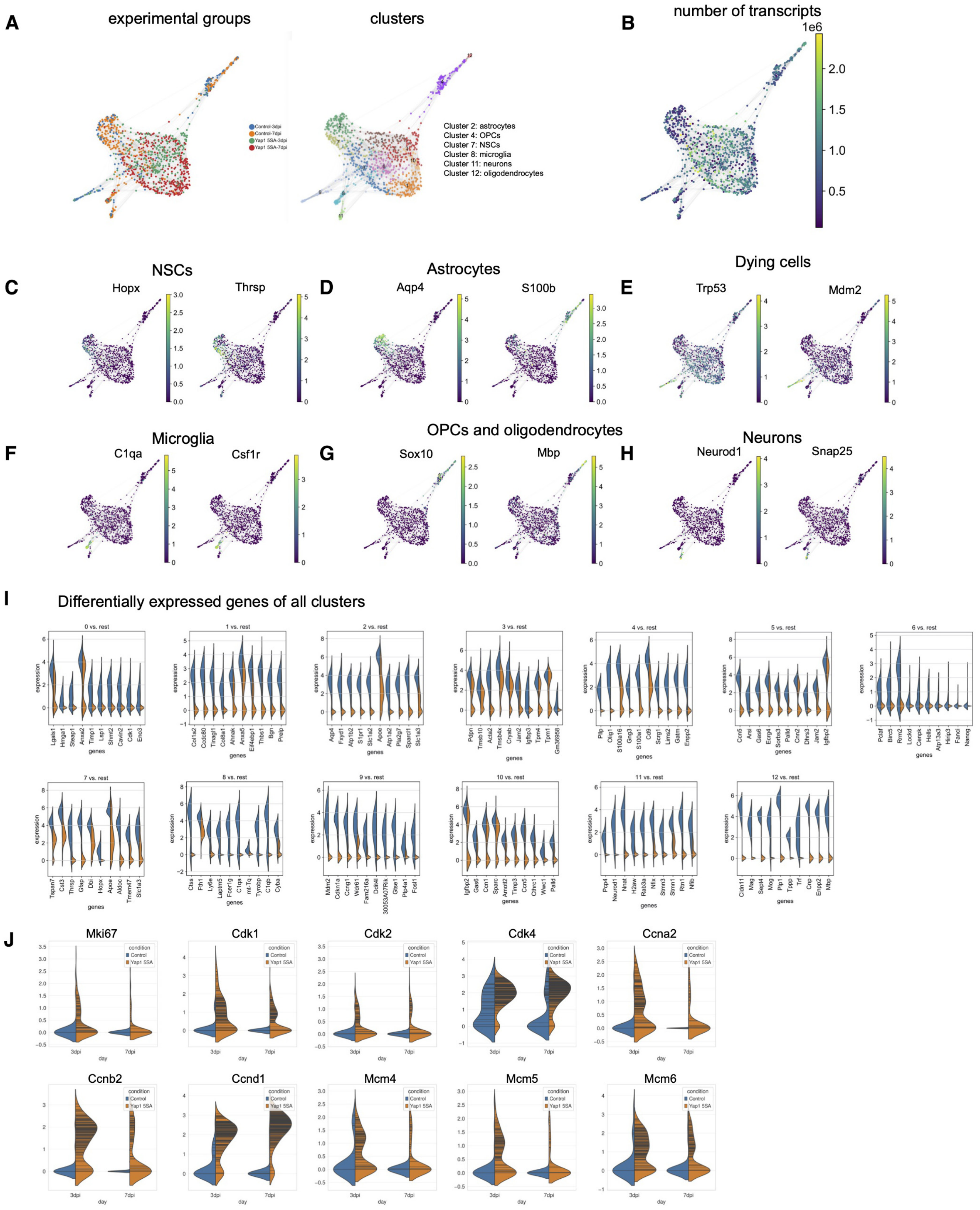
A
experimental groups
clusters
Control-3dpi
Control-7dpi
Yap1 5SA-3dpi
Yap1 5SA-7dpi
Cluster 2: astrocytes
Cluster 4: OPCs
Cluster 7: NSCs
Cluster 8: microglia
Cluster 11: neurons
Cluster 12: oligodendrocytes
B
number of transcripts
1e6
C
NSCs
Hopx
Thrsp
D
Astrocytes
Aqp4
S100b
E
Dying cells
Trp53
Mdm2
F
Microglia
C1qa
Csf1r
G
OPCs and oligodendrocytes
Sox10
Mbp
H
Neurons
Neurod1
Snap25
I
Differentially expressed genes of all clusters
J
Mki67
Cdk1
Cdk2
Cdk4
Ccna2
Ccnb2
Ccnd1
Mcm4
Mcm5
Mcm6
condition
Control
Yap1 5SA
3dpi
7dpi
day

Figure EV4.

Figure EV5. Overexpression of Yap1-5SA in adult NSCs for 30 days *in vivo*.

A Schematic diagram of experimental design. P60 mice were analyzed at 30 days after lentivirus injection of control, wild-type Yap1 and Yap1-5SA.
B Immunofluorescence for GFP and Sox2 in control conditions or overexpression of wild-type Yap1 or Yap1-5SA 30 days after lentivirus injection.
C High magnification images from immunofluorescence in (B). Lower panel shows quantification of Sox2-positive cells among GFP-positive cells in (C). n = 4 mice (biological replicates) in control and Yap1-5SA group; n = 3 mice (biological replicates) in Yap1 WT group.
D Immunofluorescence for GFP and NeuN in control conditions or overexpression of wild-type Yap1 or Yap1-5SA 30 days after lentivirus injection.
E Immunofluorescence for GFP and S100 beta (astrocyte marker) in Yap1-5SA overexpressing mice at 30 days after lentivirus injection.

Data information: Data are represented as mean ± SEM. One-way ANOVA. Scale bars: 100 μm in (B). 10 μm in (C), (D), and (F).

 © 2023 The Authors

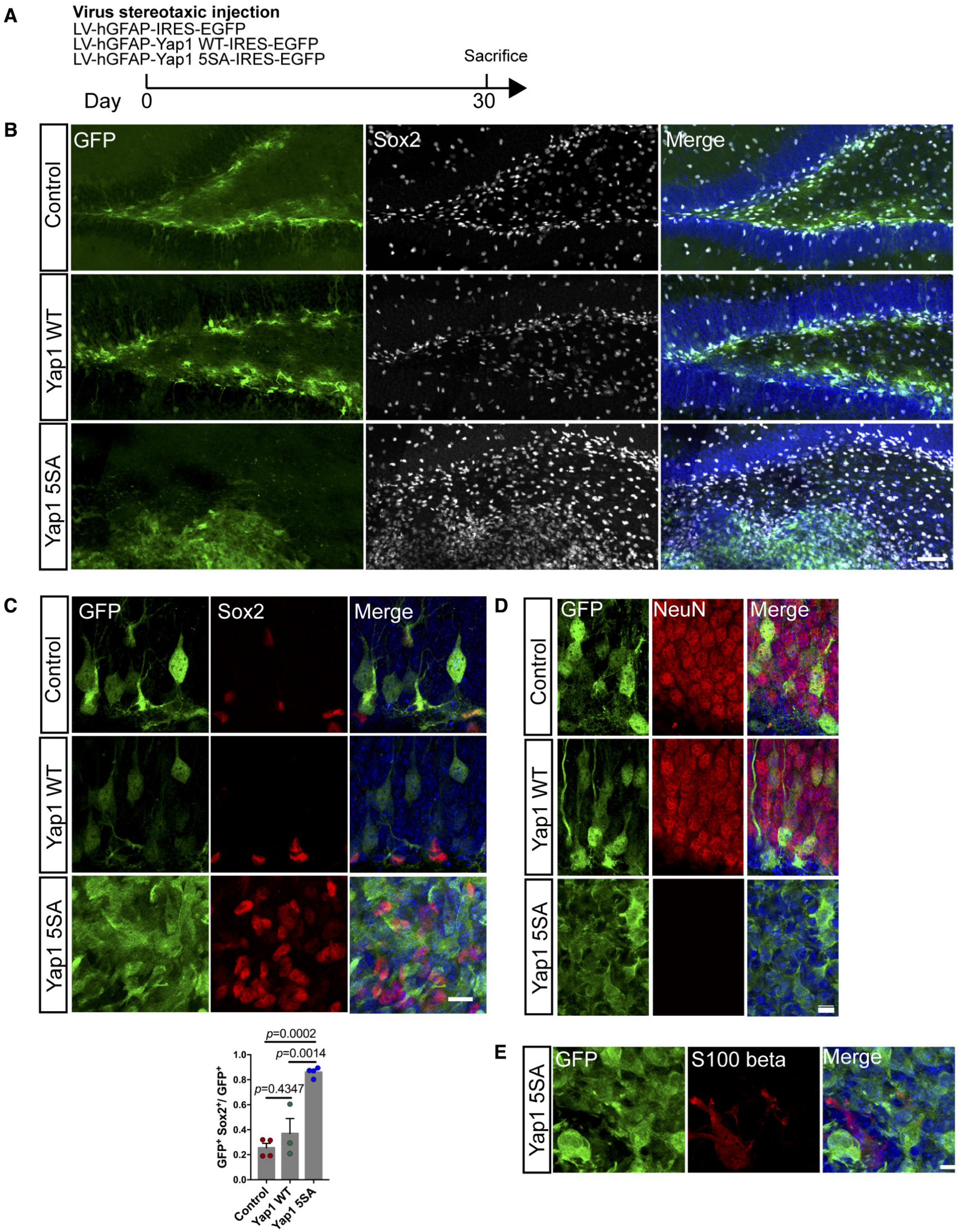
A
Virus stereotaxic injection
LV-hGFAP-IRES-EGFP
LV-hGFAP-Yap1 WT-IRES-EGFP
LV-hGFAP-Yap1 5SA-IRES-EGFP
Sacrifice
Day 0
30
B
Control
Yap1 WT
Yap1 5SA
GFP
Sox2
Merge
C
GFP
Sox2
Merge
Control
Yap1 WT
Yap1 5SA
p=0.0002
p=0.0014
p=0.4347
GFP+ Sox2+/ GFP+
1.0
0.8
0.6
0.4
0.2
0.0
Control
Yap1 WT
Yap1 5SA
D
GFP
NeuN
Merge
Control
Yap1 WT
Yap1 5SA
E
Yap1 5SA
GFP
S100 beta
Merge

Figure EV5.

APPENDIX MATERIALS

Contents

Appendix Figures S1-S4 and Appendix Figure Legends

Appendix Fig S1

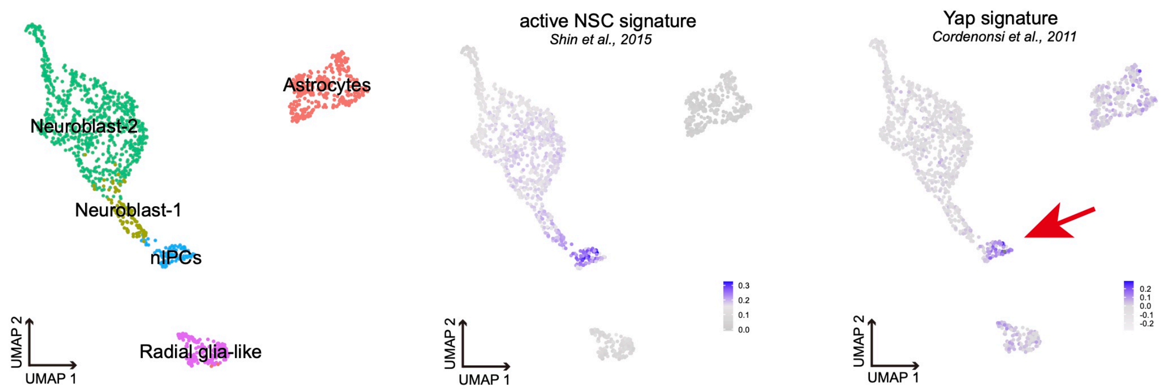

Appendix Fig S1. Yap1 signature is enriched in the proliferative neural progenitors of the mouse hippocampus.
Uniform Manifold Approximation and Projection (UMAP) of single cell transcriptome from published data set comprising NSCs and their early progeny isolated from the dentate gyrus of the adult mouse hippocampus. Yap1 signature is enriched in the same cell population as the active NSC signature. Arrow indicates Yap1 signature in the nIPCs (neural intermediate progenitor cells).

Appendix Fig S2

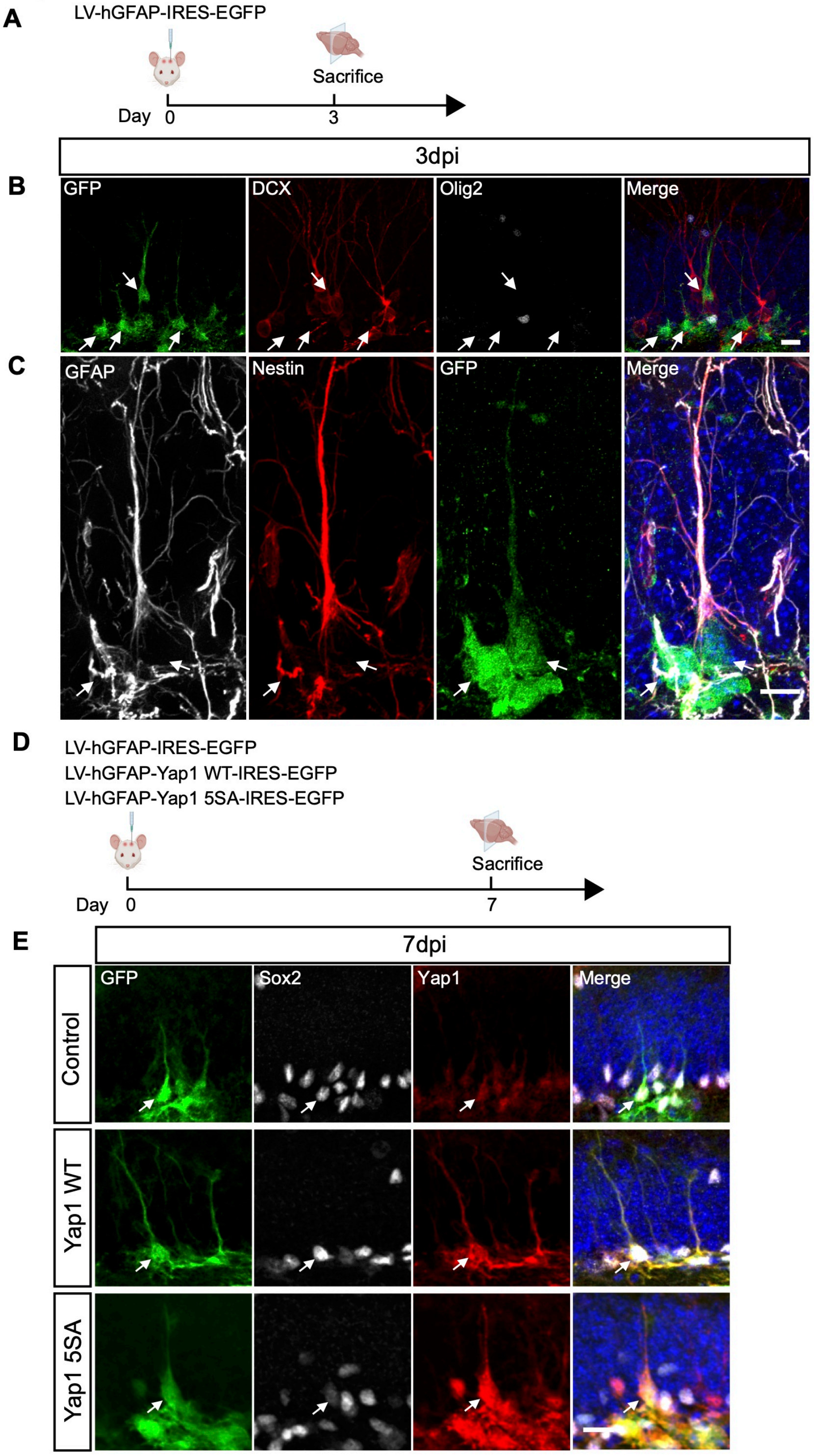

Appendix Fig S2. Targeting of adult NSCs *in vivo* with lentivirus under the control of hGFAP gene regulatory sequence.

A. Schematic diagram of experimental design. P60 mice were analyzed at 3 days after control lentivirus injection.

B. Immunofluorescence for GFP, DCX and Olig2 in coronal brain sections. Arrows indicate adult RGLs in the DG.

C. Immunofluorescence for GFP, Nestin and GFAP in coronal brain sections. Arrows indicate GFAP, Nestin and GFP positive adult RGLs in the DG.

D. Schematic diagram of experimental design. P60 mice were analyzed at 7 days after lentivirus injection of control, wild type Yap1 and Yap1-5SA.

E. Immunofluorescence for GFP, Sox2 and Yap1 in coronal brain sections. Arrows indicate the expression of Yap1 in adult RGLs.

Data information: Scale bars: 20 µm.

Appendix Fig S3

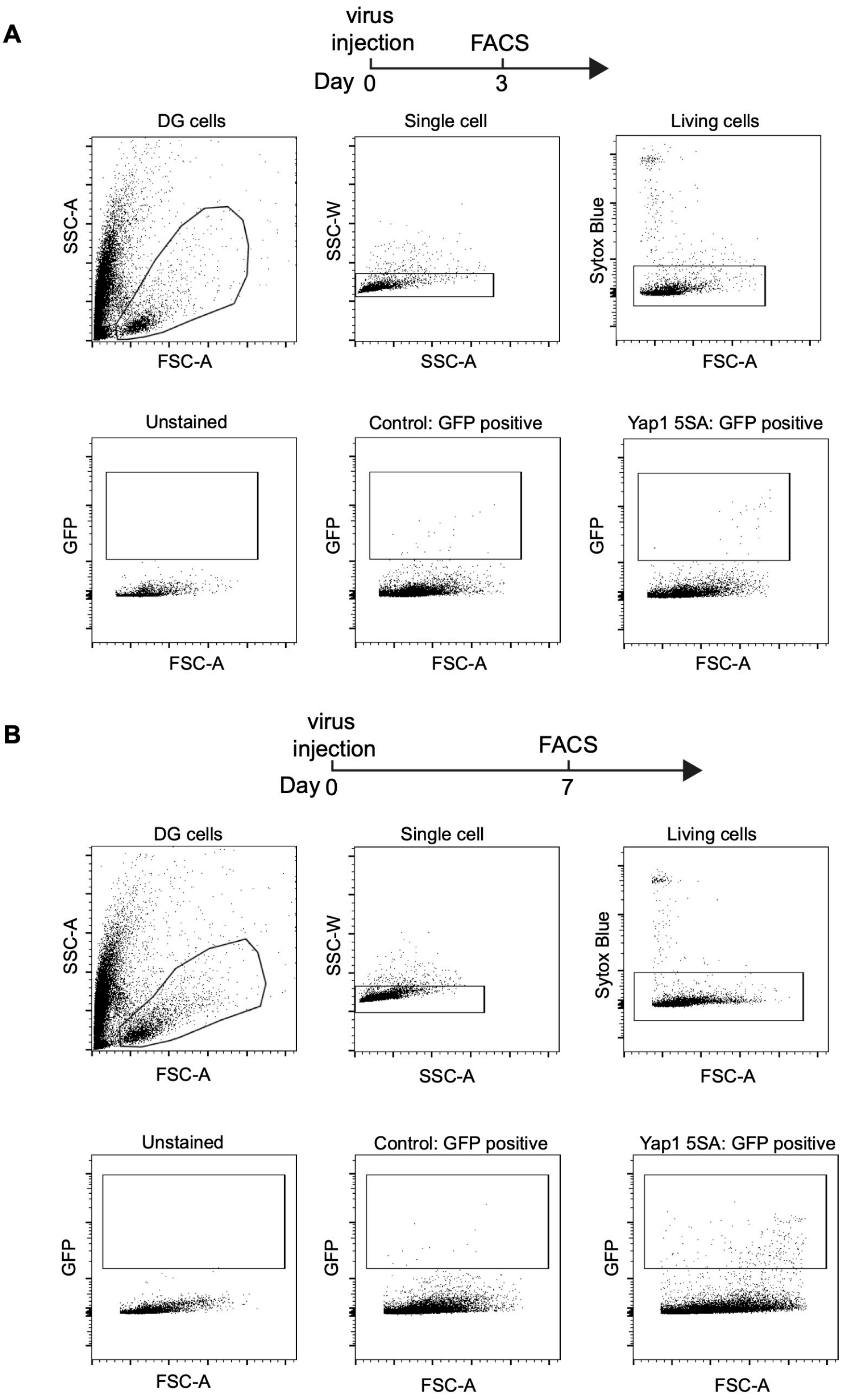

Appendix Fig S3. Gating strategy for FACS at 3 dpi and 7 dpi.

A-B. FACS strategy to obtain GFP positive cells (transduced cells by LV-hGFAP-IRES-EGFP or LV-hGFAP-Yap1-5SA-IRES-EGFP). First gate uses stringent FSC/SSC gating to exclude cellular debris; second excludes cell aggregates to obtain single cells; third excludes dead and dying cells by Sytox Blue; fourth excludes GFP negative cells. GFP positive cells were collected in both control and Yap1 overexpression group at 3dpi and 7dpi.

Appendix Fig S4

A Differentially expressed genes for each cluster after removing dying cells, microglia and oligodendroglial cells

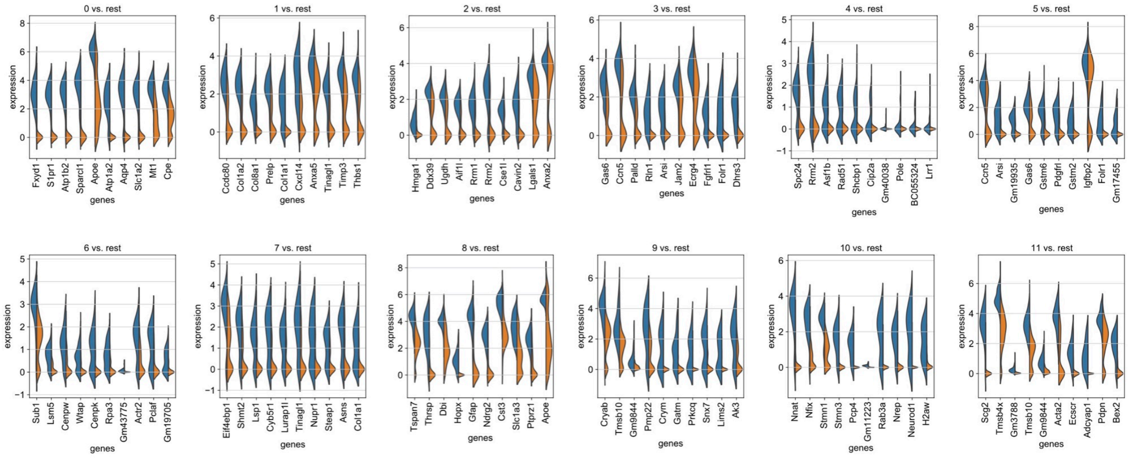

B

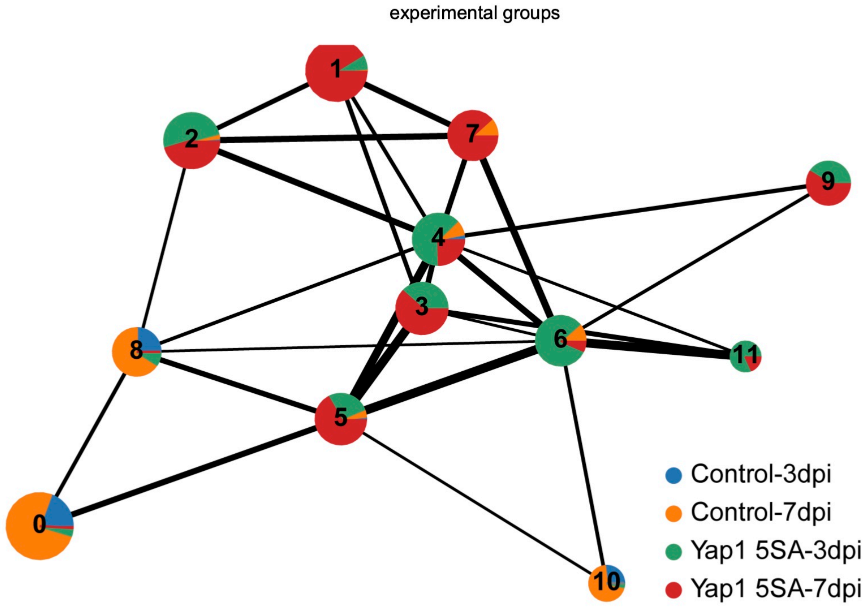

Appendix Fig S4. Re-clustering of the single cell RNA sequencing data after removal of dying cells, microglia and oligodendroglial cells

A. Top 10 markers for each cluster after re-clustering the data.

B. Composition of experimental groups in each cluster are indicated in the plot.
