# Supplementary material for: The transcriptional co‐activator Yap1 promotes adult hippocampal neural stem cell activation
Source: EMBO J. 2023 Apr 21;42(11):e110384. doi: 10.15252/embj.2021110384 (PMC10233373; doi:10.15252/embj.2021110384)
Supplement: Supplementary file 5 — Source Data for Figure 1 [file EMBJ-42-e110384-s008.zip › Figure 1 Source Data/READ ME Figure 1.docx]

**Figure 1**

Panel A

Z-stack confocal image containing:

- Channel 1 Red: Yap1
- Channel 2 Green: Sox2
- Channel 3 Blue: DAPI

Panel B

Maximum intensity projection image:

- Channel 1 Red: Yap1
- Channel 2 Green: GFP (labelling astrocytes)

Panel C

Maximum intensity projection image:

- Channel 1 Red: Yap1
- Channel 2 Green: DCX

Panel D

Maximum intensity projection image:

- Channel 1 Grey: Olig2
- Channel 2 Green: Red: Yap1
- Channel 3 Blue: DAPI

Panel E

Yap1-quiescent NSCs → Z-stack confocal image containing:

- Channel 1: Sox2
- Channel 2: Yap1
- Channel 3: Hopx
- Channel 4: DAPI

Yap1-active NSCs → Z-stack confocal image containing:

- Channel 1: Ascl1
- Channel 2: Yap1
- Channel 3: DAPI

Yap1-NRPs → Z-stack confocal image containing:

- Channel 1: DAPI
- Channel 2: Yap1
- Channel 3: Tbr2

Panel F

Excel file with the quantification of Yap1 intensity values (arbitrary units) in quiescent NSCs, active NSCs and NRPs
