# Supplementary material for: The transcriptional co‐activator Yap1 promotes adult hippocampal neural stem cell activation
Source: EMBO J. 2023 Apr 21;42(11):e110384. doi: 10.15252/embj.2021110384 (PMC10233373; doi:10.15252/embj.2021110384)
Supplement: Supplementary file 6 — Source Data for Figure 2 [file EMBJ-42-e110384-s006.zip › Figure 2 Source Data/READ ME Figure 2.docx]

**Figure 2**

Panel B

Control → Maximum intensity projection image in control animal:

- Channel 1: GFAP
- Channel 2: Yap1
- Channel 3: GFP
- Channel 4: DAPI

Yap1 cKO → Maximum intensity projection image in Yap1 cKO animal:

- Channel 1: GFAP
- Channel 2: Yap1
- Channel 3: GFP
- Channel 4: DAPI

Panel C

Excel file with the quantification of Yap1 intensity in individual NSCs from control and Yap1 cKO animals

Panel D

Control → Maximum intensity projection image in control animal 7 days after virus injection:

- Channel 1: GFAP
- Channel 2: Mcm2
- Channel 3: GFP
- Channel 4: DAPI

Yap1 cKO → Maximum intensity projection image in Yap1 cKO animal 7 days after virus injection:

- Channel 1: GFAP
- Channel 2: Mcm2
- Channel 3: GFP
- Channel 4: DAPI

Panel E

Excel file with the quantification of GFAP+ RGLs among GFP+ in control and Yap1 cKO animals 7 days after virus injection.

Panel F

Excel file with the quantification of Mcm2+ RGLs among GFP+ RGLs in control and Yap1 cKO animals 7 days after virus injection.

Panel G

Control → Maximum intensity projection image in control animal 60 days after virus injection:

- Channel 1: GFAP
- Channel 2: Mcm2
- Channel 3: GFP
- Channel 4: DAPI

Yap1 cKO → Maximum intensity projection image in Yap1 cKO animal 60 days after virus injection:

- Channel 1: GFAP
- Channel 2: Mcm2
- Channel 3: GFP
- Channel 4: DAPI

Panel H

Excel file with the quantification of GFAP+ RGLs among GFP+ in control and Yap1 cKO animals 60 days after virus injection.

Panel I

Excel file with the quantification of Mcm2+ RGLs among GFP+ RGLs in control and Yap1 cKO animals 60 days after virus injection.
