# Supplementary material for: The transcriptional co‐activator Yap1 promotes adult hippocampal neural stem cell activation
Source: EMBO J. 2023 Apr 21;42(11):e110384. doi: 10.15252/embj.2021110384 (PMC10233373; doi:10.15252/embj.2021110384)
Supplement: Supplementary file 7 — Source Data for Figure 3 [file EMBJ-42-e110384-s002.zip › Figure 3 Source Data/READ ME Figure 3.docx]

**Figure 3**

Panel B

Control → Maximum intensity projection image 7 days after control virus injection:

- Channel 1: Sox2
- Channel 2: Ki67
- Channel 3: GFP
- Channel 4: DAPI

Yap1 WT → Maximum intensity projection image 7 days after Yap1 WT virus injection:

- Channel 1: Sox2
- Channel 2: Ki67
- Channel 3: GFP
- Channel 4: DAPI

Yap1 5SA → Maximum intensity projection image 7 days after Yap1 5SA virus injection:

- Channel 1: Sox2
- Channel 2: Ki67
- Channel 3: GFP
- Channel 4: DAPI

Panel C

Excel file with the quantification of Ki67+GFP+Sox2+ cells among GFP+Sox2+ 7 days after control, Yap1 WT and Yap1 5SA virus injection.

Panel D

Excel file with the quantification of Sox2+ cells density 7 days after control, Yap1 WT and Yap1 5SA virus injection.
