# Supplementary material for: The transcriptional co‐activator Yap1 promotes adult hippocampal neural stem cell activation
Source: EMBO J. 2023 Apr 21;42(11):e110384. doi: 10.15252/embj.2021110384 (PMC10233373; doi:10.15252/embj.2021110384)
Supplement: Supplementary file 8 [file EMBJ-42-e110384-s007.pdf]

THE EMBO JOURNAL

The transcriptional co-activator Yap1 promotes adult hippocampal neural stem cell activation

Benedikt Berninger, Wenqiang Fan, Jerónimo Jurado-Arjona, Gregorio Alanis-Lobato, Sophie Péron, Christian Berger, Miguel A. Andrade-Navarro, and Sven Falk
DOI: 10.15252/embj.2021110384

Corresponding author(s): Benedikt Berninger (benedikt.berninger@kcl.ac.uk) , Jerónimo Jurado-Arjona (jjurado@kcl.ac.uk)

Review Timeline:

Submission Date:	11th Jan 22
Editorial Decision:	24th Feb 22
Revision Received:	11th Jan 23
Editorial Decision:	6th Mar 23
Revision Received:	20th Mar 23
Accepted:	27th Mar 23

Editor: Karin Dumstrei

Transaction Report:

(Note: With the exception of the correction of typographical or spelling errors that could be a source of ambiguity, letters and reports are not edited. Depending on transfer agreements, referee reports obtained elsewhere may or may not be included in this compilation. Referee reports are anonymous unless the Referee chooses to sign their reports.)

1st Editorial Decision **24th Feb 2022**

Dear Benedikt,

Thank you for submitting your manuscript to The EMBO Journal. Your study has now been seen by three referees and their comments are provided below.

As you can see from the comments there is an interest in the paper, but it is also clear that the analysis has to be strengthened to consider publication here. Should you be able to extend the analysis along the lines list below then I would be interested in considering a revised version.

I think it would be helpful to discuss the revisions further either via email or video. Let me know when a good time for you would be.

When preparing your letter of response to the referees' comments, please bear in mind that this will form part of the Review Process File, and will therefore be available online to the community. For more details on our Transparent Editorial Process, please visit our website: https://www.embopress.org/page/journal/14602075/authorguide#transparentprocess

I thank you for the opportunity to consider your work for publication. I look forward to discussing the revisions further with you

with best wishes

karin

Karin Dumstrei, PhD
Senior Editor
The EMBO Journal

Instructions for preparing your revised manuscript:

Please see attached PDF for helpful tips on how to prepare the revised version

Guide For Authors: https://www.embopress.org/page/journal/14602075/authorguide

We realize that it is difficult to revise to a specific deadline. In the interest of protecting the conceptual advance provided by the work, we recommend a revision within 3 months (25th May 2022). Please discuss the revision progress ahead of this time with the editor if you require more time to complete the revisions.

Referee #1:

In adult mice, neural stem cells in the dentate gyrus continuously give rise to dentate granule neurons. A central developmental step in adult hippocampal neurogenesis is the activation of the quiescent stem cell. Previous work has identified some molecular pathways (e.g. BMP-signaling, Notch-signaling, Lipid metabolism, ASCL1-dependent transcription) that control neural stem cell activation, but the field is still lacking a complete picture of the molecular network regulating this essential step. This study examines the role of the Hippo-signaling downstream effector YAP1 in stem cell activation using conditional knockout and lentiviral overexpression approach combined with single cell RNA-Seq analyses.The main findings are:

1. activated NSCs feature a YAP1 signature
2. Loss of Yap1 decreases NSC activation after 2 months
3. Increasing YAP1 signaling by overexpression increases NSC activation. Overexpression of a constitutively active mutant of YAP1 induces a glioblastoma like transcriptomic signature in NSCs.

The manuscript addresses an important question in the field of adult neurogenesis (that is the molecular mechanism governing stem cell quiescence and activation) and describes some interesting findings. These findings, however, are not explored in greater depth (impact of YAP1 deletion / YAP1-5SA expression on NSC progeny; tumor formation by Yap1-5SA expressing NSCs;connection of YAP1 to major pathways of stem cell activation) which diminishes the principle significance of the study.
Major comments:
1. The authors provide qualitative evidence that YAP1 is expressed in SOX2+ stem and precursor cells in vivo. Quantification of YAP1 expression in Stem cells / activated stem cells and intermediate precursors would provide better insight in the distribution

of YAP1 expression and would also help to validate the scRNA Seq analysis.
2. The authors describe that decreased activation of stem cell is only observed 60 days after deletion of Yap1. Is this because of technical reasons, i.e., that YAP1 protein is highly stable? The authors describe that YAP1 immunostaining is reduced after 7 days. Analysis of the YAP1 expression in recombined cells at later time points would help to determine the time course of YAP1 protein deletion. If according to such analysis YAP1 stability is unlikely to be the major reason for the delayed stem cell activation phenotype, it would be of high interest to gain more insight into the mechanisms that cause the delay in appearance of the activation phenotype. In vivo scRNA-seq analysis would be ideal; but in vitro analyses (cell biology / bulk sequencing) of YAP1 deleted NSCs may already provide some better insight into the molecular mechanisms.
3. In addition, it would be important to determine the fate of the progeny of YAP1 deleted NSCs, to get insight whether deletion of the transient activity of YAP1 interferes with neuronal differentiation/survival.
4. ASCL1 is considered one of the main drivers of NSC activation. The authors describe that following Yap1-5SA overexpression NSCs do not express significant amounts of ASCL1 mRNA. How are these pathways connected?
5. The findings that Yap1-5SA overexpressing NSCs show higher activation and that they have a glioblastoma like molecular signature are highly interesting. Unfortunately these findings are not explored much further. What is the fate of Yap1-5SA expressing NSCs in the long run? Do Yap1-5SA overexpressing NSCs form tumors? Do Yap1-5SA NSCs generate neurons or astrocytes?

Referee #2:

In this study the authors show the role of the transcriptional activator Yap1 in the transition between the quiescent and the activated state of the neural stem cell (NSC) pool in the adult hippocampus. Using different approaches, they show that conditional deletion of Yap1 in hippocampal NSCs, causes a reduction in the population of proliferating cells in the dentate gyrus. They also show, that overexpression of active Yap1 promotes hippocampal NSCs proliferation. They finally analyse single cell RNA sequencing data from wild-type and Yap1 overexpressing NSCs and show that active Yap1 can induce an aberrant activated NSC signature.
Overall the paper addresses an interesting question in hippocampal NSCs, and could have broader implications about the role of Yap1 in many classes of stem cells. The study is clearly presented, however, the following issues should be addressed:
MAJOR CONCERNS

Authors don´t describe properly in the introduction the radial glia-like cells (RGLs). Although they recognise in the results that this is the major NSCs population in the SGZ and that they can be identified by their localization they don´t talk about these cells in the introduction. Also, I believe that this type of NSCs is not well demonstrated to express or not Yap1. In Fig 1A, they show that Sox2+ cells clearly express Yap1, however they need to combine this staining with GFAP as in Fig. 1B radial cells are not clearly shown. The authors would need to combine Yap1 also with a Hopx marker to clearly define what population of cells are expressing Yap1 in the adult hippocampus. DAPI staining will be useful to better localize Yap1+ cells within the SGZ.

Given that deletion of Yap1 reduces the proportion of activated NSCs in the adult hippocampus, is crucial to determine whether this reduction results in an impairment of neurogenesis. The proportion of DCX+ cells needs to be determined in Yap1 deficient mice. Also in Yap1 5SA mice relative to control.

The analysis of the active versus quiescent NSCs in the SGZ 30 days after inducing the deletion of Yap1 shows a high variability between animals, so to make their conclusion stronger, a higher number of animals should be analysed in Fig EV3B.

I believe that the in vitro study of hippocampal neurospheres in which the authors have promoted the activation of cells (Fig. EV2-E), is not conclusive as low levels of cytoplasmic Yap1 are shown in quiescent cells. An immunostaining for BrdU or Ki67 in combination with Yap1 will help to clarify whether Yap1 is expressed more abundantly in activated cells.

MINOR CONCERNS
- In the introduction several references suggesting a role of Yap1 in brain development or in NSC proliferation are missed. For example, Lavado et al., 2021 (doi: 10.1016/j.ydbio.2021.08.010), Najas et al., 2020 (doi: 10.1016/j.ydbio.2021.08.010) and Han et al., 2020 (doi: 10.1089/scd.2019.0281). These have to be included.

- Given that the authors first analyse the active signature from Shin et al., 2015, this analysis should appear first in Fig EV1. The second panel should be the signature of Yap1 signature.

- In Fig. 2C the way of representing the intensity of Yap1 should take into account the different animals used to perform the study, instead of representing the mean of all cells analysed. These data do not show the variability between animals.

- In Figs 3C and 3D authors should use ANOVA, instead of t-test, to perform statistical comparisons.

- High magnification images of the triple staining for GFP, Sox2 and Ki67 cells in Fig 3B would help to better support the results shown in Fig 3C and D.

Referee #3:

Fan et al. report on the role of YAP1 as a promoter of neural stem cell (NSC) activation in the adult mouse hippocampus. The study is timely and highly relevant for stem cell research as the intracellular regulation of NSC quiescence/activation balance remains poorly known, except for the mandatory role of Ascl1 for quiescent NSC activation.

The experimental approach stems from a smart re-analysis of a previously published transcriptome dataset on adult hippocampal NSCs and progenitors to further develop into well-performed studies on the expression and effects of the loss- and gain-of-function of YAP1, followed by a detailed scRNA-seq analysis of the alteration of gene expression induced by the forced expression of Yap1-5SA, a constitutively active form of YAP1, on hippocampal GFAP-expressing cells.
The techniques are well mastered, the data clear, rigorously analyzed, and very nicely illustrated. Globally, this paper is an example of thoughtful reflection and clear delivery of an important scientific message on YAP1 signaling dysregulation as a driver of glioblastoma-cell signatures. Adding new experimental analyses to explore the regulation of YAP1 signaling in NSCs or YAP1's role in non-NSC populations would distract the paper from its initial goal which focuses on YAP1 effects on the quiescence/activation balance in steady-state and pathological-like conditions.
My suggestion for the authors to improve this paper is to enrich the discussion by commenting on a few additional points:
- YAP1 expression in astrocytes and YAP1's role in NSC activation would suggest that YAP1 also promotes reactive astrogliosis. Literature (PMID: 26679195) nevertheless indicates a repressing role for YAP1, mediated by SOCS3, in this process. Can this be clarified by the authors through examination of their transcriptomic datasets?
- YAP1's relationship with extra and intra-cellular molecules known to regulate cell cycle initiation/progression seems to have been dismissed. For example, YAP1 and VEGF signaling have been recently shown to interact during endothelial cell differentiation (studies from the Ruiz de Almodovar's lab). YAP1 and mTOR collaborate in the context of organ growth (PMID: 23196842) and mTOR is well known to regulate neurogenesis. The authors should open their discussion onto these signaling pathways.
-In the RNA-seq data set, Yap1-5SAexpressing cluster 1 exhibits a prominent expression of mesenchymal cell type-related genes such as Col1a1. How can the authors exclude that this cluster doesn't directly relate to GFAP NSCs, but rather to contaminating GFAP-progenitors of vascular smooth muscle cells/endothelial cells (PMID: 32160776)? Col1a1 is moreover known to be a marker of cerebral perivascular fibroblasts (with PDGFRalpha, see Christer Betsholtz transcriptomic data on brain vascular cells).

No typos or errors in the figure legends have been detected.

Point by point response to referee comments

Referee #1:

In adult mice, neural stem cells in the dentate gyrus continuously give rise to dentate granule neurons. A central developmental step in adult hippocampal neurogenesis is the activation of the quiescent stem cell. Previous work has identified some molecular pathways (e.g. BMP-signaling, Notch-signaling, Lipid metabolism, ASCL1-dependent transcription) that control neural stem cell activation, but the field is still lacking a complete picture of the molecular network regulating this essential step. This study examines the role of the Hippo-signaling downstream effector YAP1 in stem cell activation using conditional knockout and lentiviral overexpression approach combined with single cell RNA-Seq analyses.
The main findings are:
1. activated NSCs feature a YAP1 signature
2. Loss of Yap1 decreases NSC activation after 2 months
3. Increasing YAP1 signaling by overexpression increases NSC activation. Overexpression of a constitutively active mutant of YAP1 induces a glioblastoma like transcriptomic signature in NSCs.
The manuscript addresses an important question in the field of adult neurogenesis (that is the molecular mechanism governing stem cell quiescence and activation) and describes some interesting findings. These findings, however, are not explored in greater depth (impact of YAP1 deletion / YAP1-5SA expression on NSC progeny; tumor formation by Yap1-5SA expressing NSCs; connection of YAP1 to major pathways of stem cell activation) which diminishes the principle significance of the study.

We thank the referee for highlighting the importance of our study, and we hope that now we provide the depth of further analysis this referee rightly requested.

Major comments:

1. The authors provide qualitative evidence that YAP1 is expressed in SOX2+ stem and precursor cells in vivo. Quantification of YAP1 expression in Stem cells / activated stem cells and intermediate precursors would provide better insight in the distribution of YAP1 expression and would also help to validate the scRNA Seq analysis.

As requested by the referee, we now quantified Yap1 expression in quiescent (Hopx+) and activated (Ascl1+) stem cells as well as neuronally-restricted progenitor cells (NRPs, Tbr2+). The data show that activated NSCs express highest levels of Yap1, with NRPs expressing significantly less than the activated NSCs. We modified the text in the results section as indicated below (page 5) and included the new data as part of Fig 1.

To further characterize Yap1 expression dynamics during the lineage progression from quiescent NSCs to early neuronally-restricted progenitor cells, we assessed nuclear Yap1 co-localisation with the quiescent NSC marker Hopx, the activated NSC marker Ascl1 and the neuronal progenitor marker Tbr2, respectively (Fig. 1B and C). This indicated a trend towards increased nuclear Yap1 expression following NSC activation and a significant drop in nuclear Yap1 following neuronal commitment (Fig. 1B and C).

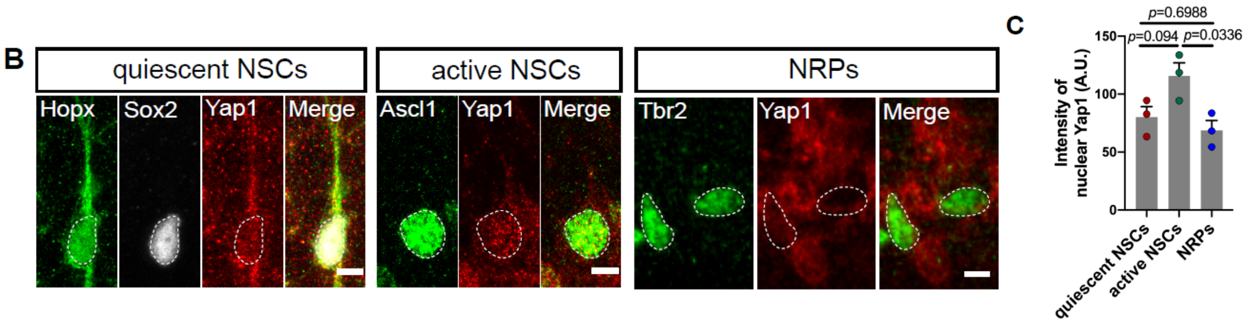

Modification Fig 1. Expression pattern of Yap1 in the DG of the adult hippocampus

B. Representative images of expression of Yap1 in quiescent NSCs (Sox2 and Hopx positive cells), active NSCs (Ascl1 positive cells) and Tbr2-positive cells neuronally-restricted progenitor cells (NRPs). C. Quantification of Yap1 intensity in (B). n= 23 quiescent NSCs from 3 mice, n= 14 active NSCs from 3 mice, n= 30 NRPs from 3 mice.

2. The authors describe that decreased activation of stem cell is only observed 60 days after deletion of Yap1. Is this because of technical reasons, i.e., that YAP1 protein is highly stable?
The authors describe that YAP1 immunostaining is reduced after 7 days. Analysis of the YAP1 expression in recombined cells at later time points would help to determine the time course of YAP1 protein deletion. If according to such analysis YAP1 stability is unlikely to be the major reason for the delayed stem cell activation phenotype, it would be of high interest to gain more insight into the mechanisms that cause the delay in appearance of the activation phenotype. In vivo scRNA-seq analysis would be ideal; but in vitro analyses (cell biology / bulk sequencing) of YAP1 deleted NSCs may already provide some better insight into the molecular mechanisms.

As suggested by the referee, we performed Yap1 expression analysis at a later time point following gene knockout (60 days) (see EV3G). This analysis revealed a similar reduction in the Yap1 intensity (measured in arbitrary units) as observed after 7 days of gene knockout. This indicates that the effect of reduced NSC activation observed after 60 days is unlikely due to a more complete gene knockout, and that effective knockout was eventually achieved already by 7 days. However, we found that Yap1 knockout in cultures of activated hippocampal NSCs resulted in a rapid decline in proliferative activity (Fig. EV2H and I). This might possibly indicate that NSC activation in culture under EGF and FGF2 conditions depends to a larger degree on Yap1 function than NSC activation by physiological mechanisms *in vivo*. While not being able to fully resolve the question of why Yap1 deletion exerts a delayed effect on NSC activation *in vivo*, we discuss now the additional possibility that the effect observed by day 60 could be mediated by indirect mechanisms such as an inflammatory milieu due to Yap1 loss in astrocytes (Huang *et al.*, 2016). We modified the text in the results section as indicated below (pages 6 and 7) and included the new data as part of Fig. EV2.

One possible explanation for this delayed manifestation of Yap1 loss-of-function maybe dependence on other mechanisms for NSC activation. Given the fact that nuclear expression of

Yap1 was even more conspicuous in hippocampal NSCs treated with EGF and FGF2 in vitro, we tested the impact of Yap1 deletion on NSC proliferation in culture by assessing incorporation of the thymidine analogue EdU (Fig. EV2H). This revealed a drastic drop in DNA synthesis following Yap1 loss-of-function (Fig. EV2I). This in vitro data qualitatively supports our observations in vivo, while at the same time suggesting quantitative differences in the dependence of NSC activation on Yap1 function.

Furthermore, we added the following sentence to the discussion (page 13):
Finally, Yap1 loss-of-function may cause a gradual, but indirect decline in NSC activation by promoting an inflammatory response in local astrocytes or NSCs themselves (Huang et al., 2016), which in turn could promote progressive increase in NSC quiescence as shown during aging (Kalamakis et al., 2019).

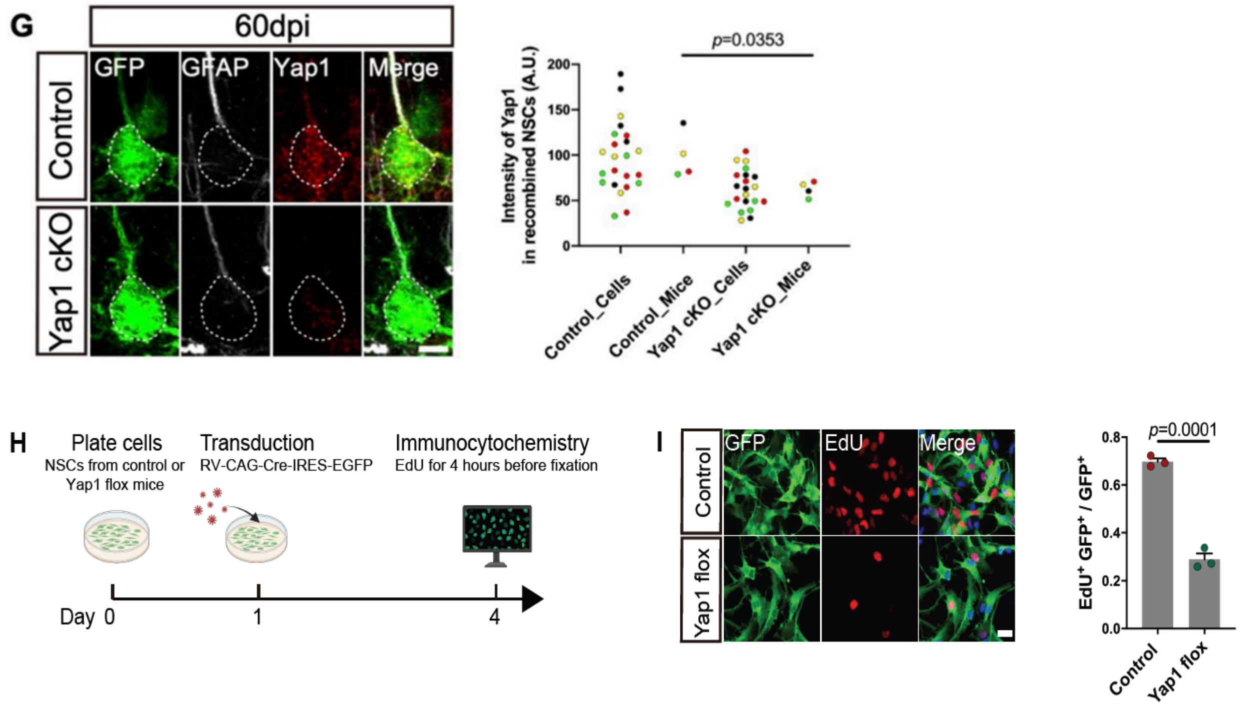

Modification Fig. EV2. Conditional knockout Yap1 in adult NSCs at 30dpi.

G. Immunofluorescence for GFP (recombination reporter), GFAP and Yap1 in control and Yap1 cKO RGLs at 60 days after tamoxifen administration. Quantification of Yap1 intensity in recombined RGLs. Yap1 levels are significantly decreased in Yap1 cKO RGLs at 60dpi. n=4 (23 cells from 4 mice) in control group, n=4 (21 cells from 4 mice) in Yap1 cKO group. H. Schematic diagram of experimental design. I. Immunofluorescence for GFP and EdU indicates that loss of Yap1 efficiently blocks the cell division of adult NSCs, as evidenced by the fact that they did not incorporate the EdU administered during the last 4 hours prior to fixation.

3. In addition, it would be important to determine the fate of the progeny of YAP1 deleted NSCs, to get insight whether deletion of the transient activity of YAP1 interferes with neuronal differentiation/survival.

We performed analysis of DCX expression following Yap1 deletion and found that acquisition of neuronal fate was not overtly affected, neither at 30 days nor 60 days post induction of recombination (Fig. EV2C-F). Accordingly, we modified the text of the results section on page 7:

Interestingly, deletion of Yap1 did not negatively impact lineage progression towards neurogenesis in vivo as Yap1-deleted NSCs gave rise to DCX-positive neurons to a similar extent as control NSCs when assessed at either 30 or 60 days following tamoxifen administration (Fig. EV2C-F).

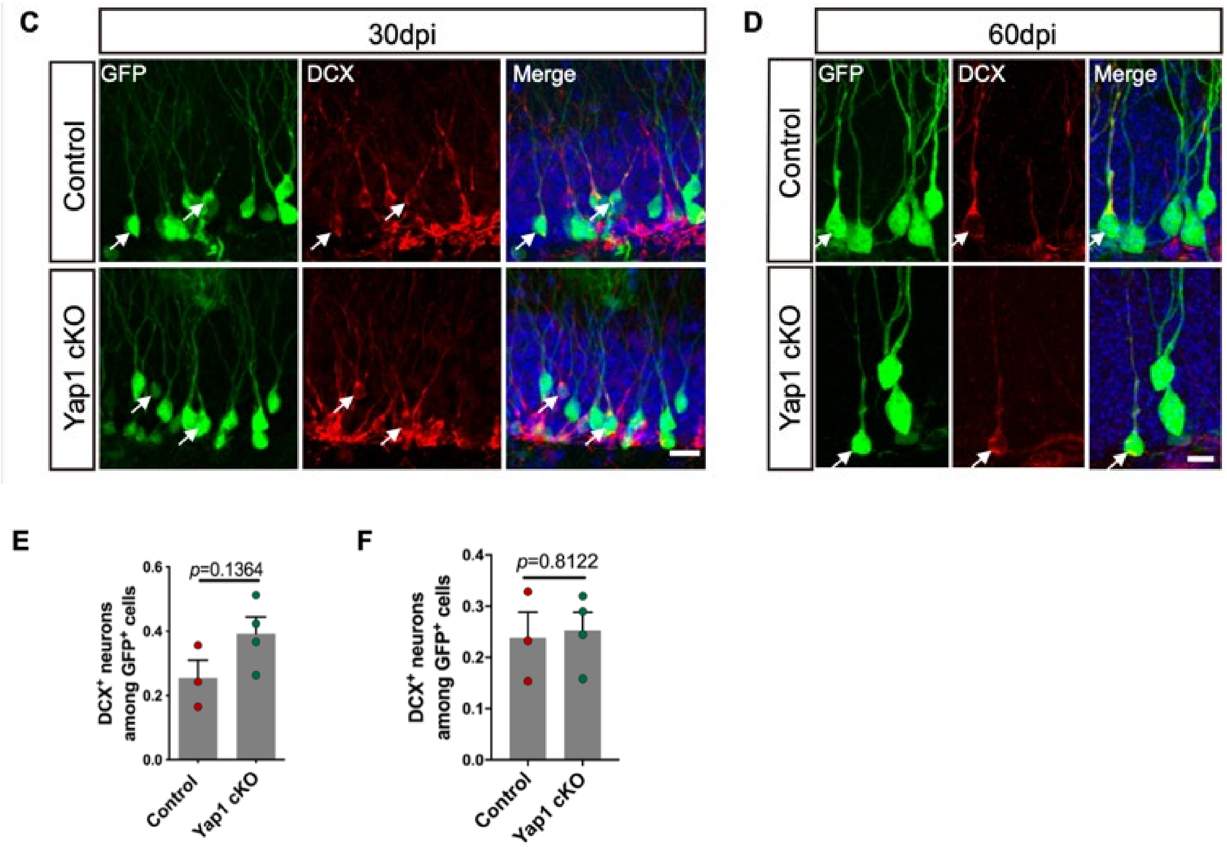

Modification Fig. EV2. Conditional knockout Yap1 in adult NSCs at 30dpi.

C.-D. Immunofluorescence for GFP (recombination reporter) and DCX in control and Yap1 cKO RGLs at 30 days and 60 days after tamoxifen administration. Arrows indicate DCX-positive immature neurons. E.-F. Quantification of the data in (C and D). Loss of Yap1 does not induce significant changes in the number of immature neurons. 30dpi group (E): n=3 mice for control group, n=4 mice for Yap1 cKO group; 60dpi group (F): n=4 mice for both control group and Yap1 cKO group.

4. ASCL1 is considered one of the main drivers of NSC activation. The authors describe that following Yap1-5SA overexpression NSCs do not express significant amounts of ASCL1 mRNA. How are these pathways connected?

This is an interesting question, which we discuss now on page 14:
ASCL1 expression was reported to be negatively correlated with YAP and TAZ expression in large cell neuroendocrine carcinoma and small cell lung cancer cells (Horie et al., 2016). Conversely, proneural factors such as Neurog2 and Ascl1 inhibit Yap1 nuclear localization by triggering phosphorylation of Yap1 at S112 (Zhang et al., 2012). This may suggest that Yap1 and Ascl1 exert a cross-inhibitory effect on each other.

5. The findings that Yap1-5SA overexpressing NSCs show higher activation and that they have a glioblastoma like molecular signature are highly interesting. Unfortunately, these findings are not explored much further. What is the fate of Yap1-5SA expressing NSCs in the long run? Do Yap1-5SA overexpressing NSCs form tumors? Do Yap1-5SA NSCs generate neurons or astrocytes?

To explore the long-term fate of Yap1-5SA expressing cells, we sacrificed animals 30 days after virus injection. While control and even wild type Yap1 expressing cells did not alter the overall architecture of the dentate gyrus and continued to give rise to neurons, Yap1-5SA expressing cells massively disrupted tissue organisation with a concomitant increase in Sox2-positive cells which did not give rise to neurons or astrocytes (Fig. EV5). Accordingly, we modified the text in the results section (pages 10 and 11):

To address the long-term consequences of Yap1 gain-of-function, we finally analysed dentate gyri of animals 30 days after lentivirus-mediated control GFP, wildtype Yap1, or Yap1-5SA expression (Fig. EV5A). While even after 30 days, wildtype Yap1 did not cause any visible alterations in the cytoarchitecture of the dentate gyrus, the latter was massively disrupted following Yap1-5SA gain-of-function (Fig. EV5B), accompanied with a drastic increase in the number of Sox2-positive cells (Fig. EV5B-D). Moreover, whereas control reporter- or wildtype Yap1-expressing cells were capable of undergoing neurogenesis (as assessed by morphology and location in the granule cell layer), Yap1-5SA-expressing cells maintained Sox2 expression and failed to acquire neuronal features (EV9C). Likewise, Yap1-5SA-expressing cells remained negative for the astrocytic protein S100ß indicating that these cells also did not differentiate into astrocytes (Fig. EV5E).

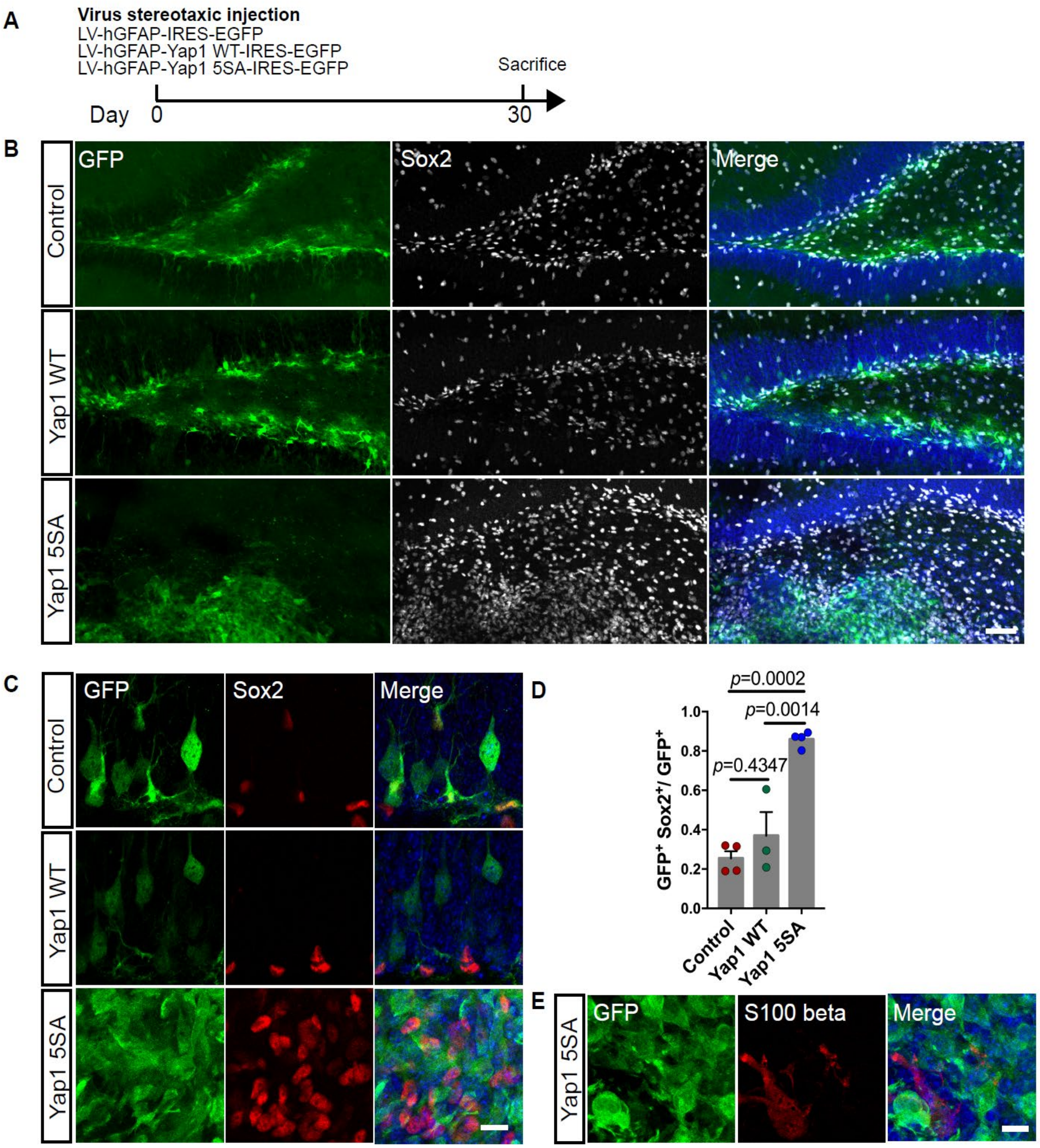

New Fig. EV5. Overexpression of Yap1-5SA in adult NSCs for 30days *in vivo*.

A. Schematic diagram of experimental design. P60 mice were analyzed at 30 days after lentivirus injection of control, wild type Yap1 and Yap1-5SA. B.-C. Immunofluorescence for GFP and Sox2 in control conditions or overexpression of wildtype Yap1 or Yap1-5SA 30 days after lentivirus injection. Low magnification images in (B) and high magnification images in (C). D. Quantification of Sox2 positive cells among GFP positive cells in (C). n=4 mice in control and Yap1-5SA group; n=3 mice in Yap1 WT group. E. Immunofluorescence for GFP and S100 beta (astrocyte marker) in Yap1-5SA overexpressing mice at 30 days after lentivirus injection.

Referee #2:

In this study the authors show the role of the transcriptional activator Yap1 in the transition between the quiescent and the activated state of the neural stem cell (NSC) pool in the adult hippocampus. Using different approaches, they show that conditional deletion of Yap1 in hippocampal NSCs, causes a reduction in the population of proliferating cells in the dentate gyrus. They also show that overexpression of active Yap1 promotes hippocampal NSCs proliferation. They finally analyse single cell RNA sequencing data from wild-type and Yap1 overexpressing NSCs and show that active Yap1 can induce an aberrant activated NSC signature. Overall, the paper addresses an interesting question in hippocampal NSCs, and could have broader implications about the role of Yap1 in many classes of stem cells. The study is clearly presented however, the following issues should be addressed:

We thank the referee for underscoring the importance of our findings to the neural and other stem cell fields.

MAJOR CONCERNS

1. Authors don´t describe properly in the introduction the radial glia-like cells (RGLs). Although they recognise in the results that this is the major NSCs population in the SGZ and that they can be identified by their localization they don´t talk about these cells in the introduction. Also, I believe that this type of NSCs is not well demonstrated to express or not Yap1. In Fig 1A, they show that Sox2+ cells clearly express Yap1, however they need to combine this staining with GFAP as in Fig. 1B radial cells are not clearly shown. The authors would need to combine Yap1 also with a Hopx marker to clearly define what population of cells are expressing Yap1 in the adult hippocampus. DAPI staining will be useful to better localize Yap1+ cells within the SGZ.

To introduce the term radial glia-like cells, we modified the introduction as follows (page 3):
Located in the subgranular zone (SGZ), many of these Sox2-positive NSCs are characterized by a glial fibrillary acidic protein (GFAP)-positive radial process that extends across the granule cell layer towards the molecular layer, lending them a radial glia-like appearance (Seri et al., 2001, Suh et al., 2007). Alongside these radial glia-like NSCs, the SGZ harbors also Sox2-positive non-radial neural progenitor cells shown to be derived from dividing radial glia-like cells and themselves capable of undergoing either symmetric neurogenic divisions or asymmetric divisions, yielding one renewed non-radial cell and one neuronal daughter cell (Pilz et al., 2018).

Furthermore, we performed the expression analyses as requested by the referee. We used Hopx and Ascl1 staining to characterize Yap1 expression in quiescent and active NSCs, respectively. This analysis showed a tendency for higher Yap1 expression in activated NSCs even if this didn't reach statistical significance (Fig 1B and c). In any case, this analysis firmly demonstrated that both NSC populations express Yap1. Accordingly, we modified the text of the results section on page 5:

To further characterize Yap1 expression dynamics during the lineage progression from quiescent NSCs to early neuronally-restricted progenitor cells, we assessed nuclear Yap1 co-localisation with the quiescent NSC marker Hopx, the activated NSC marker Ascl1 and the neuronal progenitor marker Tbr2, respectively (Fig. 1B and C). This indicated a trend towards increased nuclear Yap1 expression following NSC activation and a significant drop in nuclear Yap1 following neuronal commitment (Fig. 1B and C).

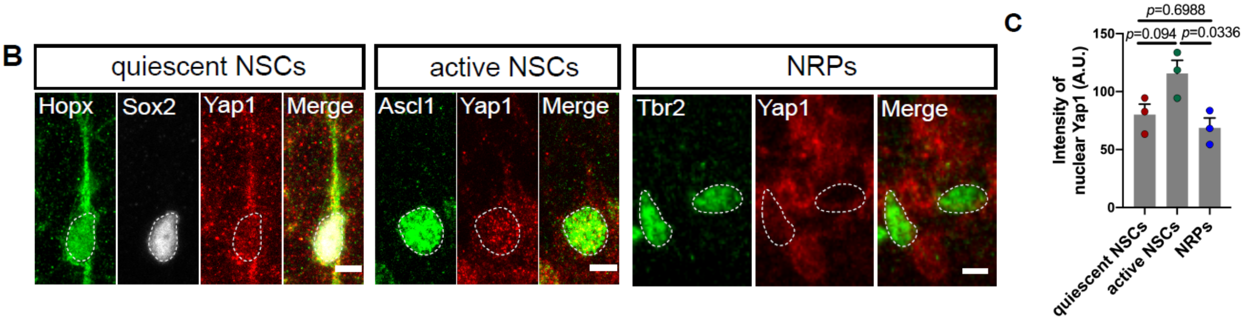

Modification Fig 1. Expression pattern of Yap1 in the DG of the adult hippocampus

B. Representative images of expression of Yap1 in quiescent NSCs (Sox2 and Hopx positive cells), active NSCs (Ascl1 positive cells) and Tbr2-positive cells neuronally-restricted progenitor cells (NRPs). C. Quantification of Yap1 intensity in (B). n= 23 quiescent NSCs from 3 mice, n= 14 active NSCs from 3 mice, n= 30 NRPs from 3 mice.

2. Given that deletion of Yap1 reduces the proportion of activated NSCs in the adult hippocampus, is crucial to determine whether this reduction results in an impairment of neurogenesis. The proportion of DCX+ cells needs to be determined in Yap1 deficient mice. Also, in Yap1 5SA mice relative to control.

As requested by the referee, we analysed the fate of recombined (i.e., Yap1 deleted) cells 30 and 60 days after induction of recombination. We found that reporter-positive cells still gave rise to DCX-positive neurons, with no significant change in number as compared to controls at either time point (Fig. EV2C-F). We therefore modified the text in the results section as follows (page 7):

Interestingly, deletion of Yap1 did not negatively impact lineage progression towards neurogenesis in vivo as Yap1-deleted NSCs gave rise to DCX-positive neurons to a similar extent as control NSCs when assessed at either 30 or 60 days following tamoxifen administration (Fig. EV2C-F).

Furthermore, we analysed the longer term (i.e., 30 days) effect of Yap1-5SA expression on cell fate. This showed that in contrast to control or wild type Yap1 expressing cells, cells expressing the mutant Yap1 no longer could generate neurons, but in contrast, continued to express Sox2 and causing a massive disruption of the cytoarchitecture of the injected dentate gyrus. This new data is shown in Fig. EV5 and are referred to in the results section on pages 10 and 11:

To address the long-term consequences of Yap1 gain-of-function, we finally analysed dentate gyri of animals 30 days after lentivirus-mediated control GFP, wildtype Yap1, or Yap1-5SA expression (Fig. EV5A). While even after 30 days, wildtype Yap1 did not cause any visible alterations in the cytoarchitecture of the dentate gyrus, the latter was massively disrupted following Yap1-5SA gain-of-function (Fig. EV5B), accompanied with a drastic increase in the number of Sox2-positive cells (Fig. EV5B-D). Moreover, whereas control reporter- or wildtype Yap1-expressing cells were capable of undergoing neurogenesis (as assessed by morphology and location in the granule cell layer), Yap1-5SA-expressing cells maintained Sox2 expression and failed to acquire neuronal features (EV9C). Likewise, Yap1-5SA-expressing cells remained negative for the astrocytic protein S100ß indicating that these cells also did not differentiate into astrocytes (Fig. EV5E).

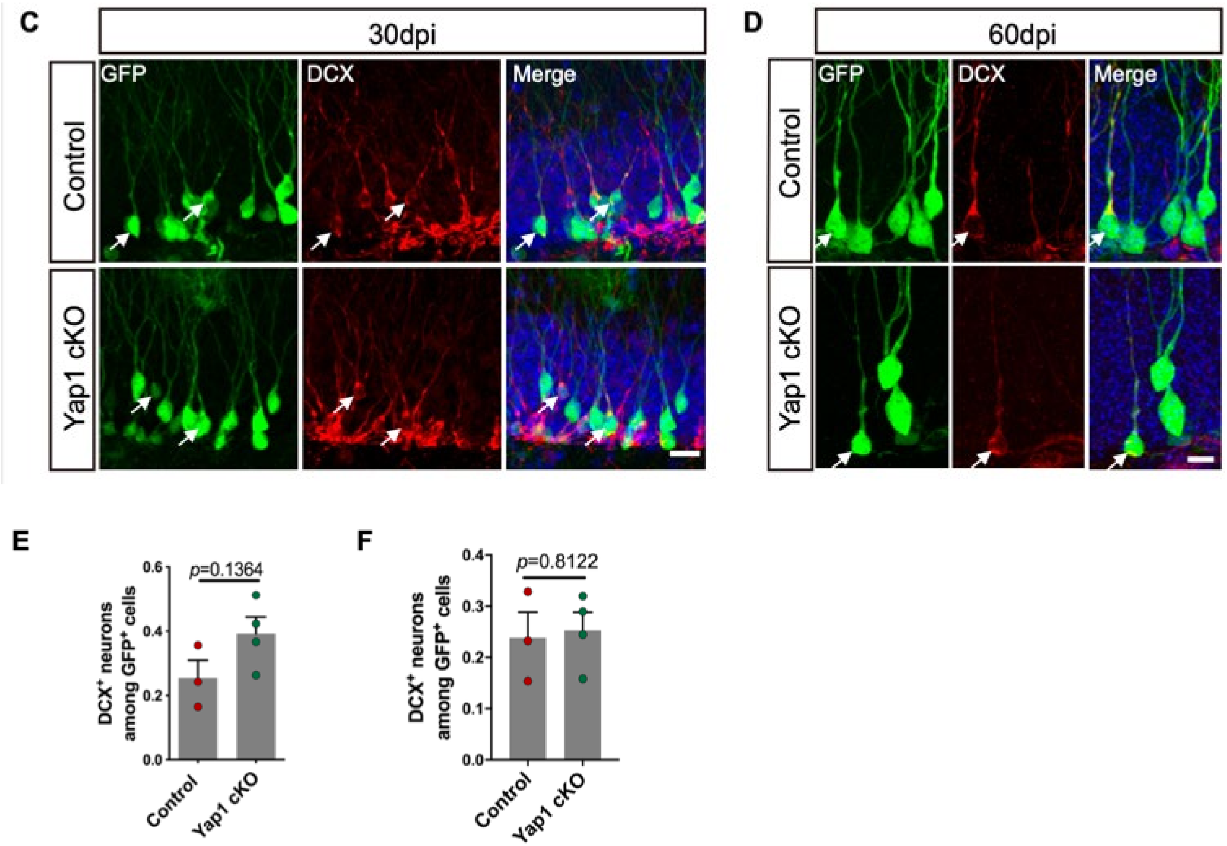

Modification Fig. EV2. Conditional knockout Yap1 in adult NSCs at 30dpi.

C.-D. Immunofluorescence for GFP (recombination reporter) and DCX in control and Yap1 cKO RGLs at 30 days and 60 days after tamoxifen administration. Arrows indicate DCX-positive immature neurons. E.-F. Quantification of the data in (C and D). Loss of Yap1 does not induce significant changes in the number of immature neurons. 30dpi group (E): n=3 mice for control group, n=4 mice for Yap1 cKO group; 60dpi group (F): n=4 mice for both control group and Yap1 cKO group.

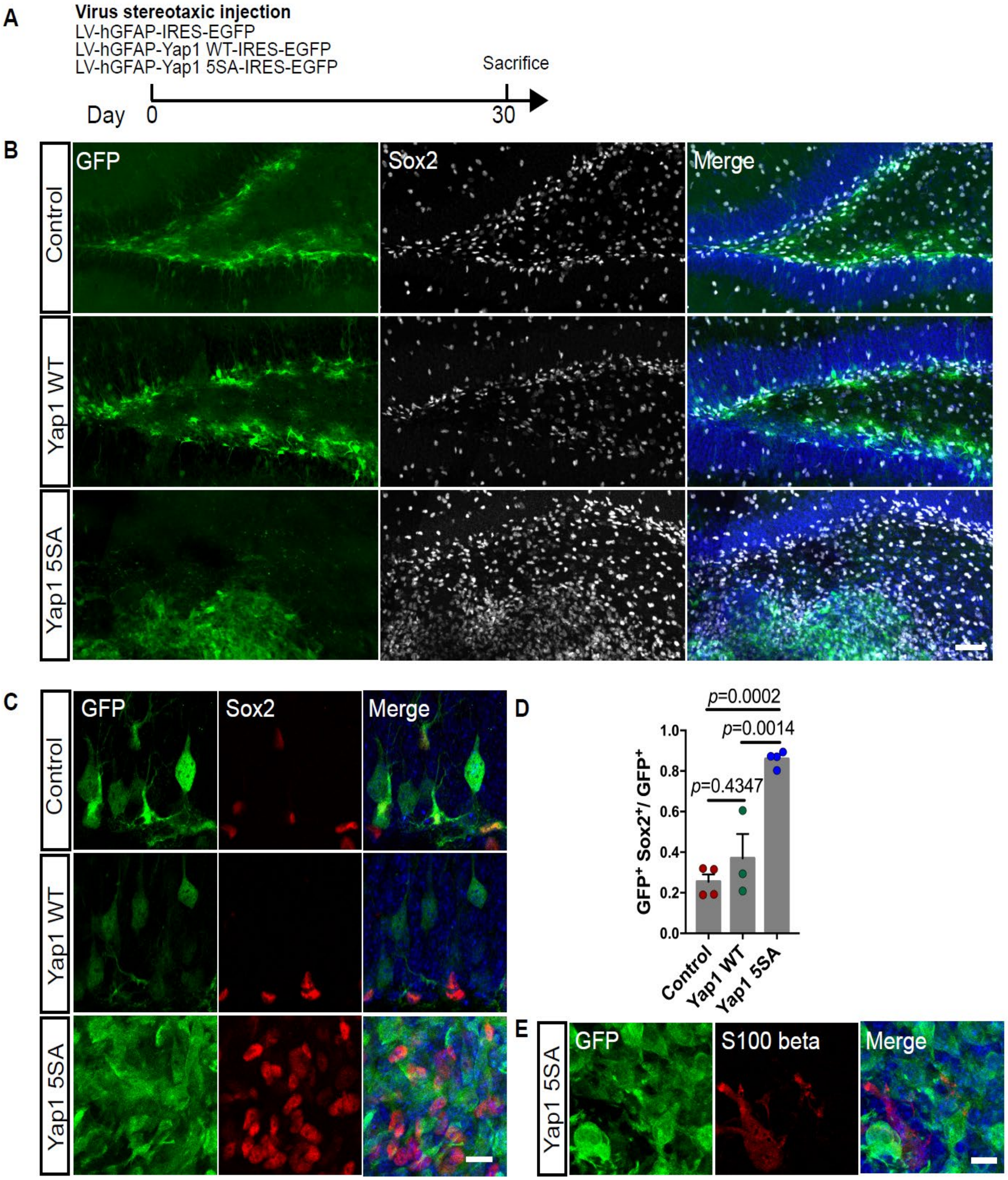

New Fig. EV5. Overexpression of Yap1-5SA in adult NSCs for 30days *in vivo*.

A. Schematic diagram of experimental design. P60 mice were analyzed at 30 days after lentivirus injection of control, wild type Yap1 and Yap1-5SA. B.-C. Immunofluorescence for GFP and Sox2 in control conditions or overexpression of wildtype Yap1 or Yap1-5SA 30 days after lentivirus injection. Low magnification images in (B) and high magnification images in (C). D. Quantification of Sox2 positive cells among GFP positive cells in (C). n=4 mice in control and Yap1-5SA group;

n=3 mice in Yap1 WT group. E. Immunofluorescence for GFP and S100 beta (astrocyte marker) in Yap1-5SA overexpressing mice at 30 days after lentivirus injection.

3. The analysis of the active versus quiescent NSCs in the SGZ 30 days after inducing the deletion of Yap1 shows a high variability between animals, so to make their conclusion stronger, a higher number of animals should be analysed in Fig EV3B.

We agree with the referee that the variability between animals is high. However, in order to detect a potentially significant difference at 30 days following recombination, we would need to increase the number of animals considerably (i.e., at least by doubling the number of animals given the already existing data set), while in case of no significant difference being confirmed, the only gain will be that the standard error will be smaller. Considering cost/benefits, we believe that the gain of knowledge will be too marginal to justify additional animal experiments.

4. I believe that the in vitro study of hippocampal neurospheres in which the authors have promoted the activation of cells (Fig. EV2-E), is not conclusive as low levels of cytoplasmic Yap1 are shown in quiescent cells. An immunostaining for BrdU or Ki67 in combination with Yap1 will help to clarify whether Yap1 is expressed more abundantly in activated cells.

To address the concern of the referee, we performed an *in vitro* culture of adult hippocampal NSCs and after culturing cells in activating conditions (EGF+FGF2), we switched the medium for 2 hours to BMP4 (i.e., quiescent conditions). Under these conditions some cells can still be found in a proliferative state (Ki67-positive). These cells displayed consistently higher intensity of nuclear Yap1 staining than cells negative for Ki67 (Fig. EV1G and H). Accordingly, we modified the text in the results section (pages 5 and 6).

Conversely, when previously activated adult hippocampal NSC cultures were treated with BMP4 for brief period to induce the transition towards quiescence, we observed nuclear Yap1 enrichment in those cells still in cell cycle (i.e., Ki67-positive), while Ki67-negative cells exhibited a significant reduction in nuclear Yap1 (Fig. EV1G and H). This data supports the notion that NSC activation is accompanied by transfer of Yap1 from the cytoplasm to the nucleus, while the reverse process takes place during return to quiescence.

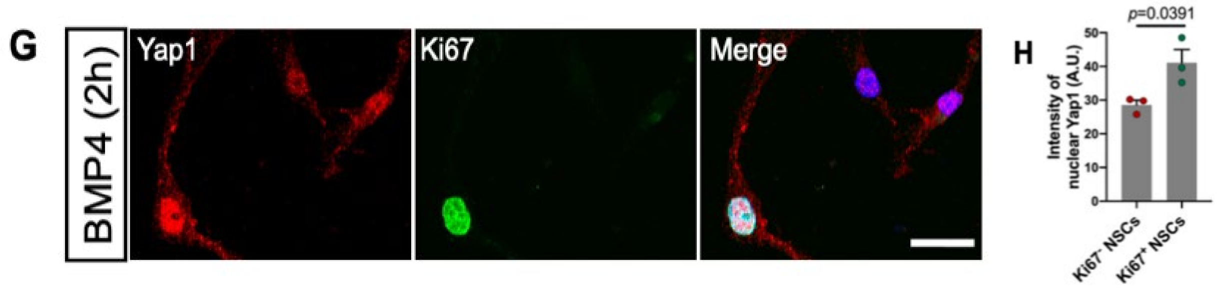

Modification Fig. EV1. Nuclear Yap1 level is increased upon adult NSCs activation.

G. Cultured adult NSCs were treated with BMP4+FGF2 for 2 hours. Immunofluorescence for Yap1 and Ki67 in proliferative and non-proliferative NSCs. H. Quantification of the data in (G). Proliferative NSCs (Ki67-positive) enriched higher level of nuclear Yap1. n=3 (85 Ki67-positive cells and 91 Ki67-negative cells from 3 independent experiments).

MINOR CONCERNS

- In the introduction several references suggesting a role of Yap1 in brain development or in NSC proliferation are missed. For example, Lavado et al., 2021 (doi: 10.1016/j.ydbio.2021.08.010), Najas et al., 2020 (doi: 10.1016/j.ydbio.2021.08.010) and Han et al., 2020 (doi: 10.1089/scd.2019.0281). These have to be included.

We have now included these references (pages 4 and 13, i.e., introduction and discussion).

- Given that the authors first analyse the active signature from Shin et al., 2015, this analysis should appear first in Fig EV1. The second panel should be the signature of Yap1 signature.

We have reversed the order in Appendix Figure S1.

- In Fig. 2C the way of representing the intensity of Yap1 should take into account the different animals used to perform the study, instead of representing the mean of all cells analysed. These data do not show the variability between animals.

To take the different animals used into account, we now present the individual values for each animal in one colour and also depict the average values of all cells analysed per animal. Statistical difference was calculated from the average values.

C

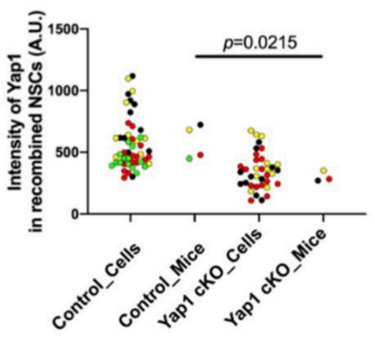

Modification Fig. 2. Yap1 regulates the balance between quiescent and active NSCs.

C. Quantification of Yap1 intensity in (B). Yap1 levels are significantly decreased in Yap1 cKO RGLs at 7dpi. n= 4 (50 cells, 4 mice) in the control group, n= 3 (37 cells, 3 mice) in the Yap1 cKO group.

- In Figs 3C and 3D authors should use ANOVA, instead of t-test, to perform statistical comparisons.

We thank the referee for pointing out our error and corrected it accordingly.

- High magnification images of the triple staining for GFP, Sox2 and Ki67 cells in Fig 3B would help to better support the results shown in Fig 3C and D.

As requested, we now include high magnification images as insets in Fig. 3B

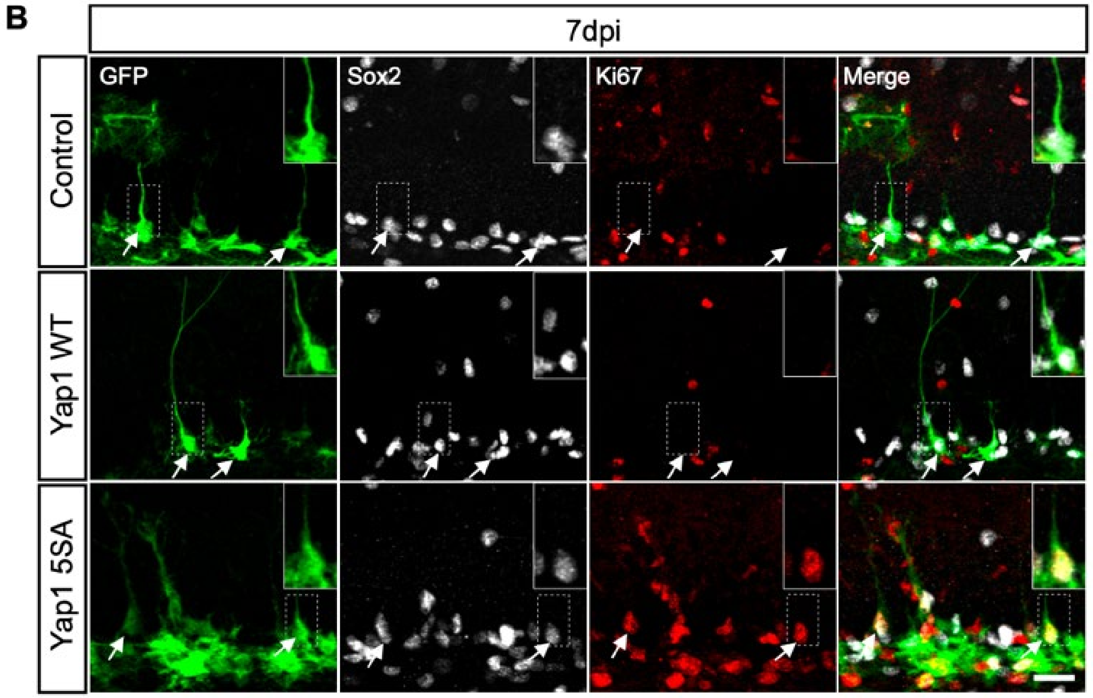

Modification Fig. 3. Yap1 gain-of-function induces adult hippocampal NSC proliferation *in vivo*.

B. Immunofluorescence for GFP, Sox2 (to identify NSCs, arrows) and Ki67 (to identify proliferative cells) in control conditions or overexpression of wildtype Yap1 or Yap1-5SA 7days after lentivirus injection. Insets show a magnified views of the areas outlined by the dashed lines.

Referee #3:

Fan et al. report on the role of YAP1 as a promoter of neural stem cell (NSC) activation in the adult mouse hippocampus. The study is timely and highly relevant for stem cell research as the intracellular regulation of NSC quiescence/activation balance remains poorly known, except for the mandatory role of Ascl1 for quiescent NSC activation.
The experimental approach stems from a smart re-analysis of a previously published transcriptome dataset on adult hippocampal NSCs and progenitors to further develop into well-performed studies on the expression and effects of the loss- and gain-of-function of YAP1, followed by a detailed scRNA-seq analysis of the alteration of gene expression induced by the

forced expression of Yap1-5SA, a constitutively active form of YAP1, on hippocampal GFAP-expressing cells.
The techniques are well mastered, the data clear, rigorously analyzed, and very nicely illustrated. Globally, this paper is an example of thoughtful reflection and clear delivery of an important scientific message on YAP1 signaling dysregulation as a driver of glioblastoma-cell signatures. Adding new experimental analyses to explore the regulation of YAP1 signaling in NSCs or YAP1's role in non-NSC populations would distract the paper from its initial goal which focuses on YAP1 effects on the quiescence/activation balance in steady-state and pathological-like conditions.

We thank the referee for their very positive appraisal of our work.

My suggestion for the authors to improve this paper is to enrich the discussion by commenting on a few additional points:

- YAP1 expression in astrocytes and YAP1's role in NSC activation would suggest that YAP1 also promotes reactive astrogliosis. Literature (PMID: 26679195) nevertheless indicates a repressing role for YAP1, mediated by SOCS3, in this process. Can this be clarified by the authors through examination of their transcriptomic datasets?

The referee raises an interesting question. We did have a look at the expression of SOCS3 in our single cell date (see plot for reviewing purpose only) but could not discern any clear pattern that would indicate massive upregulation of SOCS3 following Yap1-5SA expression. Nonetheless, we modified our discussion to consider the possibility that during reactive gliosis, Yap1 activity may be regulated dynamically rather than persistent to allow for limited SOCS3 expression (page 13).

Also, given its proliferation-promoting effect on hilar astrocytes, it will be interesting to decipher the physiological contribution of Yap1 to the, albeit limited, proliferative activity of parenchymal astrocytes during reactive gliosis. Intriguingly, in the early postnatal cerebral cortex Yap1 loss-of-function was suggested to reduce astrocyte proliferation while at the same time promoting an activated phenotype due to loss of suppressor of cytokine signaling 3, SOCS3, and subsequently increased JAK-STAT signaling in astrocytes (Huang et al., 2016). Thus, reactive gliosis may require dynamic regulation of Yap1 activity to allow for temporally limited cell cycle entry.

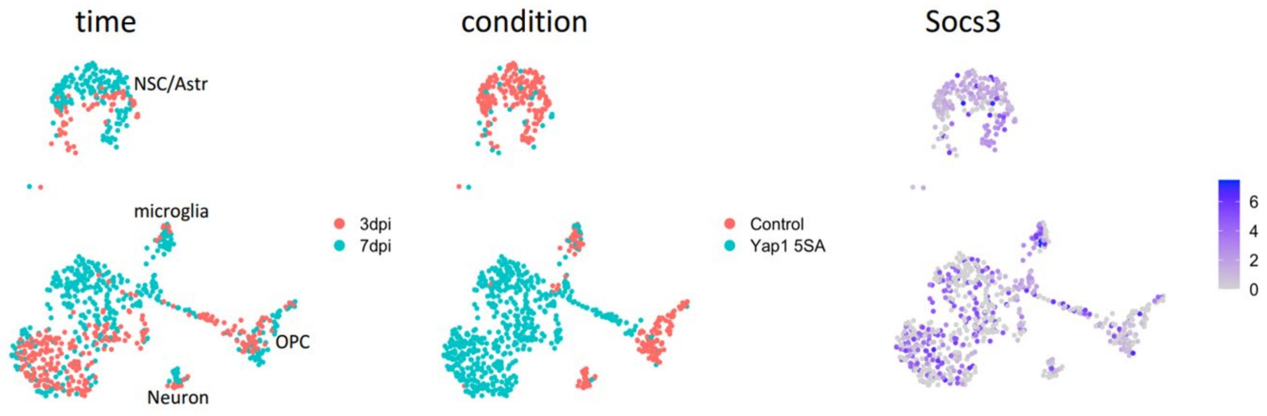

- YAP1's relationship with extra and intra-cellular molecules known to regulate cell cycle initiation/progression seems to have been dismissed. For example, YAP1 and VEGF signaling have been recently shown to interact during endothelial cell differentiation (studies from the Ruiz de Almodovar's lab). YAP1 and mTOR collaborate in the context of organ growth (PMID: 23196842) and mTOR is well known to regulate neurogenesis. The authors should open their discussion onto these signaling pathways.

We modified our discussion accordingly focussing in VEGF signaling (page 12):

Conversely, it is likely that multiple extracellular and intracellular signaling mechanisms converge to activate Yap1 in adult NSCs. Yap1 and Taz have been shown to act as signal transducers of tyrosine kinase receptor pathways such as the VEGF-VEGFR2 pathway in angiogenesis (Wang et al., 2017), and it is conceivable that related tyrosine kinase pathways exert their effects on adult NSCs via Yap1 (Han et al., 2015).

-In the RNA-seq data set, Yap1-5SAexpressing cluster 1 exhibits a prominent expression of mesenchymal cell type-related genes such as Col1a1. How can the authors exclude that this cluster doesn't directly relate to GFAP NSCs, but rather to contaminating GFAP-progenitors of vascular smooth muscle cells/endothelial cells (PMID: 32160776)? Col1a1 is moreover known to be a marker of cerebral perivascular fibroblasts (with PDGFRalpha, see Christer Betsholtz transcriptomic data on brain vascular cells).

While we cannot formally exclude a contribution from GFAP-expressing contaminating cell types, we believe this is rather unlikely as we could not detect any vascular smooth muscle/endothelial or fibroblasts cells among the control cell population (see Fig. EV4). Thus, the contaminating cells would need to have emerged only following Yap1-5SA expression. Since this may be too hypothetical at this stage, we refrained of raising this rather unlikely possibility in our discussion.

In Figure EV2(B-F): It would have been preferable to provide confocal fluorescence images of IHC for YAP1, DAPI and BrdU labeling rather than just for YAP1 and DAPI.

To address the concern of the referee, we performed an *in vitro* culture of adult hippocampal NSCs and after culturing cells in activating conditions (EGF+FGF2), we switched the medium for 2 hours to BMP4 (i.e., quiescent conditions). Under these conditions some cells can still be found in a proliferative state (Ki67-positive). These cells displayed consistently higher intensity of nuclear Yap1 staining than cells negative for Ki67 (Fig. EV1G and H). Accordingly, we modified the text in the results section (page 5).

Conversely, when previously activated adult hippocampal NSC cultures were treated with BMP4 for brief period to induce the transition towards quiescence, we observed nuclear Yap1 enrichment in those cells still in cell cycle (i.e., Ki67-positive), while Ki67-negative cells exhibited a significant reduction in nuclear Yap1 (Fig. EV1G and H). This data supports the notion that NSC activation is accompanied by transfer of Yap1 from the cytoplasm to the nucleus, while the reverse process takes place during return to quiescence.

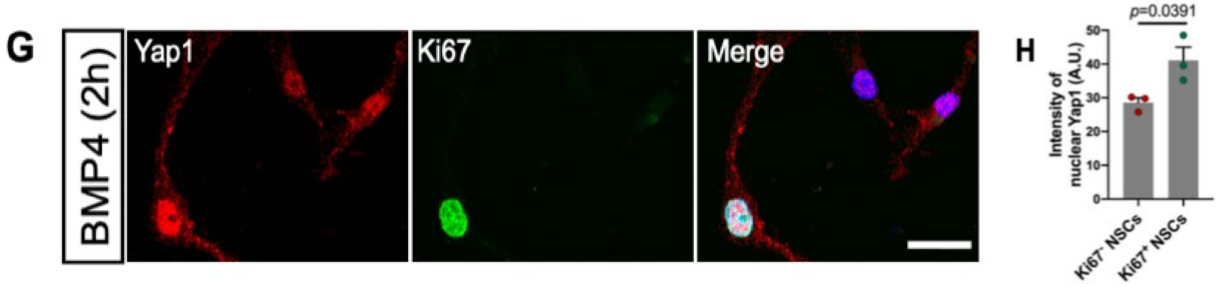

Modification Fig. EV1. Nuclear Yap1 level is increased upon adult NSCs activation.

G. Cultured adult NSCs were treated with BMP4+FGF2 for 2 hours. Immunofluorescence for Yap1 and Ki67 in proliferative and non-proliferative NSCs. H. Quantification of the data in (G). Proliferative NSCs (Ki67-positive) enriched higher level of nuclear Yap1. n=3 (85 Ki67-positive cells and 91 Ki67-negative cells from 3 independent experiments).

Figure 3: More Ki67+ cells does not necessarily mean cell cycle re-entry. A more robust and quantitative way of confirming cycling cells is to perform flow cytometry on primary cultures of hippocampal NSCs with Hoechst and Pyronin Y or qPCR analysis of quiescent NSC- vs activated NSC-genes. Quantification would better support their findings.

To address the question of the referee we took advantage of our single cell data and rendered violin plots of different cell cycle related genes under control conditions or 3 or 7 days of Yap1-5SA expression (Fig. EV4J).

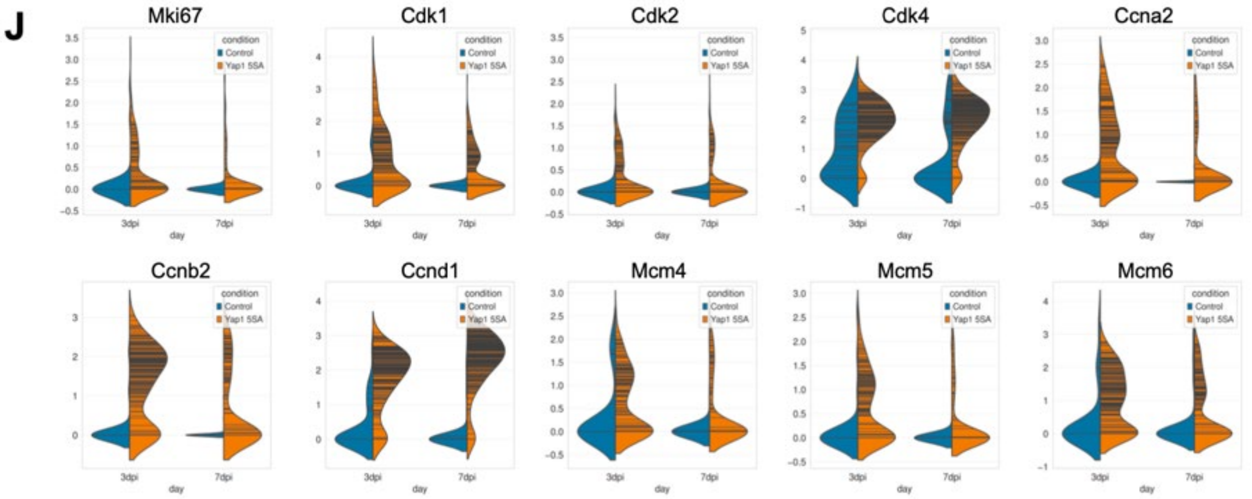

Fig. EV4. Clustering of single cell RNA sequencing data and cell type identification based on differential gene expression analysis.

J. Expression levels of cell cycle related genes at 3 and 7 days after lentivirus injection.

1st Revision - Editorial Decision 6th Mar 2023

Dear Benedikt,

Thank you for submitting your revised manuscript to The EMBO Journal. Your study has now been seen by the three referees.

As you can see from the comments below the referees appreciate the introduced changes. I am therefore very pleased to let you know that we will accept the MS for publication here. Referee #2 has a few points to consider. Before sending you the formal acceptance letter there is just a few things that we need to sort out. When you submit your revised version please also provide a point-by-point response also to the editorial points listed below:

- Please check funding information provided in the online submission system and and MS. They should match => Johannes Gutenberg University Mainz NMFZ2016 listed twice in eJP, and grant number is not the same: NMFZ2016 in eJP and NMFZ in ms file; grant number MSCA-IF-841260 missing in ms file

- COI needs to be re-labeled as Disclosure and competing interests statement

- Please remove the Authors Contributions from the manuscript. The 'Author Contributions' section is replaced by the CRediT contributor roles taxonomy to specify the contributions of each author in the journal submission system. Please use the free text box in the 'author information' section of the manuscript submisssion system to provide more detailed descriptions (e.g., 'X provided intracellular Ca++ measurements in fig Y')

- Figure callouts for Figure 1B and 1C should be after 1A; there are callouts for Figure EV7J, EV8, EV8A, EV9B but only 5 EV figures.

- Page numbers are missing in the appendix file.

- "Supplementary figure legends" should be renamed to "Expanded View figure legends"

- Please also correct the section order

- The synopsis size should be 550 wide by [200-400]

- Please upload a synopsis text. We need a summary statement plus 3-5 bullet points describing the key findings of the MS.

- Please make sure that the deposited datasets are accessible

- Our publisher has also done their pre-publication check on your manuscript. When you log into the manuscript submission system you will see the file "Data Edited Manuscript file". Take a look at the word file and the comments regarding the figure legends and respond to the issues.

- The images EdU & PH3 in Figure EV3 B Control look empty and there is no image noise in Photoshop. Could I ask you to send me the raw data for these panels.

- Also on the note of Source data - we encourage providing source data for the main figures in the MS including for plots. Could I ask you to provide me with source data? Please upload one file/folder (zipped) per figure labelled Source Data figure 1 etc.

That should be all - you can use the link provided below to upload the revised version.

Let me know if you have any further questions.

Best Karin

Karin Dumstrei, PhD
Senior Editor
The EMBO Journal

Guide For Authors: https://www.embopress.org/page/journal/14602075/authorguide

----

Referee #1:

The manuscript convincingly links YAP1 activity to the regulation of adult neural stem cell activation.
As a general comment the revised paper is still rather descriptive and provides very little mechanistic insight, which somewhat lowers the general significance / priority rating by this reviewer.

Referee #2:

The authors diligently and extensively addressed all major concerns and majority of minor concerns. I have only a minor question that they should address.
In the new analysis of neurogenesis after lentivirus-mediated control GFP, wildtype Yap1, and Yap1-5SA expression, there are a few concerns that authors need to resolve. No DCX images or DCX number quantification for Yap1-5SA mice are shown. Also when authors mention this data in the text they refer to figure EV9 but they should refer to figure EV5.
In conclusion I recommend this manuscript to publication after addressing this minor point.

Referee #3:

The authors have satisfactorily answered my questions. The paper has been nicely improved, bringing important new insights into the importance of YAP1 signaling integrity for the maintenance of normal neurogenesis. As so, the revised manuscript deserves publication in the EMBO Journal.

Dear Karin,

Please find below our point-by-point answer to your editorial requests and our response to reviewer #2.

- Please check funding information provided in the online submission system and and MS. They should match => Johannes Gutenberg University Mainz NMFZ2016 listed twice in eJP, and grant number is not the same: NMFZ2016 in eJP and NMFZ in ms file; grant number MSCA-IF-841260 missing in ms file

This has been corrected in the newly revised version of the MS.

- COI needs to be re-labeled as Disclosure and competing interests statement

COI has been changed for the correct title.

- Please remove the Authors Contributions from the manuscript. The 'Author Contributions' section is replaced by the CRediT contributor roles taxonomy to specify the contributions of each author in the journal submission system. Please use the free text box in the 'author information' section of the manuscript submisssion system to provide more detailed descriptions (e.g., 'X provided intracellular Ca++ measurements in fig Y')

Author contribution has been deleted in the revised MS.

- Figure callouts for Figure 1B and 1C should be after 1A; there are callouts for Figure EV7J, EV8, EV8A, EV9B but only 5 EV figures.

Panels in Figure 1 have been rearranged in order to follow the same logic sequence as they are explained in the MS. Callouts for EV figures have been corrected in accordance with the 5 EV contained in the MS.

- Page numbers are missing in the appendix file.

A new appendix PDF file which includes page numbers has been uploaded.

- "Supplementary figure legends" should be renamed to "Expanded View figure legends"

Supplementary figure legends have been renamed as indicated.

- Please also correct the section order

Order has been corrected according with EMBO guidelines and now Figure legends and Expanded View figure legends are at the end of the MS after the references.

- The synopsis size should be 550 wide by [200-400]

The synopsis image has been resized to 550x413

- Please upload a synopsis text. We need a summary statement plus 3-5 bullet points describing the key findings of the MS.

The synopsis text and 4 bullet points have been uploaded in a separate file.

- Please make sure that the deposited datasets are accessible

We have corroborated that the datasets are accessible.

- Our publisher has also done their pre-publication check on your manuscript. When you log into the manuscript submission system you will see the file "Data Edited Manuscript file". Take a look at the word file and the comments regarding the figure legends and respond to the issues.

We thank the publisher for their check to our manuscript and their helpful comments. We have included que suggested modifications in the new version of the revised MS.

- The images EdU & PH3 in Figure EV3 B Control look empty and there is no image noise in Photoshop. Could I ask you to send me the raw data for these panels.

Original picture for figure EV3B is provided. Increasing the intensity of the signal, image noise can be appreciated.

- Also on the note of Source data - we encourage providing source data for the main figures in the MS including for plots. Could I ask you to provide me with source data? Please upload one file/folder (zipped) per figure labelled Source Data figure 1 etc.

We are happy to provide source data. A separate zipped folder per figure has been uploaded in the submission system.

That should be all - you can use the link provided below to upload the revised version.

Let me know if you have any further questions.

Best Karin

Karin Dumstrei, PhD
Senior Editor
The EMBO Journal

Referee #1:

The manuscript convincingly links YAP1 activity to the regulation of adult neural stem cell activation.
As a general comment the revised paper is still rather descriptive and provides very little mechanistic insight, which somewhat lowers the general significance / priority rating by this reviewer.

Referee #2:

The authors diligently and extensively addressed all major concerns and majority of minor concerns. I have only a minor question that they should address.
In the new analysis of neurogenesis after lentivirus-mediated control GFP, wildtype Yap1, and Yap1-5SA expression, there are a few concerns that authors need to resolve. No DCX images or DCX number quantification for Yap1-5SA mice are shown. Also when authors mention this data in the text they refer to figure EV9 but they should refer to figure EV5.
In conclusion I recommend this manuscript to publication after addressing this minor point.

We have now included a new panel Figure EV5, i.e., Fig. EV5D, showing the expression of NeuN in neurons generated from lentivirus transduced NSCs 30 dpi. Control and Yap1WT overexpressing NSCs are able to generate NeuN neurons. In contrast, cells generated by Yap1 5SA overexpressing NSC, do not generate neurons as denoted by the lack of NeuN.

For the reviewer we include here also a force-directed graph showing expression of DCX mRNA which is restricted to cluster 10 comprising neurons and constituted exclusively by control transduced cells. Thus, there is virtually none expression of DCX in Yap15SA cells.

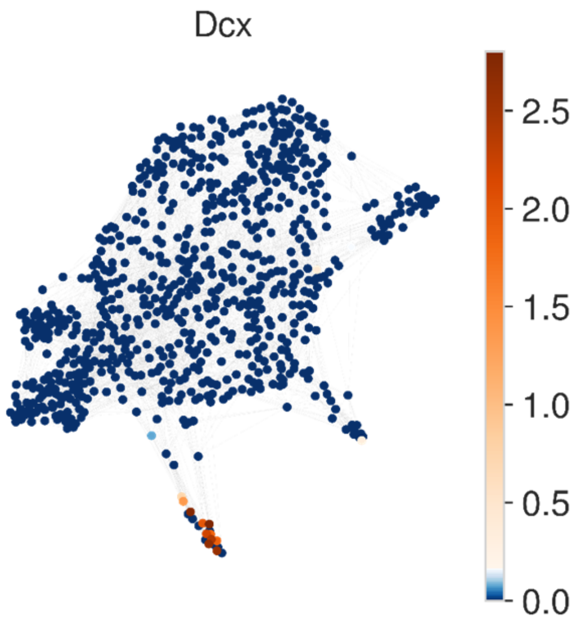

Referee #3:

The authors have satisfactorily answered my questions. The paper has been nicely improved, bringing important new insights into the importance of YAP1 signaling integrity for the maintenance of normal neurogenesis. As so, the revised manuscript deserves publication in the EMBO Journal.

2nd Revision - Editorial Decision 27th Mar 2023

Dear Benedikt,

Thank you for submitting your revised manuscript to The EMBO Journal. I have now looked carefully at everything, and all looks good. I am therefore very pleased to accept the manuscript for publication here.

With best wishes

Karin

Karin Dumstrei, PhD
Senior Editor
The EMBO Journal

Please note that it is EMBO Journal policy for the transcript of the editorial process (containing referee reports and your response letter) to be published as an online supplement to each paper. If you do NOT want this, you will need to inform the Editorial Office via email immediately. More information is available here:
https://www.embopress.org/page/journal/14602075/authorguide#transparentprocess

Your manuscript will be processed for publication in the journal by EMBO Press. Manuscripts in the PDF and electronic editions of The EMBO Journal will be copy edited, and you will be provided with page proofs prior to publication. Please note that supplementary information is not included in the proofs.

You will be contacted by Wiley Author Services to complete licensing and payment information. The required 'Page Charges Authorization Form' is available here: https://www.embopress.org/pb-assets/embo-site/tej_apc.pdf - please download and complete the form and return to embopressproduction@wiley.com

EMBO Press participates in many Publish and Read agreements that allow authors to publish Open Access with reduced/no publication charges. Check your eligibility: https://authorservices.wiley.com/author-resources/Journal-Authors/open-access/affiliation-policies-payments/index.html

Should you be planning a Press Release on your article, please get in contact with embojournal@wiley.com as early as possible, in order to coordinate publication and release dates.

If you have any questions, please do not hesitate to call or email the Editorial Office. Thank you for your contribution to The EMBO Journal.

EMBO Press Author Checklist

Corresponding Author Name: Benedikt Berninger, Jerónimo Jurado-Arjona
Journal Submitted to: EMBO Journal
Manuscript Number: EMBOJ-2021-110384

USEFUL LINKS FOR COMPLETING THIS FORM
The EMBO Journal - Author Guidelines
EMBO Reports - Author Guidelines
Molecular Systems Biology - Author Guidelines
EMBO Molecular Medicine - Author Guidelines

Reporting Checklist for Life Science Articles (updated January
This checklist is adapted from Materials Design Analysis Reporting (MDAR) Checklist for Authors. MDAR establishes a minimum set of requirements in transparent reporting in the life sciences (see Statement of Task: 10.31222/osf.io/9sm4x). Please follow the journal's guidelines in preparing your
Please note that a copy of this checklist will be published alongside your article.

Abridged guidelines for figures
1. Data
The data shown in figures should satisfy the following conditions:
- the data were obtained and processed according to the field's best practice and are presented to reflect the results of the experiments in an accurate and unbiased manner.
- ideally, figure panels should include only measurements that are directly comparable to each other and obtained with the same assay.
- plots include clearly labeled error bars for independent experiments and sample sizes. Unless justified, error bars should not be shown for technical
- if n<5, the individual data points from each experiment should be plotted. Any statistical test employed should be justified.
- Source Data should be included to report the data underlying figures according to the guidelines set out in the authorship guidelines on Data

2. Captions
Each figure caption should contain the following information, for each panel where they are relevant:
- a specification of the experimental system investigated (eg cell line, species name).
- the assay(s) and method(s) used to carry out the reported observations and measurements.
- an explicit mention of the biological and chemical entity(ies) that are being measured.
- an explicit mention of the biological and chemical entity(ies) that are altered/varied/perturbed in a controlled manner.
- the exact sample size (n) for each experimental group/condition, given as a number, not a range;
- a description of the sample collection allowing the reader to understand whether the samples represent technical or biological replicates (including how many animals, litters, cultures, etc.).
- a statement of how many times the experiment shown was independently replicated in the laboratory.
- definitions of statistical methods and measures:
  - common tests, such as t-test (please specify whether paired vs. unpaired), simple χ2 tests, Wilcoxon and Mann-Whitney tests, can be unambiguously identified by name only, but more complex techniques should be described in the methods section;
  - are tests one-sided or two-sided?
  - are there adjustments for multiple comparisons?
  - exact statistical test results, e.g., P values = x but not P values < x;
  - definition of 'center values' as median or average;
  - definition of error bars as s.d. or s.e.m.

Please complete ALL of the questions below.
Select "Not Applicable" only when the requested information is not relevant for your study.

Materials

Newly Created Materials	Information included in the manuscript?	In which section is the information available? (Reagents and Tools Table, Materials and Methods, Figures, Data Availability Section)
New materials and reagents need to be available; do any restrictions apply?	Yes	Materials and Methods

Antibodies	Information included in the manuscript?	In which section is the information available? (Reagents and Tools Table, Materials and Methods, Figures, Data Availability Section)
For **antibodies** provide the following information:<br>- Commercial antibodies: RRID (if possible) or supplier name, catalogue number and or/clone number<br>- Non-commercial: RRID or citation	Yes	Materials and Methods

DNA and RNA sequences	Information included in the manuscript?	In which section is the information available? (Reagents and Tools Table, Materials and Methods, Figures, Data Availability Section)
**Short novel DNA or RNA including primers, probes**: provide the sequences.	Not Applicable	

Cell materials	Information included in the manuscript?	In which section is the information available? (Reagents and Tools Table, Materials and Methods, Figures, Data Availability Section)
**Cell lines:** Provide species information, strain. Provide accession number in repository **OR** supplier name, catalog number, clone number, and/**OR** RRID.	Not Applicable	
**Primary cultures:** Provide species, strain, sex of origin, genetic modification status.	Yes	Materials and Methods
Report if the cell lines were recently **authenticated** (e.g., by STR profiling) and tested for mycoplasma contamination.	Not Applicable	

Experimental animals	Information included in the manuscript?	In which section is the information available? (Reagents and Tools Table, Materials and Methods, Figures, Data Availability Section)
**Laboratory animals or Model organisms:** Provide species, strain, sex, age, genetic modification status. Provide accession number in repository **OR** supplier name, catalog number, clone number, **OR** RRID.	Yes	Materials and Methods
**Animal observed in or captured from the field:** Provide species, sex, and age where possible.	Not Applicable	
Please detail **housing and husbandry conditions**.	Yes	Materials and Methods

Plants and microbes	Information included in the manuscript?	In which section is the information available? (Reagents and Tools Table, Materials and Methods, Figures, Data Availability Section)
**Plants:** provide species and strain, ecotype and cultivar where relevant, unique accession number if available, and source (including location for collected wild specimens).	Not Applicable	
**Microbes:** provide species and strain, unique accession number if available, and source.	Not Applicable	

Human research participants	Information included in the manuscript?	In which section is the information available? (Reagents and Tools Table, Materials and Methods, Figures, Data Availability Section)
If collected and within the bounds of privacy constraints report on age, sex and gender or ethnicity for all study participants.	Not Applicable	

Core facilities	Information included in the manuscript?	In which section is the information available? (Reagents and Tools Table, Materials and Methods, Figures, Data Availability Section)
If your work benefited from core facilities, was their service mentioned in the acknowledgments section?	Yes	Acknowledgements

Design

Study protocol	Information included in the manuscript?	In which section is the information available? (Reagents and Tools Table, Materials and Methods, Figures, Data Availability Section)

If study protocol has been **pre-registered, provide DOI in the manuscript**. For clinical trials, provide the trial registration number **OR** cite DOI.	Not Applicable	
Report the **clinical trial registration number** (at ClinicalTrials.gov or equivalent), where applicable.	Not Applicable	

Laboratory protocol	Information included in the manuscript?	In which section is the information available? (Reagents and Tools Table, Materials and Methods, Figures, Data Availability Section)
Provide DOI OR other citation details if **external detailed step-by-step protocols** are available.	Not Applicable	

Experimental study design and statistics	Information included in the manuscript?	In which section is the information available? (Reagents and Tools Table, Materials and Methods, Figures, Data Availability Section)
Include a statement about **sample size** estimate even if no statistical methods were used.	Yes	Figure legends
Were any steps taken to minimize the effects of subjective bias when allocating animals/samples to treatment (e.g. **randomization procedure**)? If yes, have they been described?	Yes	Quantitative analyses shown in Fig 2E, F, H and I and Fig EV3B were performed double-blind. Indicated in Material and Methods and Results
Include a statement about **blinding** even if no blinding was done.	Yes	Material and Methods, and Results
Describe **inclusion/exclusion criteria** if samples or animals were excluded from the analysis. Were the criteria pre-established?<br><br>If sample or data points were omitted from analysis, report if this was due to attrition or intentional exclusion and provide justification.	Not Applicable	
For every figure, are **statistical tests** justified as appropriate? Do the data meet the assumptions of the tests (e.g., normal distribution)? Describe any methods used to assess it. Is there an estimate of variation within each group of data? Is the variance similar between the groups that are being statistically compared?	Yes	Materials and Methods, and Figure legends

Sample definition and in-laboratory replication	Information included in the manuscript?	In which section is the information available? (Reagents and Tools Table, Materials and Methods, Figures, Data Availability Section)
In the figure legends: state number of times the experiment was **replicated** in laboratory.	Yes	Figures and Figure legends
In the figure legends: define whether data describe **technical or biological replicates**.	Yes	Figure legends

Ethics

Ethics	Information included in the manuscript?	In which section is the information available? (Reagents and Tools Table, Materials and Methods, Figures, Data Availability Section)
Studies involving **human participants**: State details of **authority granting ethics approval** (IRB or equivalent committee(s), provide reference number for approval.	Not Applicable	
Studies involving **human participants**: Include a statement confirming that **informed consent** was obtained from all subjects and that the experiments conformed to the principles set out in the WMA Declaration of Helsinki and the Department of Health and Human Services Belmont Report.	Not Applicable	
Studies involving **human participants:** For publication of **patient photos**, include a statement confirming that consent to publish was obtained.	Not Applicable	
Studies involving experimental **animals**: State details of **authority granting ethics approval** (IRB or equivalent committee(s), provide reference number for approval. Include a statement of compliance with ethical regulations.	Yes	Materials and Methods
Studies involving **specimen and field samples:** State if relevant **permits** obtained, provide details of authority approving study; if none were required, explain why.	Not Applicable	

Dual Use Research of Concern (DURC)	Information included in the manuscript?	In which section is the information available? (Reagents and Tools Table, Materials and Methods, Figures, Data Availability Section)
Could your study fall under dual use research restrictions? Please check biosecurity documents and list of **select agents and toxins** (CDC): https://www.selectagents.gov/sat/list.htm	Not Applicable	
If you used a select agent, is the security level of the lab appropriate and reported in the manuscript?	Not Applicable	
If a study is subject to dual use research of concern regulations, is the name of the **authority granting approval and reference number** for the regulatory approval provided in the manuscript?	Not Applicable	

Reporting

The MDAR framework recommends adoption of discipline-specific guidelines, established and endorsed through community initiatives. Journals have their own policy about requiring specific guidelines and recommendations to complement MDAR.

Adherence to community standards	Information included in the manuscript?	In which section is the information available? (Reagents and Tools Table, Materials and Methods, Figures, Data Availability Section)
State if relevant guidelines or checklists (e.g., **ICMJE, MIBBI, ARRIVE, PRISMA**) have been followed or provided.	Not Applicable	
For **tumor marker prognostic studies,** we recommend that you follow the **REMARK** reporting guidelines (see link list at top right). See author guidelines, under 'Reporting Guidelines'. Please confirm you have followed these guidelines.	Not Applicable	
For **phase II and III randomized controlled trials**, please refer to the **CONSORT** flow diagram (see link list at top right) and submit the CONSORT checklist (see link list at top right) with your submission. See author guidelines, under 'Reporting Guidelines'. Please confirm you have submitted this list.	Not Applicable	

Data Availability

Data availability	Information included in the manuscript?	In which section is the information available? (Reagents and Tools Table, Materials and Methods, Figures, Data Availability Section)
Have **primary datasets** been deposited according to the journal's guidelines (see 'Data Deposition' section) and the respective accession numbers provided in the Data Availability Section?	Yes	Data Availability Section
Were **human clinical and genomic datasets** deposited in a public access-controlled repository in accordance to ethical obligations to the patients and to the applicable consent agreement?	Not Applicable	
Are **computational models** that are central and integral to a study available without restrictions in a machine-readable form? Were the relevant accession numbers or links provided?	Not Applicable	
If publicly available data were reused, provide the respective **data citations in the reference list**.	Yes	reference list
